# Assessing trial representativeness using serious adverse events: an observational analysis using aggregate and individual-level data from clinical trials and routine healthcare data

**DOI:** 10.1186/s12916-022-02594-9

**Published:** 2022-10-28

**Authors:** Peter Hanlon, Elaine Butterly, Anoop S. V. Shah, Laurie J. Hannigan, Sarah H. Wild, Bruce Guthrie, Frances S. Mair, Sofia Dias, Nicky J. Welton, David A. McAllister

**Affiliations:** 1 School for Health and Wellbeing, University of Glasgow, Glasgow, UK; 2 London School of Hygiene and Tropical Medicine, London, UK; 3 Nic Waals Institute, Lovisenberg Diaconal Hospital, Oslo, Norway; 4 Population Health Sciences, Bristol Medical School, University of Bristol, Bristol, UK; 5 Department of Mental Disorders, Norwegian Institute of Public Health, Oslo, Norway; 6 Usher Institute, University of Edinburgh, Edinburgh, Scotland; 7 Centre for Reviews and Dissemination, University of York, York, UK

**Keywords:** Randomised controlled trials, Serious adverse events, Multimorbidity, Epidemiology, Chronic disease

## Abstract

**Background:**

The applicability of randomised controlled trials of pharmacological agents to older people with frailty/multimorbidity is often uncertain, due to concerns that trials are not representative. However, assessing trial representativeness is challenging and complex. We explore an approach assessing trial representativeness by comparing rates of trial serious adverse events (SAE) to rates of hospitalisation/death in routine care.

**Methods:**

This was an observational analysis of individual (125 trials, *n*=122,069) and aggregate-level drug trial data (483 trials, *n*=636,267) for 21 index conditions compared to population-based routine healthcare data (routine care). Trials were identified from ClinicalTrials.gov. Routine care comparison from linked primary care and hospital data from Wales, UK (*n*=2.3M). Our outcome of interest was SAEs (routinely reported in trials). In routine care, SAEs were based on hospitalisations and deaths (which are SAEs by definition). We compared trial SAEs in trials to expected SAEs based on age/sex standardised routine care populations with the same index condition. Using IPD, we assessed the relationship between multimorbidity count and SAEs in both trials and routine care and assessed the impact on the observed/expected SAE ratio additionally accounting for multimorbidity.

**Results:**

For 12/21 index conditions, the pooled observed/expected SAE ratio was <1, indicating fewer SAEs in trial participants than in routine care. A further 6/21 had point estimates <1 but the 95% CI included the null. The median pooled estimate of observed/expected SAE ratio was 0.60 (95% CI 0.55–0.64; COPD) and the interquartile range was 0.44 (0.34–0.55; Parkinson’s disease) to 0.87 (0.58–1.29; inflammatory bowel disease). Higher multimorbidity count was associated with SAEs across all index conditions in both routine care and trials. For most trials, the observed/expected SAE ratio moved closer to 1 after additionally accounting for multimorbidity count, but it nonetheless remained below 1 for most.

**Conclusions:**

Trial participants experience fewer SAEs than expected based on age/sex/condition hospitalisation and death rates in routine care, confirming the predicted lack of representativeness. This difference is only partially explained by differences in multimorbidity. Assessing observed/expected SAE may help assess the applicability of trial findings to older populations in whom multimorbidity and frailty are common.

**Supplementary Information:**

The online version contains supplementary material available at 10.1186/s12916-022-02594-9.

## Background

Randomised controlled trials (hereafter trials) provide the most robust and valid evidence about relative treatment efficacy. However, many patients treated in routine clinical care do not meet trial eligibility criteria; older patients and those with multimorbidity (the presence of two or more conditions) or frailty are often excluded or under-represented [1]. Although not always an explicit exclusion criterion, investigators also routinely exclude people who have concerns over an individual’s ability to manage the burdens of trial participation [2], in order to minimise loss to follow-up [3].

Where such groups are under-represented within trials, the applicability of effect estimates to the wider clinical population is uncertain [4]. Relative treatment effects (e.g. odds ratios) might plausibly differ in older patients or those with frailty or multimorbidity [5]. Even where it is reasonable to assume that relative treatment effects are the same, absolute treatment effects (both benefits and harms), and therefore the balance between risk and benefit, may differ because baseline rates of relevant outcomes vary [4]. Additionally, people excluded from trials may be at greater risk of adverse effects or complications of treatment, particularly in the context of frailty, meaning that assessment of safety based on trials may not be applicable to people receiving treatment in routine care. Thus, it is important to consider the representativeness of trial participants.

Participant representativeness in terms of age, sex and the severity of the target condition can be readily assessed as these characteristics are routinely included in trial reports. However, this is not true for measures of frailty or multimorbidity. We previously examined representativeness in terms of multimorbidity and frailty across a range of industry-funded phase 3 trials [6, 7]. However, this involved the analysis of individual-level participant data which is a complex and time-consuming process, not feasible in most contexts. Consequently, clinicians and guideline developers are usually unable to fully assess trial representativeness.

Trial serious adverse event (SAE) reporting may help address this problem. The primary purpose of collecting SAE data is to detect if the treatments being tested in the trial are harmful. However, any event that is life-threatening leads to death, causes or prolongs hospitalisation, results in serious or lasting impairment or disability, or causes a birth defect is defined as a SAE, regardless of causation, and must be reported [8]. Therefore, where a trial is representative, we would expect the SAE rate to reflect age-sex-specific rates of hospitalisation and death among people with the same condition identified from routine care. SAE rates may therefore be utilised to help assess trial representativeness. In trials for hypertension, we tested this hypothesis, finding that the SAE rates were consistently lower than predicted based on hospitalisation and death rates among people with hypertension in routine care [9]. We also found that although SAE rates were higher in hypertension trials which aimed to be representative of older people, the rates were still lower than in routine care.

This study will examine SAEs in trials and in routine care across 21 index conditions. Using routine healthcare data and trial data, we aim to explore (i) how observed SAE counts in trial populations compare to SAEs (defined as hospitalisations and deaths) for people with the same index condition in routine care, (ii) whether multimorbidity counts will predict hospitalisation and deaths and SAEs similarly in trial and routine care populations and (iii) whether any differences between expected and observed SAEs will be attenuated by accounting for different levels of multimorbidity between trial and routine care populations.

## Methods

### Study design

This observational analysis compares incident SAEs among people enrolled in randomised controlled trials of pharmacological therapies for a range of index conditions to SAEs (defined as deaths or hospitalisations) among people with similar index conditions in routine care. First, we use aggregate data from trials and routine care data from a clinical population with the same index condition and similar age-sex distribution to the trial population to generate an SAE ratio of observed to expected SAEs. Secondly, in trials for which individual participant data were available, we compare observed and expected SAEs in two ways; first where the expected SAEs are based solely on age and sex distribution and secondly where the expected SAEs are additionally estimated using the number of additional long-term conditions (multimorbidity count).

### Data sources and participants

#### Trials—aggregate data

We identified trials registered with clinicaltrials.gov for 21 index conditions. Trials were selected according to a pre-specified protocol (Prospero CRD42018048202) [10]. Trial selection is described in detail elsewhere [7]. Briefly, trials had to be registered with ClinicalTrials.gov; start after 1st January 1990; be phase-2/3, phase-3, or phase-4; include ≥300 participants; have an upper age limit no younger than 60 years; and evaluate pharmacological treatments for one of a range of cardiovascular, respiratory, gastrointestinal, musculoskeletal, metabolic, autoimmune and connective tissue, urological and otolaryngological index conditions (listed in Table 1) [7]. We grouped trials by index condition, defined by the treatment indication as described in the trial registration. For this analysis of SAEs, we then restricted this set of trials to those registered after 2010 (range 2010–2017, mean 2012 and median 2012), as reporting of SAEs on ClinicalTrials.gov was more complete after this date.

**Table 1:** Description of numbers of people in community and participants in trials for each index condition

Index condition	Routine care		Aggregate data trials *N*=483			IPD trials *N*=125		
	Total *N*	Mean age (sd)	Included trials	Total number of participants	Range of mean trial age	Included trials	Total number of participants	Range of mean trial age
Asthma	191,160	45.6 (22.9)	47	74,833	35.2–51.4	4	1084	32.0–50.2
Atrial fibrillation	43,330	74.7 (11.9)	9	12,539	59.3–75.0	1	18,113	72.8
Axial spondyloarthritis	1982	52.4 (15.3)	8	2994	29.9–45.2	2	320	38.0–43.8
Benign prostatic hyperplasia	19,906	72.0 (10.0)	7	4617	60.9–66.5	5	1710	62.2–66.6
Chronic obstructive pulmonary disease	57,378	69.1 (11.6)	94	131,630	61.1–70.8	7	3376	61.0–66.1
Dementia	13,871	82.1 (9.0)	3	2506	73.8–74.4	6	4791	69.0–83.0
Epilepsy	29,554	45.8 (21.0)	8	3715	32.2–41.1	0	-	
Hypertension	310,691	67.0 (12.9)	14	10,380	49.2–70.7	12	6863	51.4–70.9
Inflammatory bowel disease	12514	52.3 (17.8)	7	4086	37.4–44.7	10	4352	36.0–41.9
Myocardial infarction	3510	70.7 (14.1)	11	76,425	58.2–67.0	0	-	
Osteoarthritis	124,521	67.6 (12.7)	4	1794	60.7–62.7	1	1321	63.9
Osteoporosis	38,212	72.8 (12.2)	5	5335	68.8–74.8	7	51,204	53.6–73.2
Parkinson’s disease	4998	74.9 (10.4)	14	5754	61.9–67.5	4	1212	61.0–62.9
Psoriasis	52,810	49.1 (19.0)	24	19,064	43.1–54.2	7	3609	43.6–46.0
Psoriatic arthropathy	3523	54.1 (14.0)	13	5168	47.4–51.9	3	596	45.9–49.0
Pulmonary fibrosis	1465	73.3 (10.9)	4	1962	66.6–70.3	2	1063	66.2–67.7
Pulmonary hypertension	759	60.5 (27.0)	2	1757	48.1–55.7	1	161	52.2
Rheumatoid arthritis	13,809	62.2 (15.5)	29	21,545	46.6–60.7	11	5223	49.0–55.6
Systemic lupus erythematosus	1033	52.8 (15.5)	3	1998	32.1–41.3	2	1129	33.6–39.8
Thromboembolism	9162	66.1 (15.7)	4	8503	40.0–76.4	7	16,959	53.3–55.7
Type 2 diabetes mellitus	82,473	65.3 (13.0)	173	239,662	48.8–74.2	23	19,830	53.5–64.1

#### Trials—individual participant data

From within the list of eligible trials, we identified and accessed individual participant data for trials available via one of two repositories: the Clinical Study Data Request (CSDR) and the Yale University Open Data Access (YODA) project as described in detail previously [7].

#### Routine care comparison

Data from the Secure Anonymised Information Linkage (SAIL) Databank were used to identify a routine care population for each of the trial index conditions. SAIL is a repository of health and administrative data from Wales, includes approximately 70% of the Welsh population, and is nationally representative in terms of distribution of age, sex, and socioeconomic status [7, 11]. We identified people with each of the index conditions from a sample of 2.3 million people registered with a participating general practice between 1st January 2011 and 1st January 2012 (to match the median start date of the trials). Index conditions were identified using diagnostic codes used in UK primary care, as described in detail elsewhere [7].

#### Identifying outcomes

The outcome of interest was incident SAEs (in trials) and incident SAEs in the routine care population – defined as incident hospitalisations/deaths. For the routine care population, SAEs were identified through linkage to the Patient Episode Database for Wales and the National Mortality Registry, respectively. We included all hospital episodes that were coded as ‘urgent’ (as opposed to ‘elective’). For each participant, we assessed incident events occurring between 1st January 2012 and 1st August 2012 (first 6 months available following identification of the index condition, concurrent with the median time of trial registration), de-registration with a participating practice, or death, whichever happened first. This follow-up time was selected to be similar to the follow-up in the included trials (median 26 weeks; interquartile range 12 to 52 weeks). Total observation time was calculated for each individual.

For all registered trials we extracted the number of participants for whom SAEs were reported, the number of persons at risk as reported on ClinicalTrials.gov, and the timeframe for which the events were reported. For trial IPD, event information was identified from the standard adverse event data tables within the CSDR or YODA repositories, and follow-up time was calculated as the number of days from randomisation to the end of follow-up. All IPD trials reported whether an event was classified as serious, however fewer trials provided details of classification (i.e. few trials specified what proportion of SAEs were hospitalisations and deaths versus other causes such as events resulting in impairment or disability). On examining 24 trials providing complete data for death and hospitalisation within YODA, the proportion of SAEs due to hospitalisation or death was generally high (for these 24 trials the proportions were 100% for ankylosing spondylitis, 82% for dementia, 97% for diabetes, 97% for IBD, 96% for psoriasis, 92% for psoriatic arthropathy and 87% for rheumatoid arthritis). SAE ascertainment in the routine care population is likely to be slightly lower than in the trial population.

### Assessing demographics and multimorbidity

For trials, age and sex information was obtained at a summary level from trial registration reporting and directly from individual participant data. For the routine care population, age and sex were obtained from primary care data.

In order to explore the relationship between multimorbidity and SAEs in the trial IPD, we identified twenty-one comorbidities (cardiovascular disease, chronic pain, arthritis, affective disorders, acid-related disorders, asthma/chronic obstructive pulmonary disease, diabetes mellitus, osteoporosis, thyroid disease, thromboembolic disease, inflammatory conditions, benign prostatic hyperplasia, gout, glaucoma, urinary incontinence, erectile dysfunction, psychotic disorders, epilepsy, migraine, parkinsonism and dementia). These were identified using concomitant medication data. Concomitant medication data were used as a surrogate for prevalent multimorbidity as (to maintain patient confidentiality) medical history was frequently redacted in the trial datasets. Medication-based definitions were prespecified following clinical and epidemiological review, and are described in detail elsewhere [7]. Briefly, chronic conditions were grouped to allow identification of broad categories of conditions from medication use (e.g. the use of inhaled bronchodilators was taken to indicate ‘obstructive airways disease’, but we did not attempt to differentiate between asthma and chronic obstructive pulmonary disease). Furthermore, medications which were likely to be used for a diverse range of indications were not used to identify chronic conditions (e.g. we excluded tricyclic antidepressants from the list used to identify affective disorders as these are also used to treat chronic pain). Data from baseline recruitment were used to quantify a total multimorbidity count for each participant in each trial.

For the routine care population, prescription data from the primary care record were used to apply identical medication-based multimorbidity definitions. We applied these definitions to drugs prescribed during 2011, which was treated as ‘baseline’.

### Statistical analysis

The routine care data and trial IPD were both held in different secure data analysis platforms with restrictions on what could be exported. Analysis therefore involved exporting summary statistics and model outputs from each platform. The analyses are presented below relating to the three main aims of the study. A more detailed description of the statistical analyses is given in the supplementary material.

#### Comparison of SAEs in trials (aggregate data) and routine care

This analysis aimed to compare the observed SAEs in trials to SAEs for people with the same index condition in routine care. First, for each index condition, we modelled first hospitalisation or death in routine care using age-adjusted Poisson regression models, stratified by sex. To allow for non-linearity in age, up to two fractional polynomial terms were included. An offset was included to account for differences in person-time. This model therefore allowed us to calculate the predicted SAE rate for each index condition, conditional on age and sex. Second, for each trial with aggregate-level data (*n*=483), we estimated the percentage of trial participants of each sex in one-year age bands based on the age (mean, variance, upper and lower bounds) and sex statistics as reported on clinicaltrial.gov. Third, for each one-year age/sex band, we calculated the expected number of SAEs given the trial size and follow-up time based on the routine care models summing these (weighting by the percentage of participants in each band) to obtain the expected SAEs for the whole trial. Finally, we compared the observed number of SAEs in each trial to the expected number of SAEs, expressed as a ratio (observed/expected SAE ratio). We calculated 95% credible intervals for each trial using sampling methods presented in the statistical appendix. We also estimated the pooled observed/expected ratio at the level of each index condition by fitting a random effects model with a Poisson likelihood treating the expected count as an offset term.

For these analyses, we ignored treatment effects, combining SAEs across all arms, implicitly assuming that the effect of trial interventions on SAEs was ignorable for this set of trials. Following peer review, we conducted the following post hoc exploratory analyses to test this assumption. Although SAE results and design information at the level of trial arms are held within CTG, these are not linked to one another by a unique identifier, so we first harmonised these manually before categorising each trial according to the type of comparison. For 12 trials, the serious adverse event rates were not available at arm level but only as summaries, leaving 471 trials with arm-level SAE information. We then characterised the nature of the comparison in each trial. Of these, in 269 trials there was a placebo arm, in 110 trials all arms within the trial had the same designation (experimental or active but not both) and in 92 trials there were different designations across arms (i.e. experimental versus active). For each trial (for the available comparison), we estimated the log-rate ratio for the difference in SAE rate between arms. We did so by fitting a Poisson regression model with the log person-time (for each arm) as an offset. The person time was estimated similarly as in the main analysis (follow-up time × participants − 0.5 * follow-up time × incident events). We subsequently combined these log-rate ratios in random effects meta-analyses for each index condition according to the type of treatment comparison.

#### Association between multimorbidity count and SAEs in trials (IPD) and routine care

This analysis aimed to compare the association between multimorbidity count and SAEs in trials (using trial IPD) and in routine care. This analysis was limited to trials with IPD and where the total number of SAEs per sex was ≥20 (*n*=60 trials for 11 index conditions). For each sex, we estimated the association between multimorbidity count and SAEs using Poisson regression models, adjusted for age. The log-transformed time to each event or the end of follow-up was included as an offset term. For the trials, the coefficients and standard errors for the comorbidity terms were then meta-analysed in random effects meta-analyses fitted using restricted maximum likelihood estimation. For each index condition, we then plotted the rate ratio (with 95% confidence intervals) for SAEs across the range of multimorbidity counts found in the trials (meta-analysed for each index condition) and in routine care.

#### Comparison of observed and expected trial SAEs before and after accounting for multimorbidity

This final analysis aimed to assess if any differences between expected and observed SAE rates were attenuated by accounting for different levels of multimorbidity between trial and routine care populations. This analysis was based on trial IPD (*n*=125 trials). First, we estimated the expected SAE count for each trial based on age, sex and index condition, using the same models as for the aggregate data (unlike with the aggregate trial analysis, the percentage of participants of each age and sex was directly observed rather than estimated from summary statistics). Next, we fitted further sex-specific models to the routine care dataset for each index condition including, in addition to age, multimorbidity count. Age and multimorbidity count were each modelled using up to two fractional polynomial terms. These models were used to calculate the expected number of SAEs per trial based on the age, sex and multimorbidity count of trial participants. Finally, we calculated the ratio of observed to expected SAEs based on age and sex alone, and based on age, sex and multimorbidity count. The two ratios were then compared for each trial.

All analyses were conducted using R statistical software. All trial-level data, including model outputs, as well as analysis code are provided on the project GitHub repository (https://github.com/dmcalli2/sae_ctg_multicond_public).

## Results

**Fig. 1:**
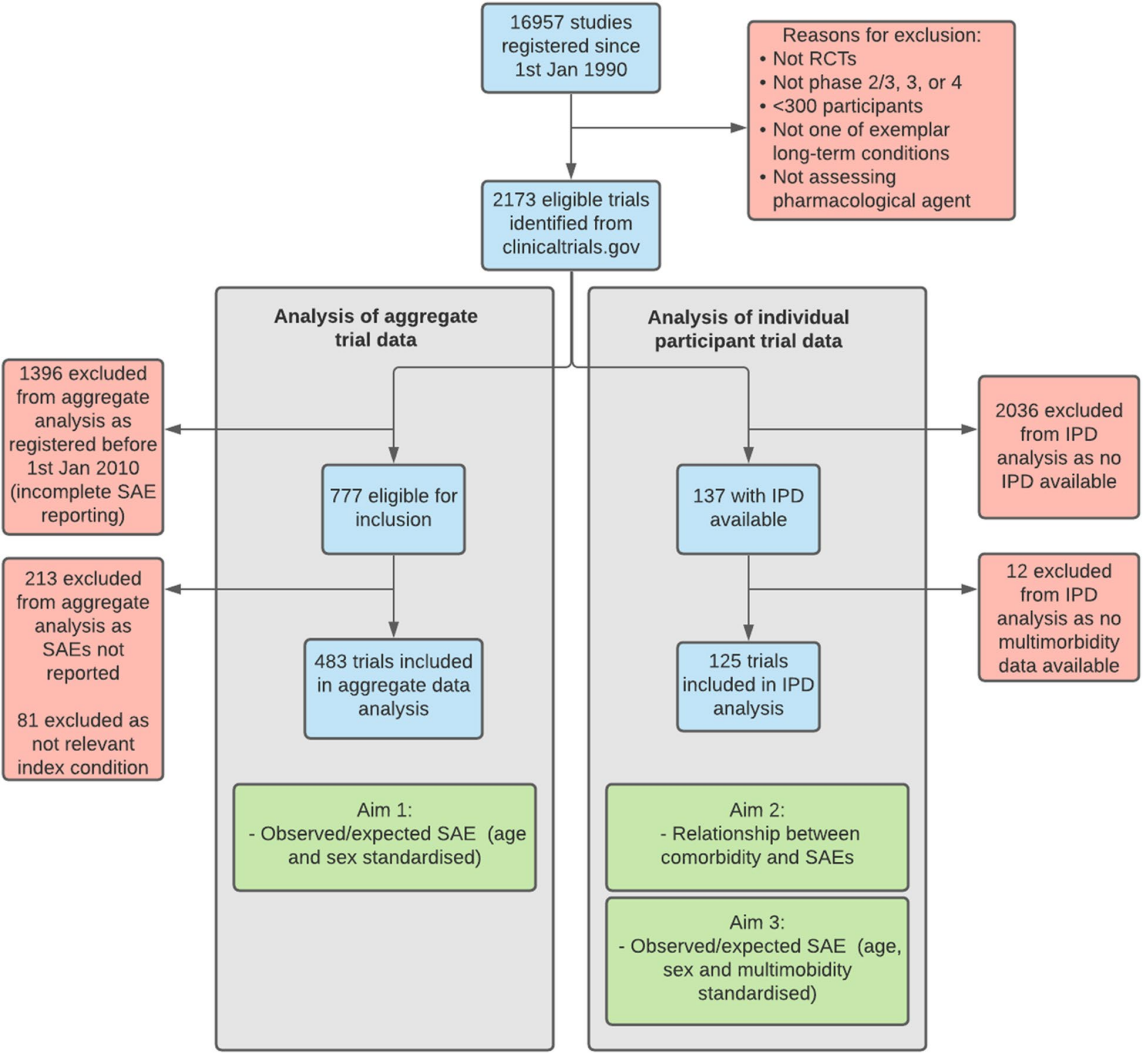
Identification and inclusion of eligible trials

Trial selection is summarised in Fig. 1. Of the 2173 eligible trials identified in our original search, 777 were registered after 1st January 2010. Of these, 578 reported SAE data. 14 were excluded because insufficient information was reported for calculation of SAE counts and a further 81 were excluded as the index condition was not included in our list. We therefore included 483 trials in our analysis of aggregate trial data (n=636,267 participants). We obtained IPD for 125 trials (*n*=122,069 participants) from the CSDR and YODA repositories (trials for which sponsors made IPD available to third party researchers, for which we did not apply a cut-off date of 2010 given that there are relatively few trials for which IPD are available), which were included in subsequent analyses of multimorbidity count. 42 trials were included in both the IPD and aggregate sets. Trials for each of the 21 index conditions are summarised in Table 1, with individual trial summary data shown on the project GitHub repository ( https://github.com/dmcalli2/sae_ctg_multicond_public). This table also shows the total number and mean age of people with each of the index conditions in the routine care sample of 2.3M people registered with a SAIL practice during 2011.

### Comparison of SAEs in trials (aggregate data) and routine care

**Fig. 2:**
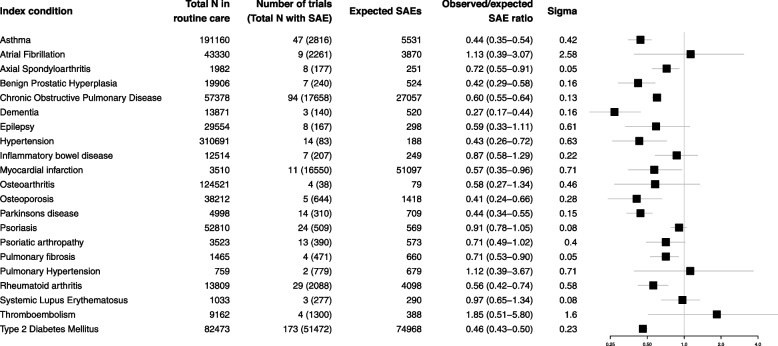
This figure displayed the pooled observed/expected SAE ratio for each of the index conditions. It also shows the number of people in the routine care cohort with each index condition, the number of trials with aggregate data and the total number of SAEs

For each of the index conditions, the observed/expected SAE ratio for each index condition is shown in Fig. 2 pooled across trials. For 18 of the 21 index conditions, the SAEs were lower than that expected; for 12 of these the 95% confidence intervals did not include the null. COPD was the index condition with the median observed/expected SAE ratio (0.60; 95% CI 0.55–0.64), Parkinson’s disease and inflammatory disease were at the 25th and 75th centiles respectively (0.44; 95% CI 0.34–0.55 and 0.87; 0.58–1.29 respectively). The most extreme ratio was seen for dementia (0.27; 95% confidence interval 0.17–0.44) indicating that the rate of SAEs in these trials was around a quarter of that seen in routine care. For three out of 21 conditions, the observed/expected SAE ratio was >1, and for each of these, the confidence intervals were wide and included the null (pulmonary hypertension 1.12 (0.39–3.67), atrial fibrillation 1.13 (0.39–3.07), and thromboembolism 1.85 (0.51–5.80)).

Considerable variation in the observed/expected SAE ratio was apparent between trials within the same index condition. Trial level estimates are shown in Fig. 3 for the six index conditions with the greatest number of trials (all other index conditions are shown in the supplementary appendix, Figs. S1–S21). Taking type 2 diabetes as an example, although the pooled ratio of observed/expected SAE was less than half (0.46 (95% CI 0.43–0.50)), for some trials, it was close to unity.

**Fig. 3:**
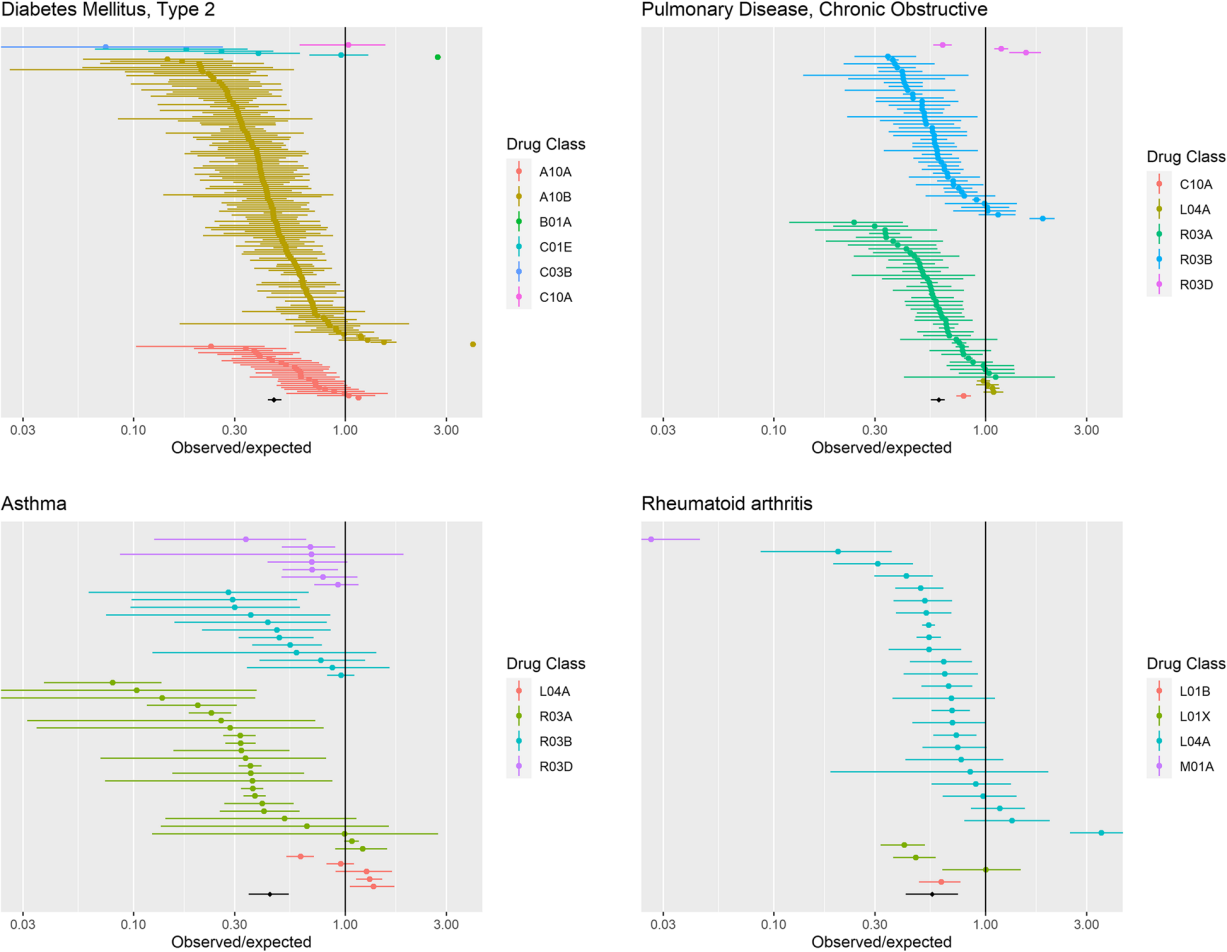
This figure shows the ratio of observed/expected serious adverse event rates in aggregate data trials. Four selected index conditions with the largest number of eligible trials are displayed here, with the remaining conditions displayed in the supplementary appendix. The point-estimate and 95% confidence interval for the ratio for each trial is shown by the coloured points and bars, respectively. Different drug classes are separated by colour (full key displayed in supplementary appendix). The pooled ratio and 95% confidence intervals meta-analysed across all trials within each index condition is shown by the black point and line at the bottom of each plot. Findings are based on analysis of aggregate trial data from ClinicalTrials.gov (index condition, trial drug, age, sex, SAEs and follow-up) for the observed rate and individual patient data from SAIL was used to calculate the expected rate

These analyses pooled SAEs from the treatment and control arms of each trial, assuming that SAE rates would be similar across arms. Post-hoc analyses comparing SAE rates across trial arms, conducted to explore this assumption, are shown in the supplementary appendix. For 471 trials for which trial arm-level data was available, the SAEs were generally similar across trial arms. Even where a treatment was compared with placebo—where we would expect to have the best chance of identifying a difference in SAE rates—there was rarely evidence of a statistically significant difference in SAE rates. This was true for both individual trials and the meta-analyses (Figs. S22 and S23). Where the 95% confidence intervals excluded the null (eg in type 2 diabetes trials with a placebo comparator), the magnitude of such differences was much smaller than the differences in rates we observed between trial participants and individuals in the community.

### Association between multimorbidity count and SAEs in trials (IPD) and routine care

**Fig. 4:**
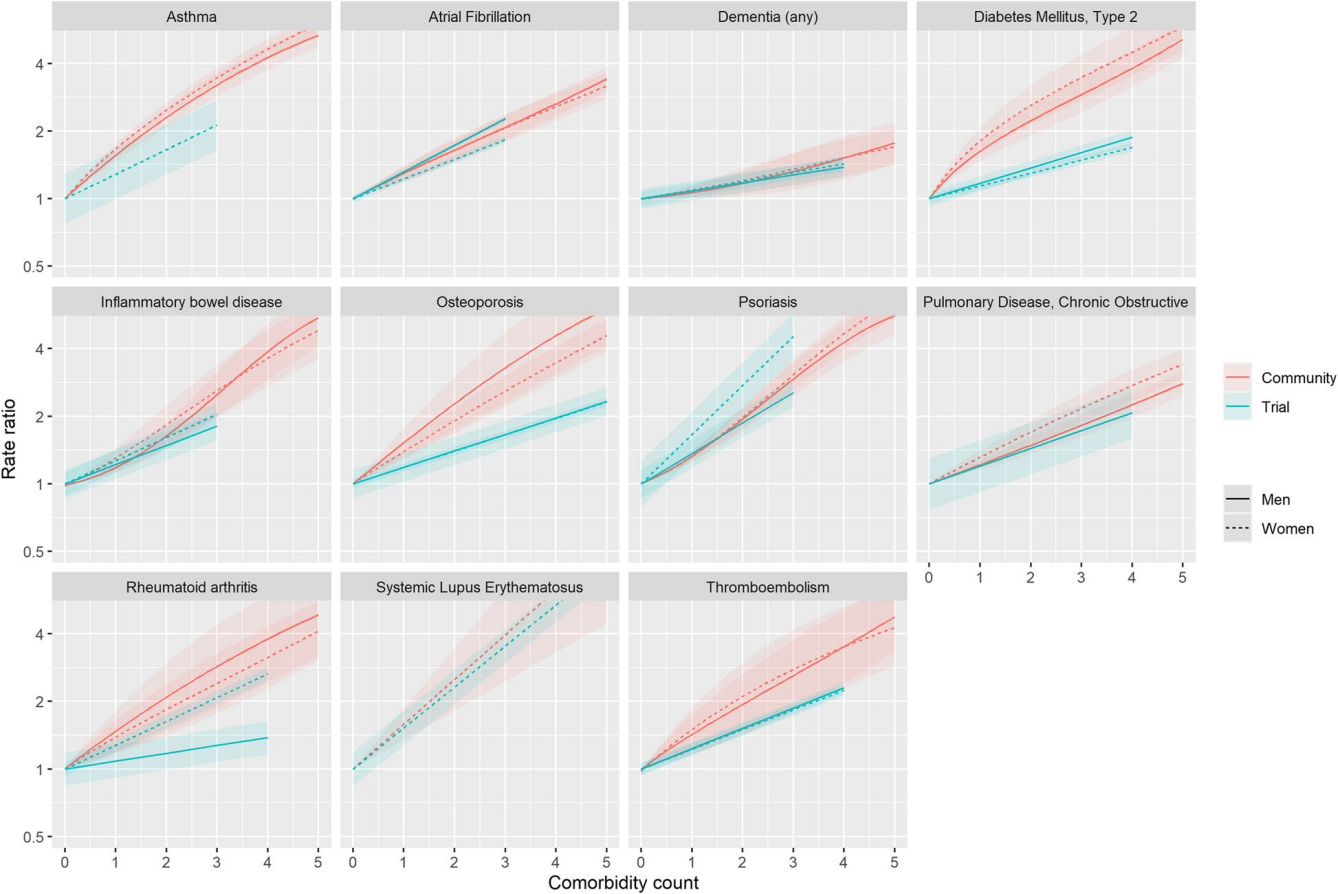
This figure shows the relationship between multimorbidity count and SAE rate in men and women meta-analysed for trials of each index condition (blue) and for each index condition in routine care (red). Shaded areas indicate 95% confidence intervals for the meta-analysis and routine care estimates

For all 21 index conditions, the multimorbidity count predicted the SAE rate in routine care. In the 11 index conditions for which we had sufficient data, multimorbidity count also predicted the SAE rate in the trial data. These associations are shown in Fig. 4 for trials and routine care, respectively. The SAE rate did not differ across trial treatment arms (RR men 0.91; 95%CI 0.81–1.02, RR women 0.99; 95%CI 0.87–1.10), and multimorbidity predicted SAE rates similarly in trial treatment arms and control arms (rate ratio (RR)-interaction 1.02; 95%CI 0.97–1.06).

### Comparison of observed and expected trial SAE rates before and after accounting for multimorbidity

**Fig. 5:**
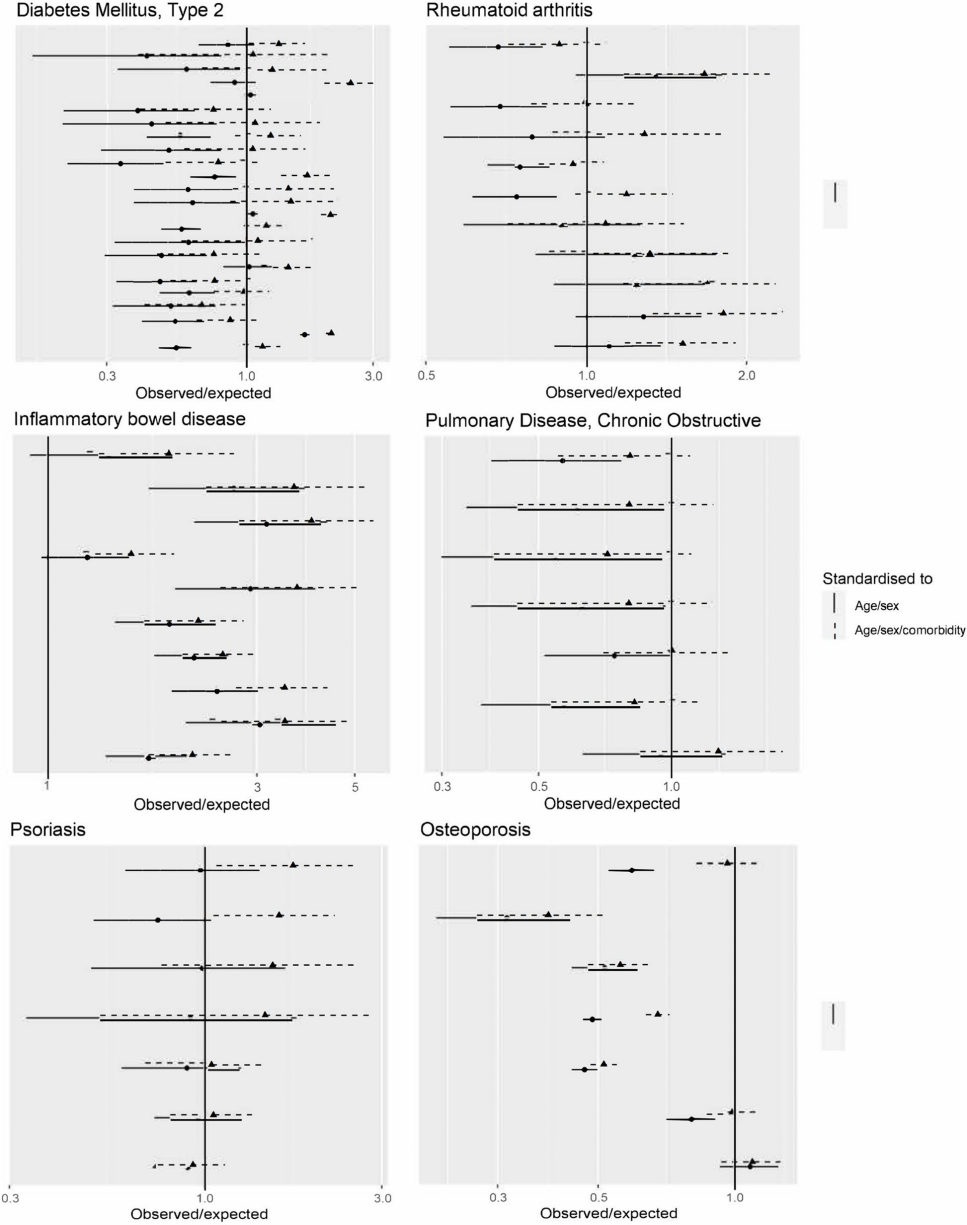
Ratio of observed/expected SAE based on age and sex (square points with solid lines indicating 95% confidence interval), and based on age, sex and multimorbidity count (triangle points with broken lines indicating 95% confidence intervals) for six selected index conditions. Each pair of points correspond to a single trial. Ratios for all other conditions are shown in the supplementary appendix

Since multimorbidity counts are lower in trial than in routine care populations (results reported previously) [7] and multimorbidity counts predict SAE rates in routine care populations (Fig. 4), the ratio of observed/expected SAE is inevitably higher when multimorbidity count is included in the standardisation than when age and sex alone are included. For most trials, for which the age-/sex-adjusted ratio was <1, this meant that additionally adjusting for multimorbidity attenuated the observed/expected ratio closer to one. Figure 5 shows the magnitude of this effect for the four index conditions with the greatest number of IPD trials (other conditions shown in supplementary material). The solid line indicates the ratio of observed to expected based on age and sex and the dotted line the ratio of observed to expected based on age, sex and multimorbidity count. In some cases, the impact of accounting for the multimorbidity count was sufficiently large for the ratios to move from being below one to being at or above one. However, for most trials the observed/expected SAE ratio remained <1 regardless of whether the expected count was also based on multimorbidity (Fig. 5 and supplementary appendix). This implies that differences in the multimorbidity count between trial and routine care populations may account for some, but not all, of the difference in event rates between trials and routine care.

## Discussion

We compared SAEs in trials to the expected number of SAEs based on age-/sex-adjusted hospitalisation and death rates among people with the same index conditions in routine care. For most trials, and the majority of index conditions, trials had fewer than expected SAEs. On meta-analysing 483 trials with aggregate-level data, across 21 index conditions, we found that the point estimate for this ratio was below one for 18 index conditions and that for 12 of the 18 index conditions the confidence intervals did not include one. Secondly, we assessed the relationship between multimorbidity (which is known to be less common in clinical trial populations) and SAEs. Multimorbidity count was associated with increased SAE rates in trials as well as in routine care. Finally, we found that, where the expected SAE count in trials was derived from age, sex and multimorbidity (rather than age and sex alone), the observed/expected SAE ratio for most trials in most index conditions moved closer to one. Despite this, the observed SAEs remained lower than expected in most trials, even after additionally accounting for multimorbidity. These findings show (i) that age/sex standardised observed rates of SAEs are lower than expected in trial populations; (ii) that some of this difference is explained by lower levels of multimorbidity within trial populations; and (iii) that many trials are not representative even after age, sex and multimorbidity standardisation, suggesting that trial populations differ systematically from those treated in routine care in aspects not fully accounted for by a simple multimorbidity count.

Our findings suggest that trial populations on average experience fewer SAEs than people with the same index conditions in routine care. While this suggests trials are often under-representative, there could be several other factors contributing to differences between trials and routine care, and to heterogeneity in the observed/expected ratio between trials. It is possible that trial participation led to better quality care for some participants, which in turn may reduce the likelihood of hospitalisation or death. Furthermore, under-reporting of SAEs by trial sponsors would also result in the differences seen. However, SAE reporting is a regulatory requirement for drug approval and our aggregate data analysis was limited to the time period in which this requirement was in place. While these factors may account for some difference between trials and routine care, for many trials, the difference between trials and routine care was large and unlikely to be explained by these other factors alone. Furthermore, taking type 2 diabetes as an example, many of the trials for which the observed/expected SAE ratio was closer to one were trials in which the population of interest was likely to be at higher risk of SAEs (e.g. people with nephropathy or at increased cardiovascular risk). Our findings are therefore consistent with previous literature suggesting that many clinical trial populations are not representative, and that trial participants are on average healthier than patients encountered in routine care, including having a lower prevalence of multimorbidity. It further suggests that the examination of expected and observed SAEs may offer additional insights into trial representativeness.

### Comparison with other studies

It is widely recognised that many trials exclude a large proportion of people with the condition of interest [1]. Specifically, older people and those with multimorbidity or frailty are often excluded from trials (either explicitly or implicitly) [6, 7, 12]. It has been argued that this lack of representativeness limits the generalisability and applicability of trial findings [13, 14], and more recently this uncertainty has been reflected in clinical guidelines for managing multimorbidity [15]. However, assessment of representativeness is also challenging as reporting of exclusion criteria and participant characteristics is variable and often limited [16].

Two previous studies used psoriasis registry data to compare rates of SAEs in trial ineligible vs trial eligible patients, with lower rates observed in the population eligible for trial participation [17, 18]. Another study found higher rates of SAEs in a UK psoriasis registry than in IPD from two psoriasis trials, even after re-weighting the register data to more closely resemble the trial populations [19]. Our own previous study also compared SAE rates in trial participants and patients in routine care finding higher rates in the routine care population after age-sex standardisation, but did so solely for trials of agents acting on the renin-angiotensin-aldosterone system in order to treat hypertension [9]. The current study adds to this somewhat sparse literature by examining the trial age-sex standardised observed/expected SAE ratio across a wide range of index conditions. For many conditions, particularly those predominantly affecting older people and in which multimorbidity is common, we showed that most trials have substantially lower rates of SAEs than expected, suggesting that most trials are unrepresentative.

Our observation that the multimorbidity count was associated with SAEs and with hospitalisation and death similarly across index conditions adds to the previous literature showing that multimorbidity predicts death and hospitalisations in the general population [20, 21, 22, 23, 24], and that SAEs are associated with the frailty index in trial participants [6]. We are not aware of any previous study exploring the relationship between multimorbidity and SAEs in trials.

### Implications

Our findings suggest that trials systematically select people at lower risk of SAEs. As a result, even if the relative benefit of the trial treatment were the same for all patients, the overall net benefit of treatment may be different for people at higher risk of SAEs who are more likely to be excluded from trials. It is therefore important to assess trial representativeness in order to judge the extent to which trial-derived estimates of relative and absolute treatment effects (e.g. odds ratios and absolute risk reductions, respectively), net overall treatment benefits (balancing the effects of treatments on target and adverse events) and cost-effectiveness can be applied to clinical practice [25]. By design, trials exclude many people with the condition being treated. Even accounting for explicit eligibility criteria, it seems likely that, even in the absence of specific exclusions addressing this, trial investigators may be less likely to recruit patients they suspect of being liable to early withdrawal or to experiencing adverse events due to multimorbidity or frailty. Furthermore, trial descriptions of baseline characteristics rarely capture all relevant characteristics of trial participants; frailty and multimorbidity, for example, are rarely included in such summaries. Currently, approaches to assess trial representativeness are based on detailed consideration of the trial design (recruitment strategies, eligibility criteria, etc.), participant flow (numbers screened, enrolled, etc.) and participant characteristics. There are useful tools to guide this process, but it remains complex, time-consuming and impractical for widespread, rapid use. Furthermore, a detailed assessment of trial participant characteristics relies on these characteristics being reported in trials, which is not always the case for important measures such as socioeconomic status, multimorbidity or frailty.

In this context, assessing the observed/expected SAE ratio may be a useful metric to aid the assessment of trial representativeness. This may be used to supplement more complex methods of assessing representativeness. A possible advantage of the observed/expected SAE rate ratio is that it is a single number which provides an integrated measure of the susceptibility of trial participants to SAEs relative to general populations with the relevant index condition. Moreover, it is based on a measure which is a fundamental component of current clinical trial reporting [26, 27]. At present, however, because there is no benchmark against which to judge the observed SAE count, this potentially useful information on representativeness is opaque. We show that after defining a notional target population to whom trial findings may apply, one can use age and sex-specific hospitalisation and death rates for that population to estimate the observed/expected SAEs ratio for individual trials.

Using the observed/expected SAE ratio to assess the representativeness of a given trial will require careful consideration of both the population identified from routine care and the arm of the trial used to assess the SAE count. In this analysis, having found no difference on average between treatment and control arms, we used the total SAE count from each trial (across all trial arms). However, for some trials, such an approach may lead to biased estimates if the SAE count was higher or lower in the treatment arm. For example, in trials where the treatment itself is likely to influence SAE rates (e.g. of potentially toxic treatments such as chemotherapy), it may be more appropriate to restrict the trial data to the control arm where the control treatment is more comparable to routine care. To facilitate such comparisons, reporting guidelines should encourage trialists to report the age distribution, total follow-up time and SAE counts stratified by study arm and by sex. Where there is a difference in SAEs between treatment and control arms, it is likely that the control arm would provide the most meaningful comparison with routine care (particularly where the control involved active treatment reflecting ‘usual care’). Selection of the routine care population is also important when considering the representativeness of a specific trial. It may be more appropriate to select a routine care comparison to which the trial treatment is likely to be indicated (rather than the broad approach or including all those with the condition of interest). Furthermore, when treatment and control arms both include active treatment (which may influence SAE rates) is may be preferable to compare SAEs with patients receiving comparable treatments in routine care. We did not attempt to make such nuanced judgements for each trial assessed in this analysis, given the broad range of index conditions, treatment indications, and the large number of trials. However, future applications of this approach to assessing representativeness will need to judge the appropriate routine care population and trial arm comparison in the context of the trial(s) being assessed.

Although, for individual trials, combining SAE counts across arm will increase the precision of the observed/expected SAEs ratios, particular caution should be employed (i) where the magnitude of difference between the community and trial participants is small (and hence small differences between the arms could have important implications for interpretation), (ii) where there is empirical evidence of differences in rates of SAEs between arms, or (iii) where is reason to believe from external evidence (e.g. biological plausibility or findings from other studies) suggesting that treatment arm is likely to have an effect on SAE rates.

In the hope that other groups will adopt our approach, we have provided analysis code, data and a detailed description in the supplementary appendix section.

### Strengths and limitations of this study

Strengths of this analysis include the inclusion of many trials and multiple index conditions and the use of a UK representative routine care population in which expected SAE rates were calculated. However, this broad approach meant that we could not incorporate all characteristics which could affect the risk SAEs (such as socioeconomic status, ethnicity, or severity of the index condition). Had the routine care population been closer to the intended target population for each trial (e.g. by excluding patients with absolute contra-indications for the drug under study, or selecting only those suitable for second-line therapy where this was the trial indication), it is possible that the heterogeneity in the observed/expected ratio would have been lower.

We used the SAE count for all trial participants, not solely those in the usual care arm, in order to increase the statistical precision with which the observed/expected SAE ratio could be estimated. This means that investigational products not yet widely used in routine care may have increased the SAE rate within trials. However, we found that SAEs did not differ by treatment arm and that there was no multimorbidity count-treatment interaction. This suggests that, at least for this set of trials we studied, underlying participant characteristics rather than investigational product-related effects were the major driver of SAEs. However, this may not be true of all trials (e.g. those of potentially toxic treatments such as chemotherapy or immunosuppressive treatments, in which a greater proportion of SAEs in the treatment arm are likely to be directly linked to treatment).

The use of individual participant data allowed us to examine associations between multimorbidity and SAEs within trials, and to explore the extent to which the age-sex standardised observed/expected SAE ratio was impacted by accounting for differences in multimorbidity count by trial and target populations. However, as we reported previously [6, 7], our measures of multimorbidity were based on re-analyses of trial data originally collected for purposes other than measuring multimorbidity. As such, the impact of additionally accounting for multimorbidity may have been larger if better measures were available. Furthermore, this analysis was limited to trials with IPD (which, while comparable in terms of size, start year and exclusion criteria, contain fewer phase 4 trials than the wider body of eligible trials) and with a sufficient number of SAEs per trial to allow estimates of associations (meaning that this relationship could not be assessed for some index conditions). A further limitation is that other events than hospitalisation and death, such as prolongation of hospitalisation, also qualify as SAEs. While the contribution of such events was low (between 0% and 13%), this over-counting would nonetheless tend to bias observed/expected SAE ratios upwards, in most cases giving the impression that trial populations were more similar to routine care populations. Finally, the community population was from Wales, UK, whereas the trials were multinational. Some of the differences between trial and routine care rates may therefore reflect differences in population characteristics or health systems. Differences between trials within a given index condition may also reflect differences in their respective healthcare settings. However, the observed/expected ratio did not appear to differ depending on whether trials did or did not have a site in the UK (supplementary appendix) and rates of hospitalisations and deaths in the UK are comparable to other high-income countries where most of the trials were conducted.

## Conclusions

SAE rates in trials are consistently lower than expected. Multimorbidity is associated with SAEs in both trials and routine care and is less prevalent within trial populations. However, the lower prevalence of multimorbidity in trials only partially explains the difference in SAE rates between trials and routine care, suggesting additional systematic differences between trial and routine care populations. Conventional approaches to assessing trial representativeness are complex, time-consuming, and partial. Our findings suggest that the observed/expected SAE ratio has the potential to be a useful metric of trial representativeness to aid in interpreting the applicability of trials.

## Supplementary Information

**Additional file 1: Supplementary figures S1-S21.** Trial level estimates of observed/expected SAEs. **Figure S1.** Observed/expected SAEs in asthma trials. **Figure S2.** Observed/expected SAEs in atrial fibrillation trials. **Figure S3.** Observed/expected SAEs in axial spondyloarthritis trials. **Figure S4.** Observed/expected SAEs in benign prostatic hyperplasia trials. **Figure S5.** Observed/expected SAEs in dementia trials. **Figure S6.** Observed/expected SAEs in type 2 diabetes mellitus trials. **Figure S7.** Observed/expected SAEs in epilepsy trials. **Figure S8.** Observed/expected SAEs in hypertension trials. **Figure S9.** Observed/expected SAEs in pulmonary hypertension trials. **Figure S10.** Observed/expected SAEs in inflammatory bowel disease trials. **Figure S11.** Observed/expected SAEs in myocardial infarction trials. **Figure S12.** Observed/expected SAEs in osteoarthritis trials. **Figure S13.** Observed/expected SAEs in osteoporosis trials. **Figure S14.** Observed/expected SAEs in Parkinson’s disease trials. **Figure S15.** Observed/expected SAEs in psoriasis trials. **Figure S16.** Observed/expected SAEs in psoriatic arthropathy trials. **Figure S17.** Observed/expected SAEs in chronic obstructive pulmonary disease trials. **Figure S18.** Observed/expected SAEs in pulmonary fibrosis trials. **Figure S19.** Observed/expected SAEs in rheumatoid arthritis trials. **Figure S20.** Observed/expected SAEs in systemic lupus erythematosus trials. **Figure S21.** Observed/expected SAEs in thromboembolism trials.

**Additional file 2: Supplementary figures S22-23** Comparison of SAEs between trial arms. **Figure S22.** Trial-level comparison of SAE rate between trial arms. **Figure S23.** Index condition-level meta-analyses of comparison of SAE rate between trial arms.

**Additional file 3: Supplementary figures S24-S44**. Observed/expected SAEs before and after standardization by multimorbidity count. **Figure S24.** Observed/expected SAEs before and after standardization by multimorbidity count: asthma trials. **Figure S25.** Observed/expected SAEs before and after standardization by multimorbidity count: atrial fibrillation trials. **Figure S26.** observed/expected SAEs before and after standardization by multimorbidity count: axial spondyloarthritis trials. **Figure S27.** Observed/expected SAEs before and after standardization by multimorbidity count: benign prostatic hyperplasia trials. **Figure S28.** Observed/expected SAEs before and after standardization by multimorbidity count: dementia trials. **Figure S29.** observed/expected SAEs before and after standardization by multimorbidity count: type 2 diabetes mellitus trials. **Figure S30.** Observed/expected SAEs before and after standardization by multimorbidity count: hypertension trials. **Figure S31.** Observed/expected SAEs before and after standardization by multimorbidity count: pulmonary hypertension trials. **Figure S32.** Observed/expected SAEs before and after standardization by multimorbidity count: inflammatory bowel disease trials. **Figure S33.** Observed/expected SAEs before and after standardization by multimorbidity count: migraine trials. **Figure S34.** Observed/expected SAEs before and after standardization by multimorbidity count: osteoarthritis trials. **Figure S35.** Observed/expected SAEs before and after standardization by multimorbidity count: osteoporosis trials. **Figure S36.** Observed/expected SAEs before and after standardization by multimorbidity count: Parkinson’s disease trials. **Figure S37.** Observed/expected SAEs before and after standardization by multimorbidity count: psoriasis trials. **Figure S38.** Observed/expected SAEs before and after standardization by multimorbidity count: psoriatic arthropathy trials. **Figure S39.** Observed/expected SAEs before and after standardization by multimorbidity count: chronic obstructive pulmonary disease trials. **Figure S40.** Observed/expected SAEs before and after standardization by multimorbidity count: pulmonary fibrosis trials. **Figure S41.** Observed/expected SAEs before and after standardization by multimorbidity count: restless legs syndrome trials. **Figure S42.** Observed/expected SAEs before and after standardization by multimorbidity count: rheumatoid arthritis trials. **Figure S43.** Observed/expected SAEs before and after standardization by multimorbidity count: systemic lupus erythematosus trials. **Figure S44.** Observed/expected SAEs before and after standardization by multimorbidity count: thromboembolism trials.

**Additional file 4.** Statistical methods.

## Data Availability

Aggregated data and code required to run these models, along with full model descriptions, are available at https://github.com/dmcalli2/sae_ctg_multicond_public

## Abbreviations

CI: Credible interval
CSDR: Clinical Study Data Request
IPD: Individual participant data
SAE: Serious adverse event
SAIL: Secure Anonymised Information Linkage
YODA: Yale Open Data Access
