# Supplementary material for: Assessing trial representativeness using serious adverse events: an observational analysis using aggregate and individual-level data from clinical trials and routine healthcare data
Source: BMC Med. 2022 Oct 28;20:410. doi: 10.1186/s12916-022-02594-9 (PMC9615407; doi:10.1186/s12916-022-02594-9)

Additional file 1:
Supplementary figures S1-S21
Trial level estimates of observed/expected SAEs

Figure S1: Observed/expected SAEs in asthma trials

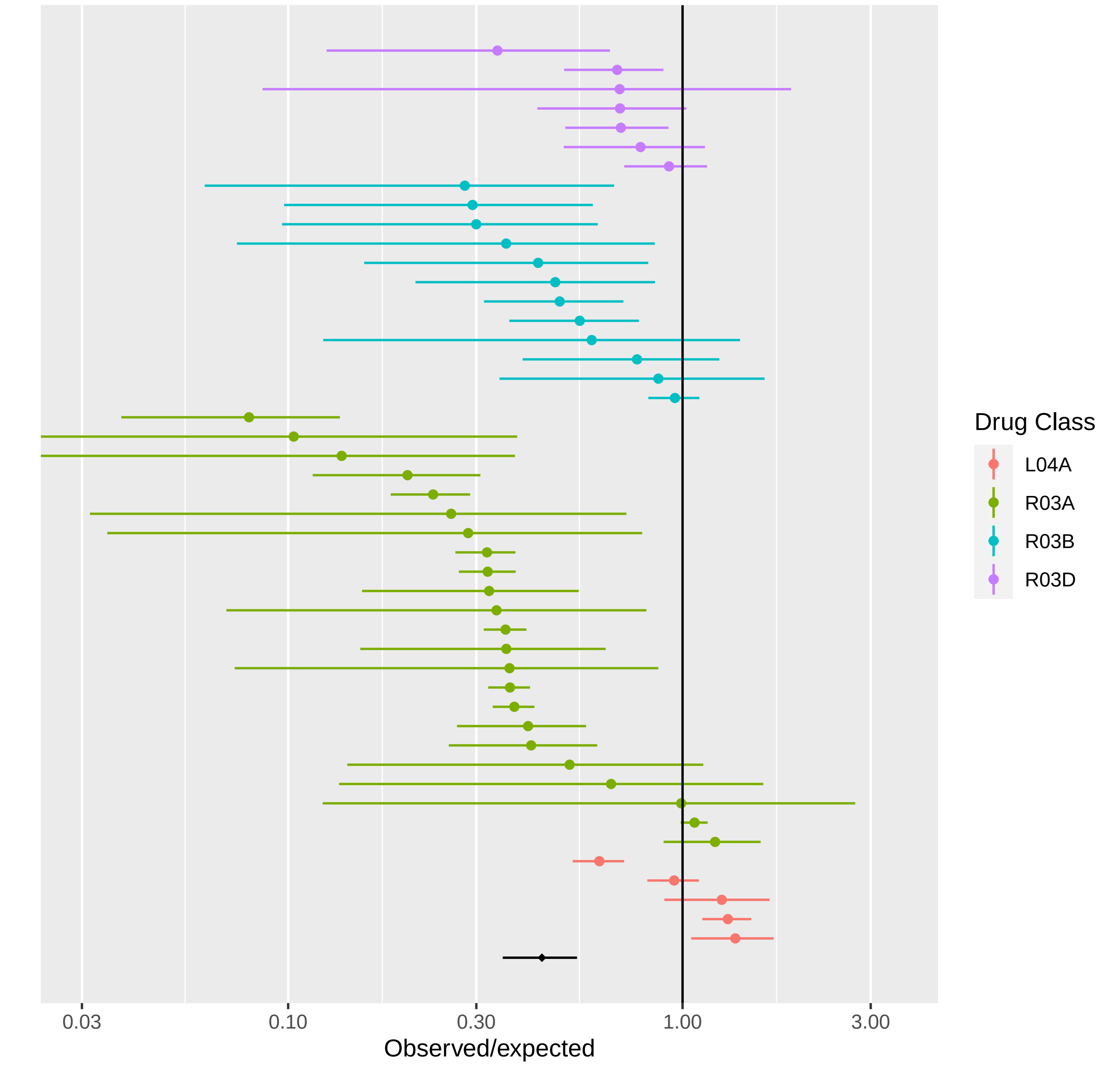

Figure S2: Observed/expected SAEs in atrial fibrillation trials

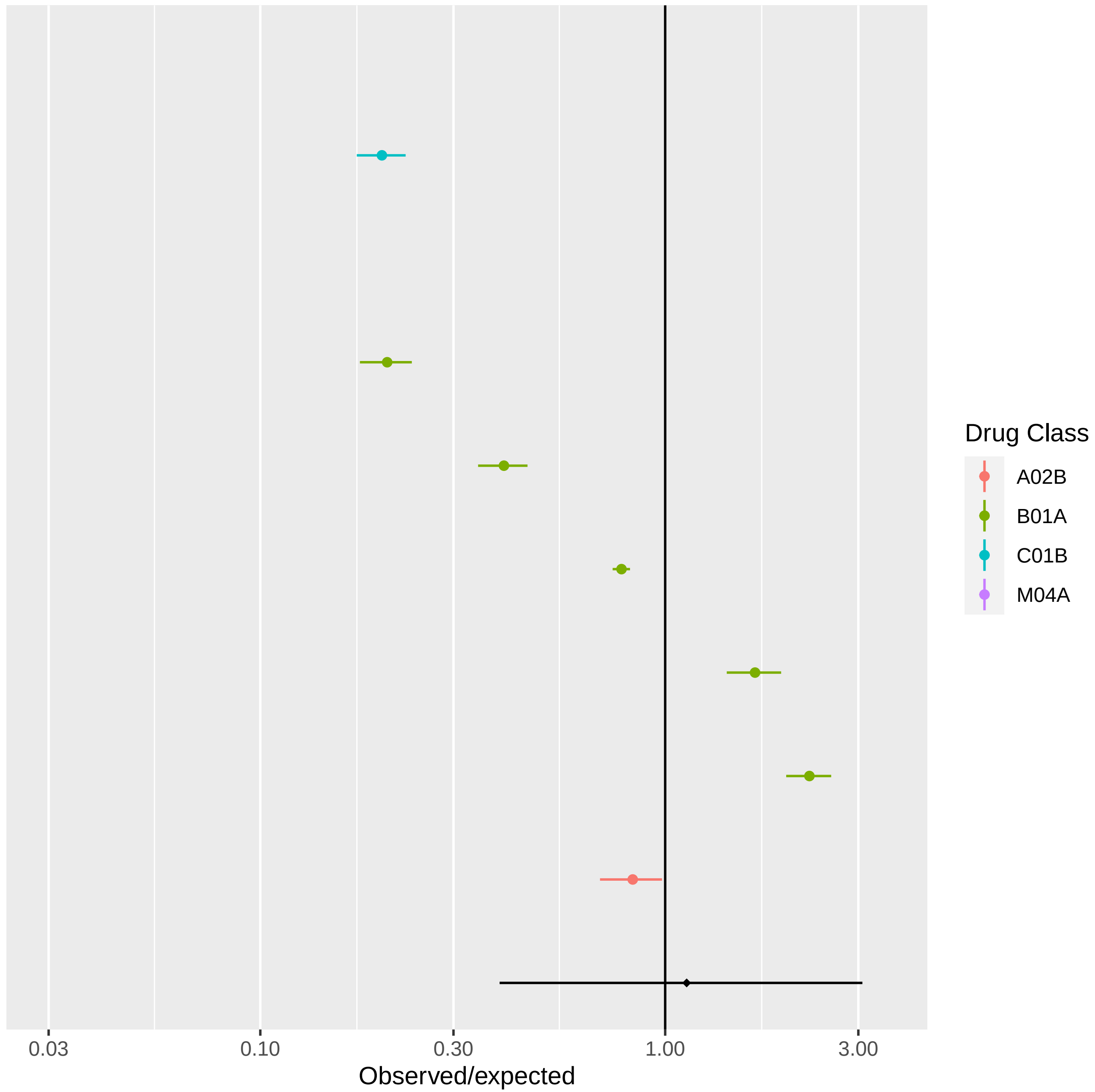

Figure S3: Observed/expected SAEs in axial spondyloarthritis trials

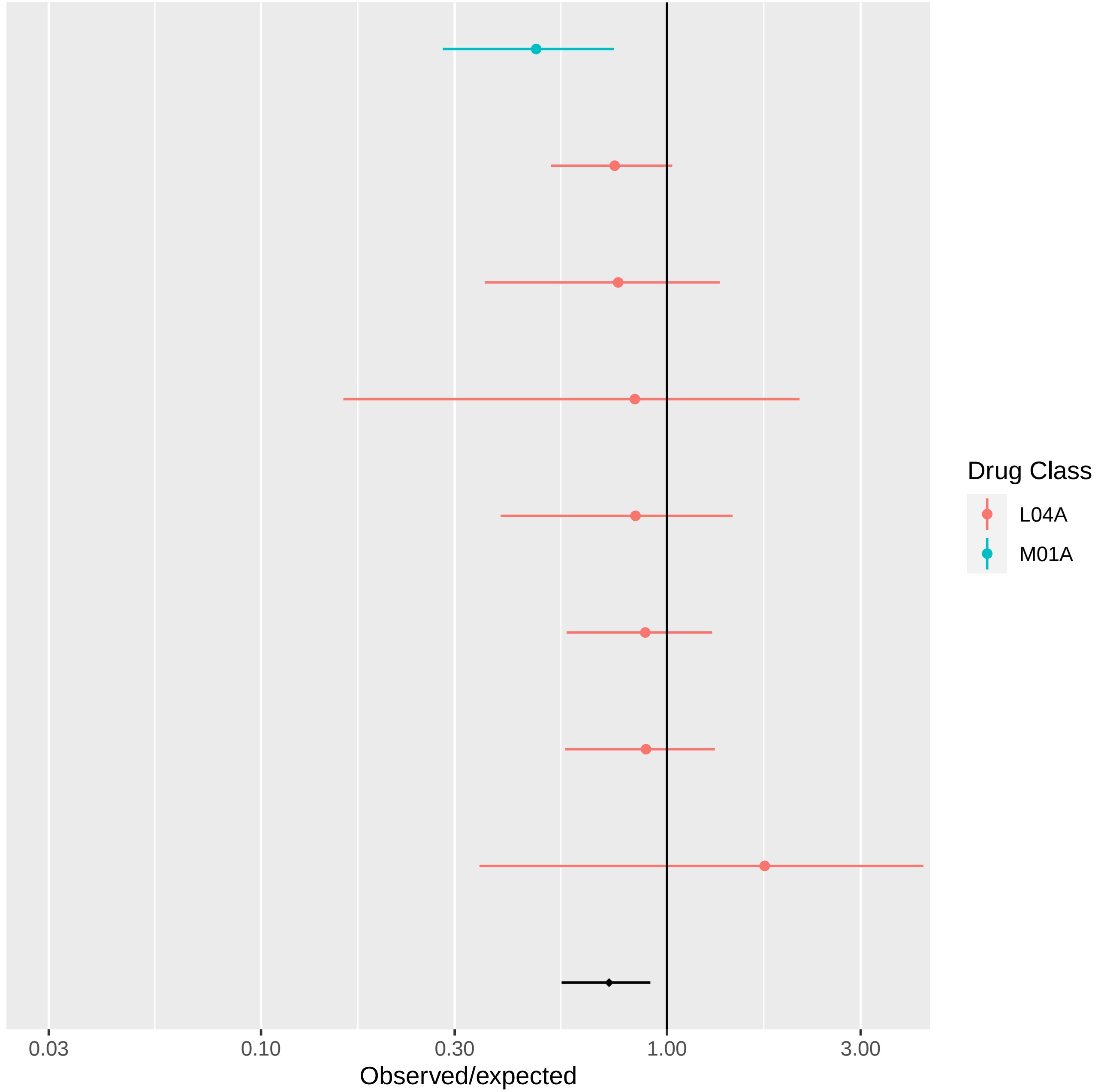

Figure S4: Observed/expected SAEs in benign prostatic hyperplasia trials

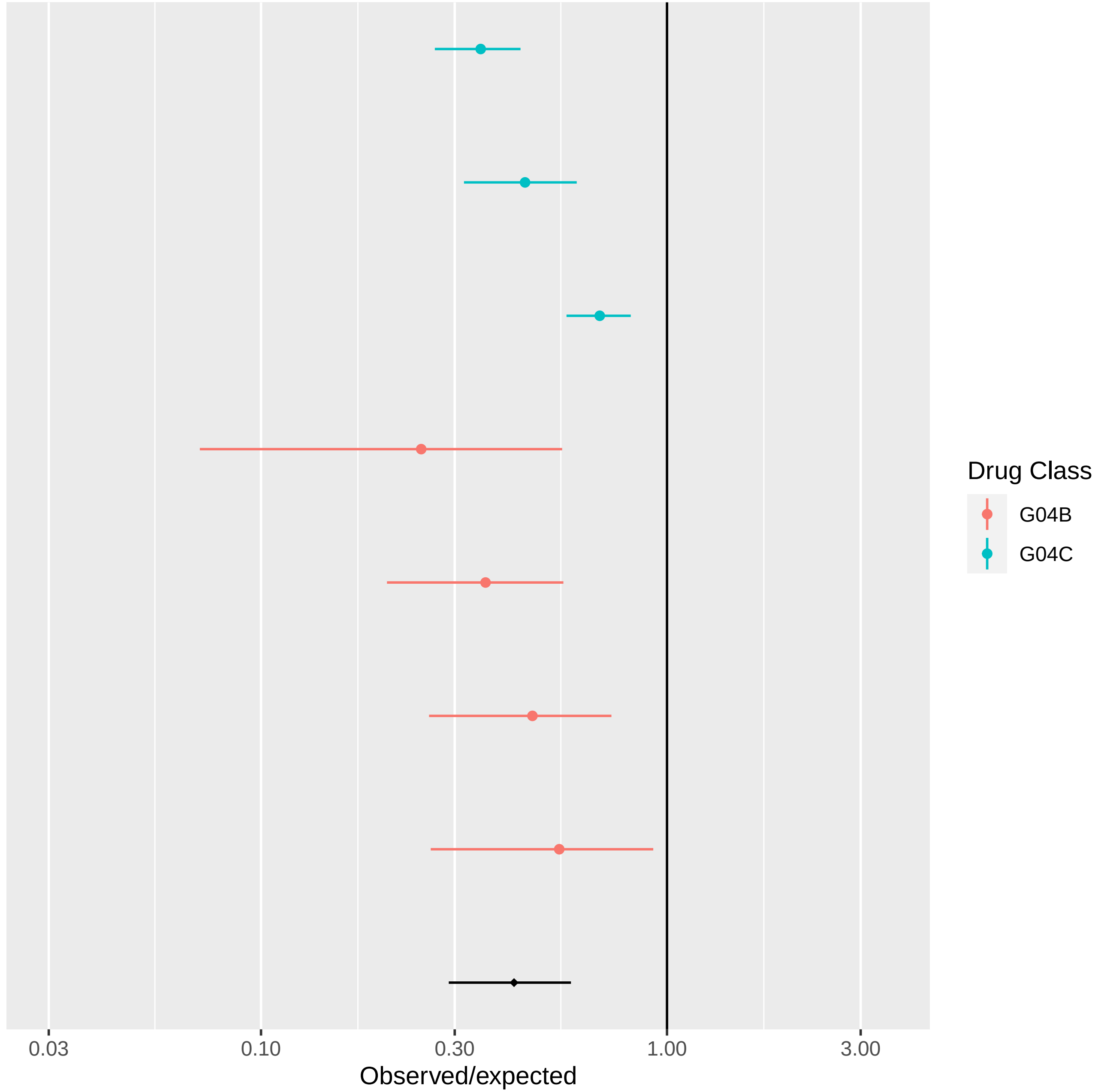

Figure S5: Observed/expected SAEs in dementia trials

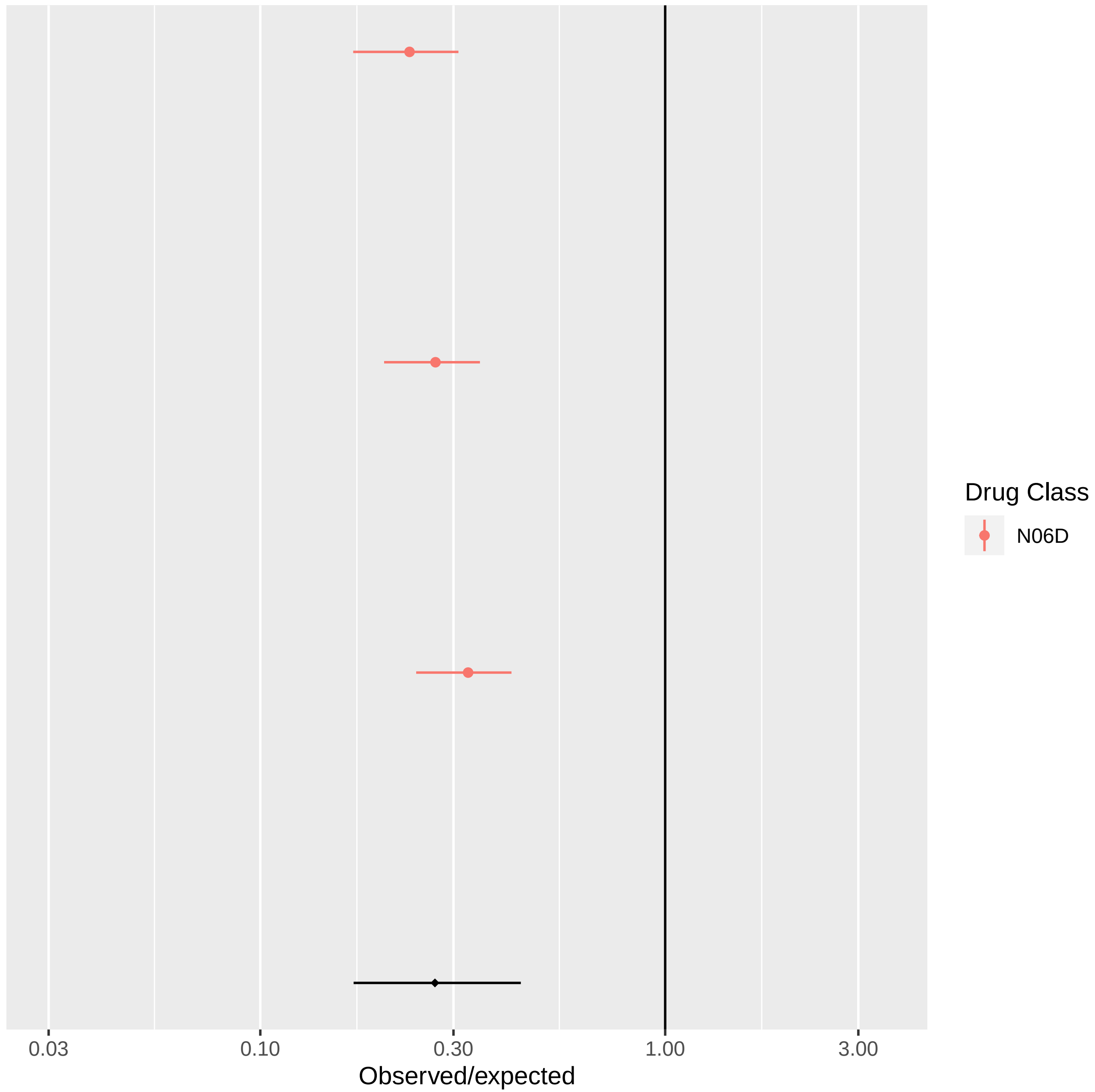

Figure S6: Observed/expected SAEs in type 2 diabetes mellitus trials

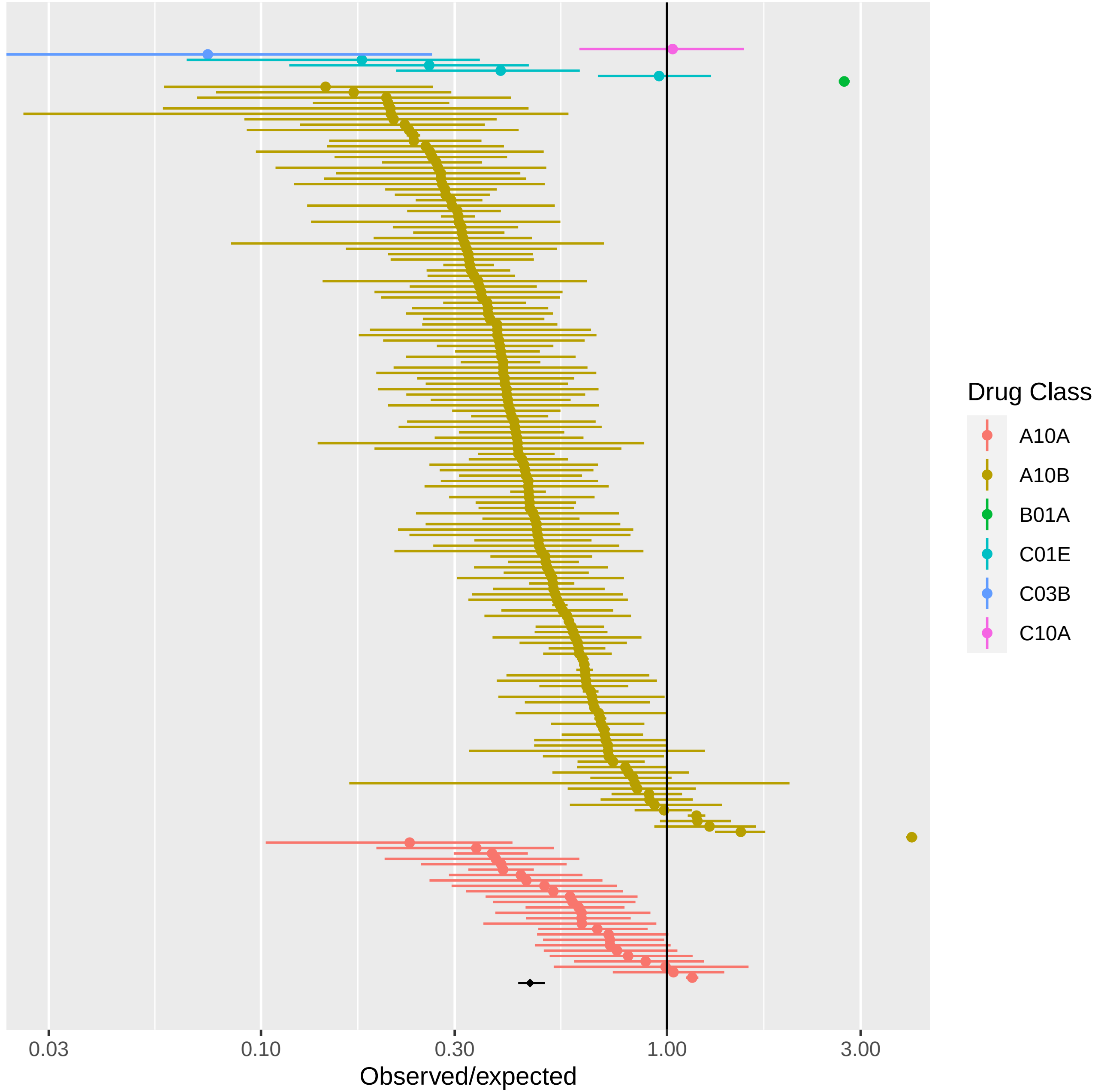

Figure S7: Observed/expected SAEs in epilepsy trials

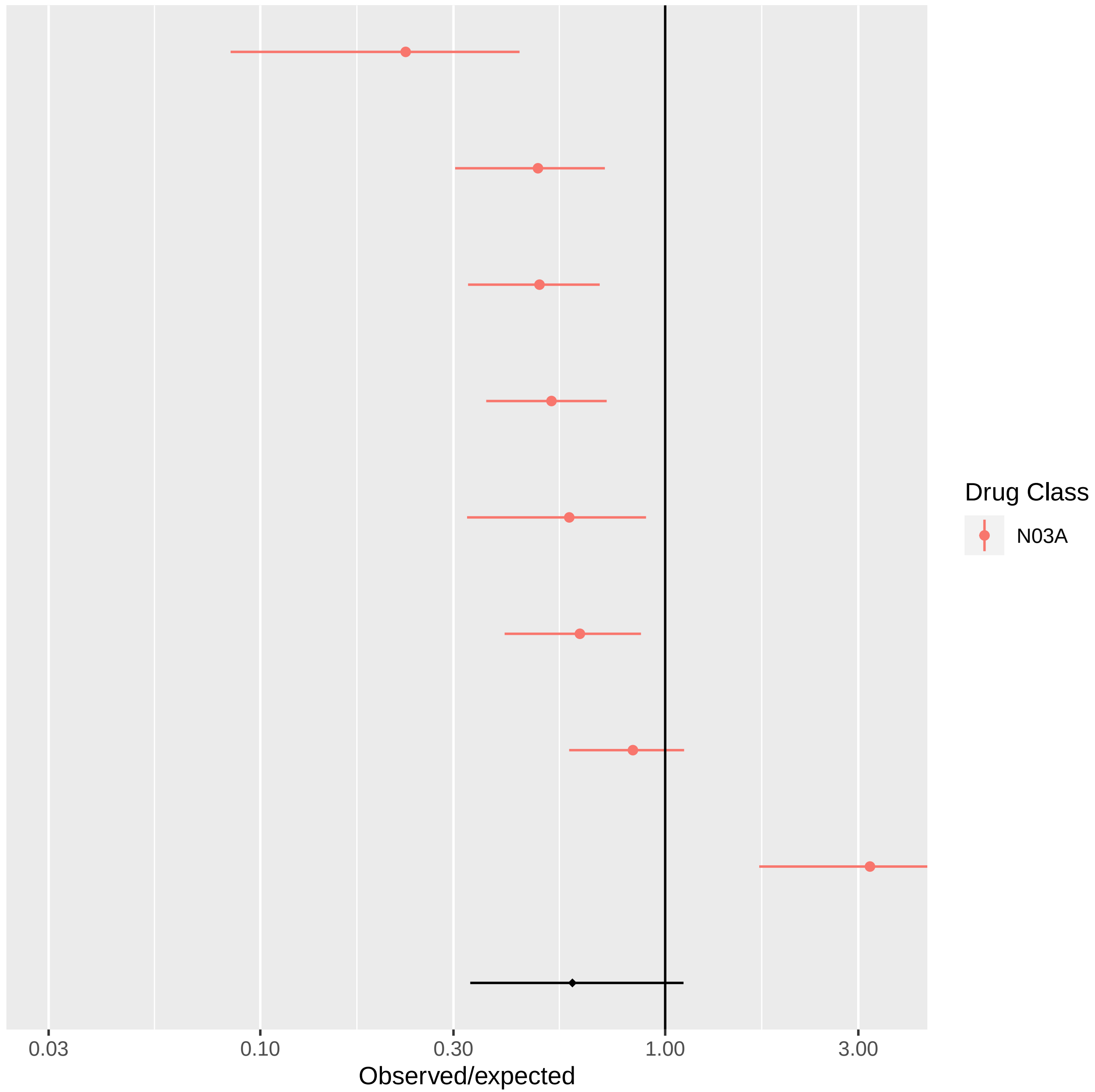

Figure S8: Observed/expected SAEs in hypertension trials

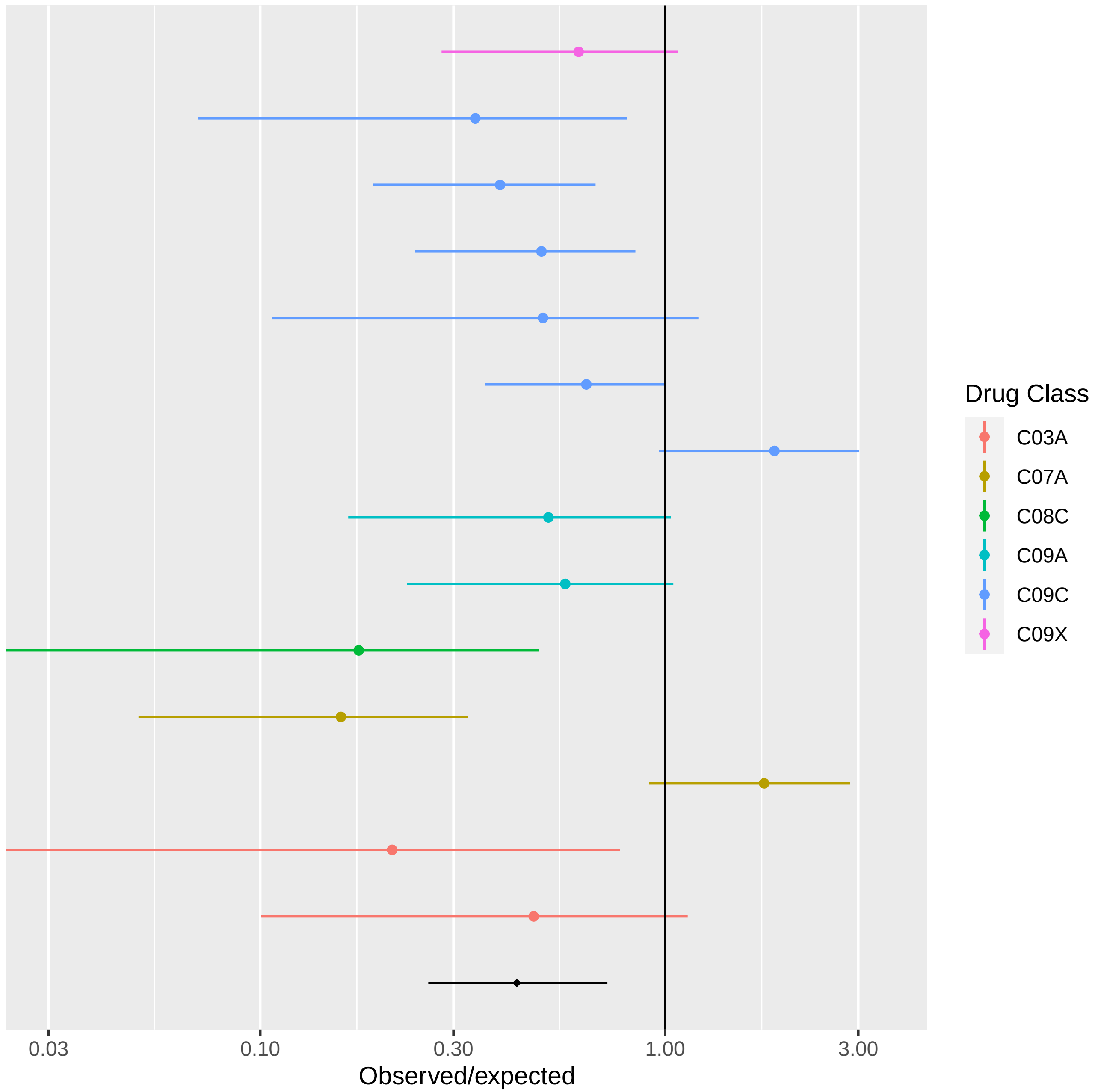

Figure S9: Observed/expected SAEs in pulmonary hypertension trials

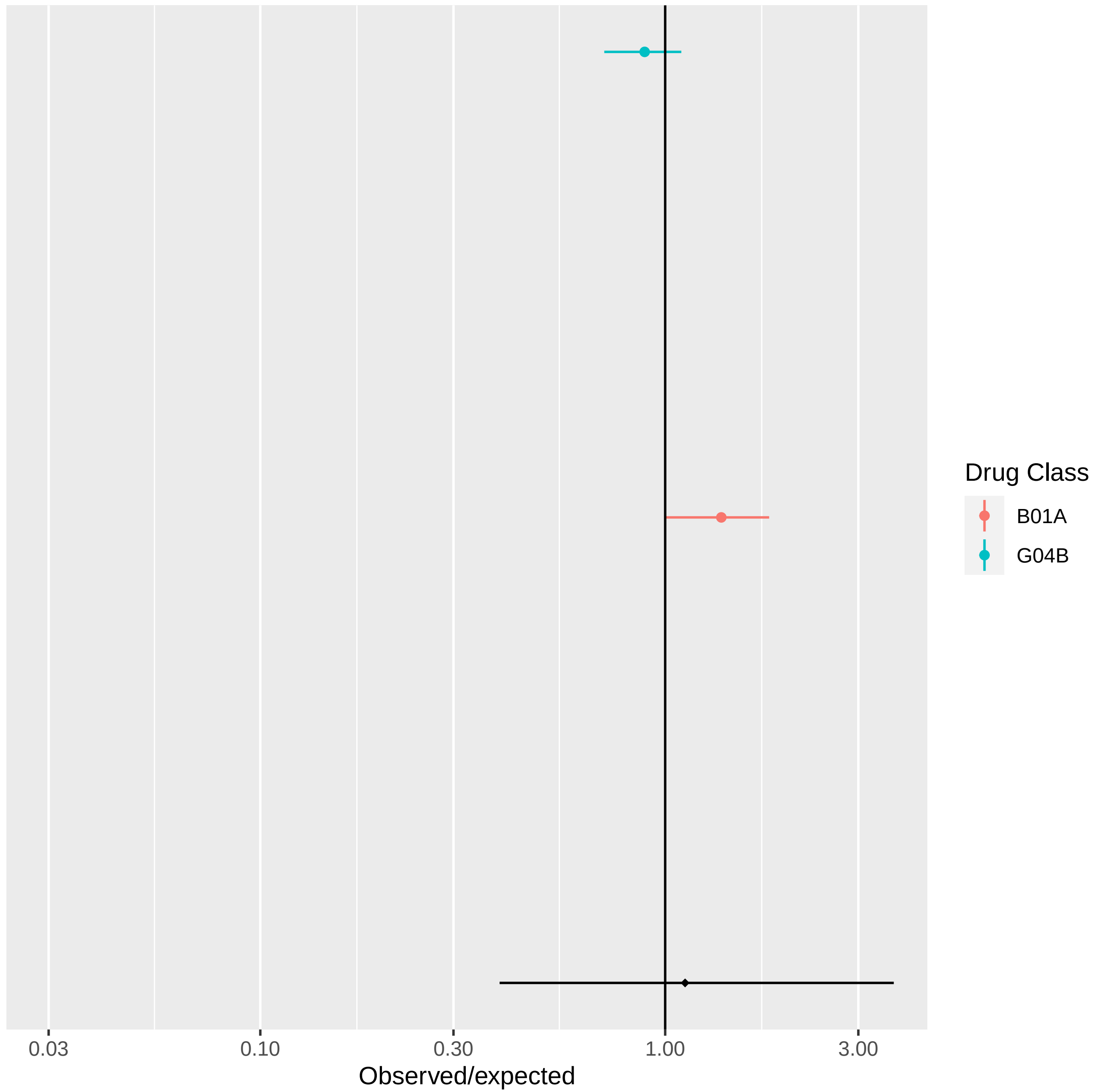

Figure S10: Observed/expected SAEs in inflammatory bowel disease trials

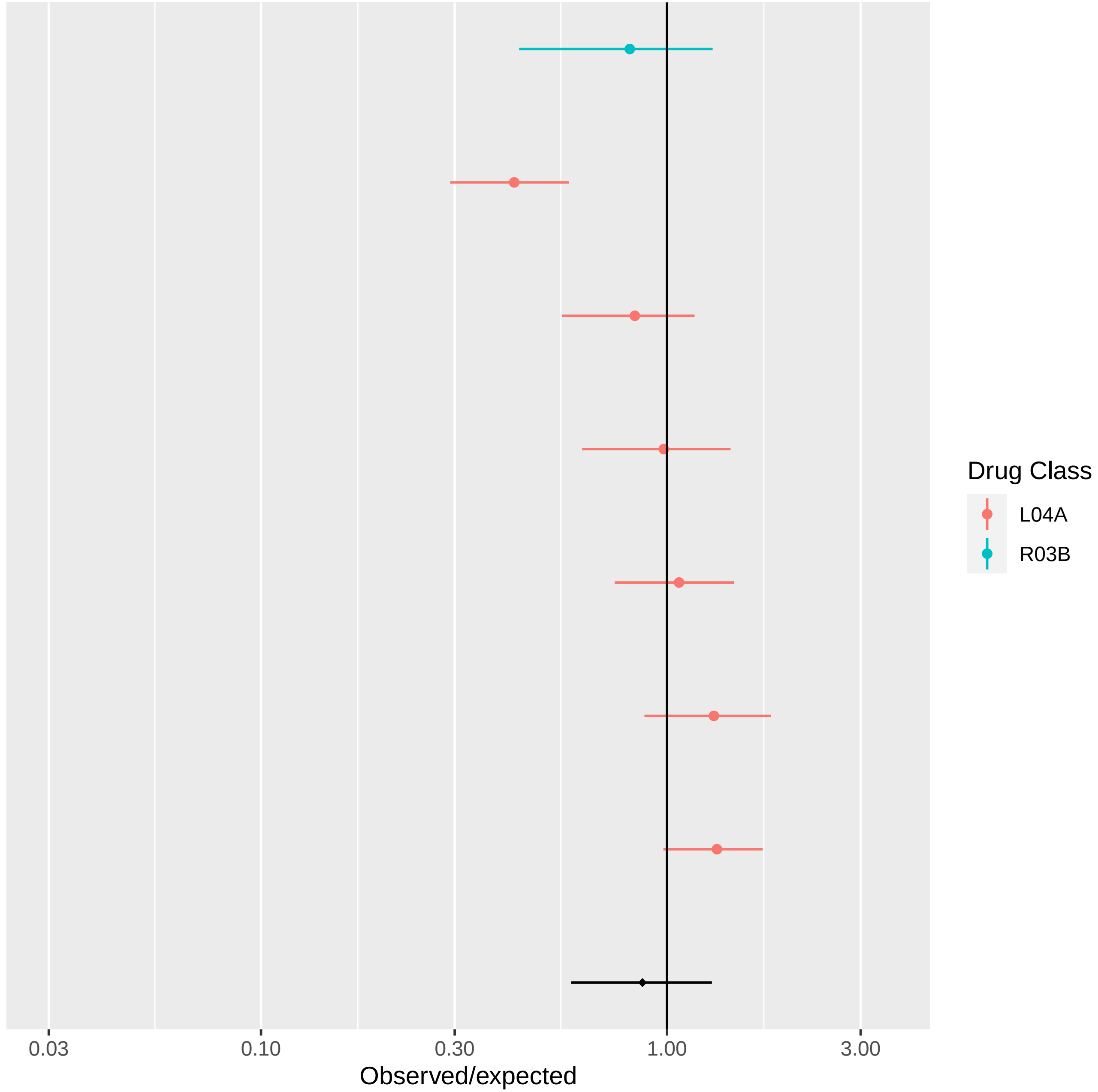

Figure S11: Observed/expected SAEs in myocardial infarction trials

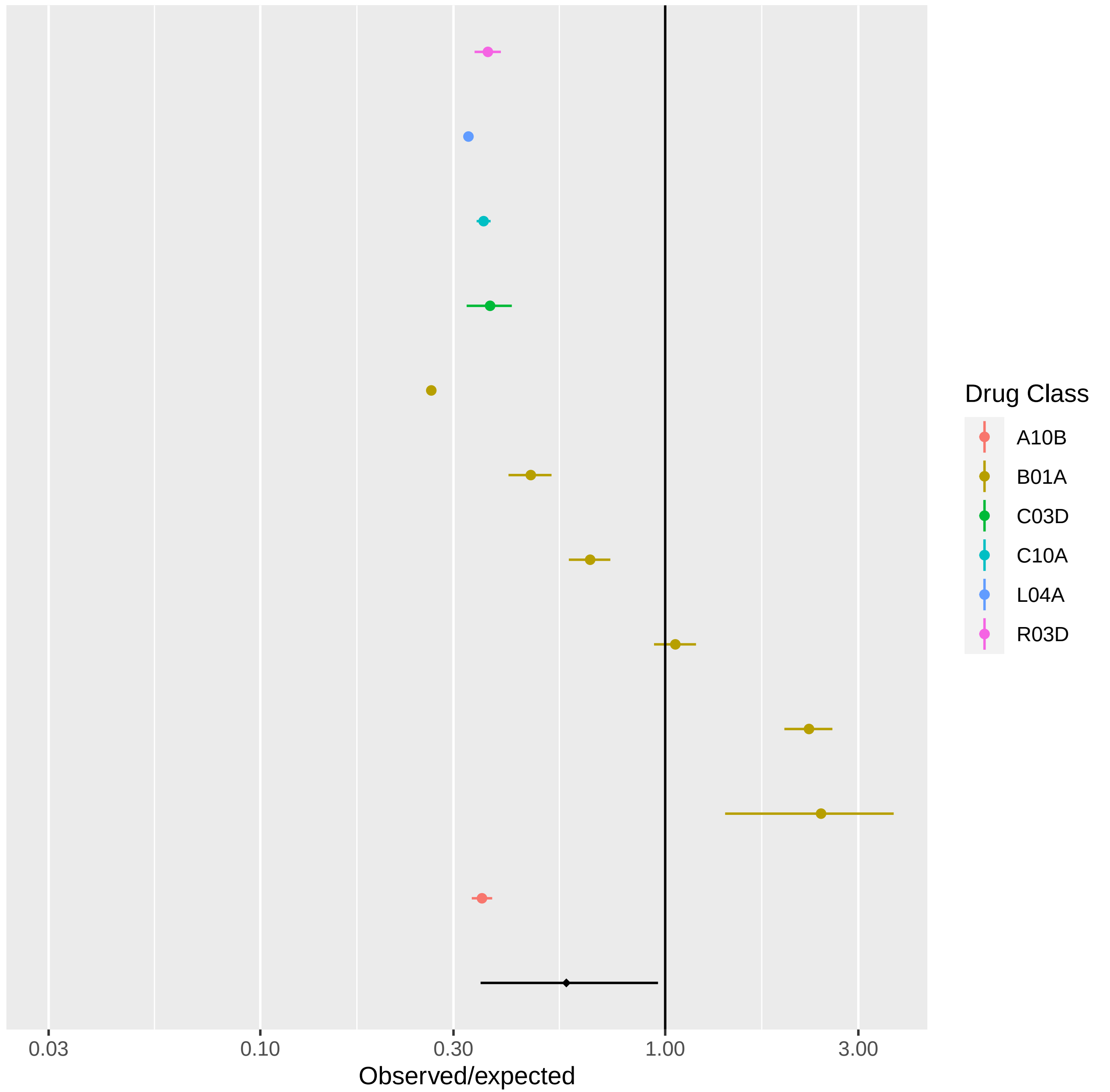

Figure S12: Observed/expected SAEs in osteoarthritis trials

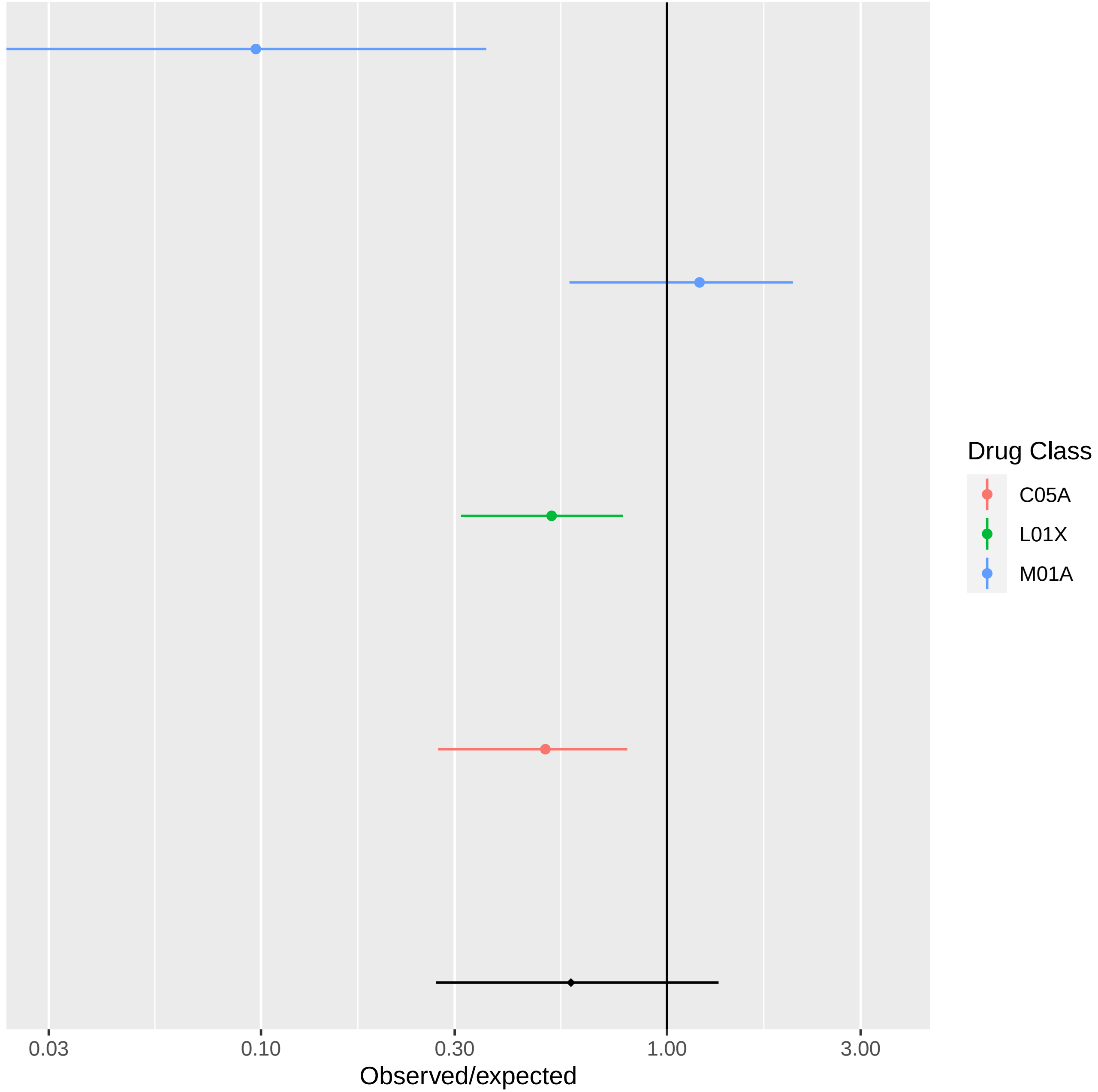

Figure S13: Observed/expected SAEs in osteoporosis trials

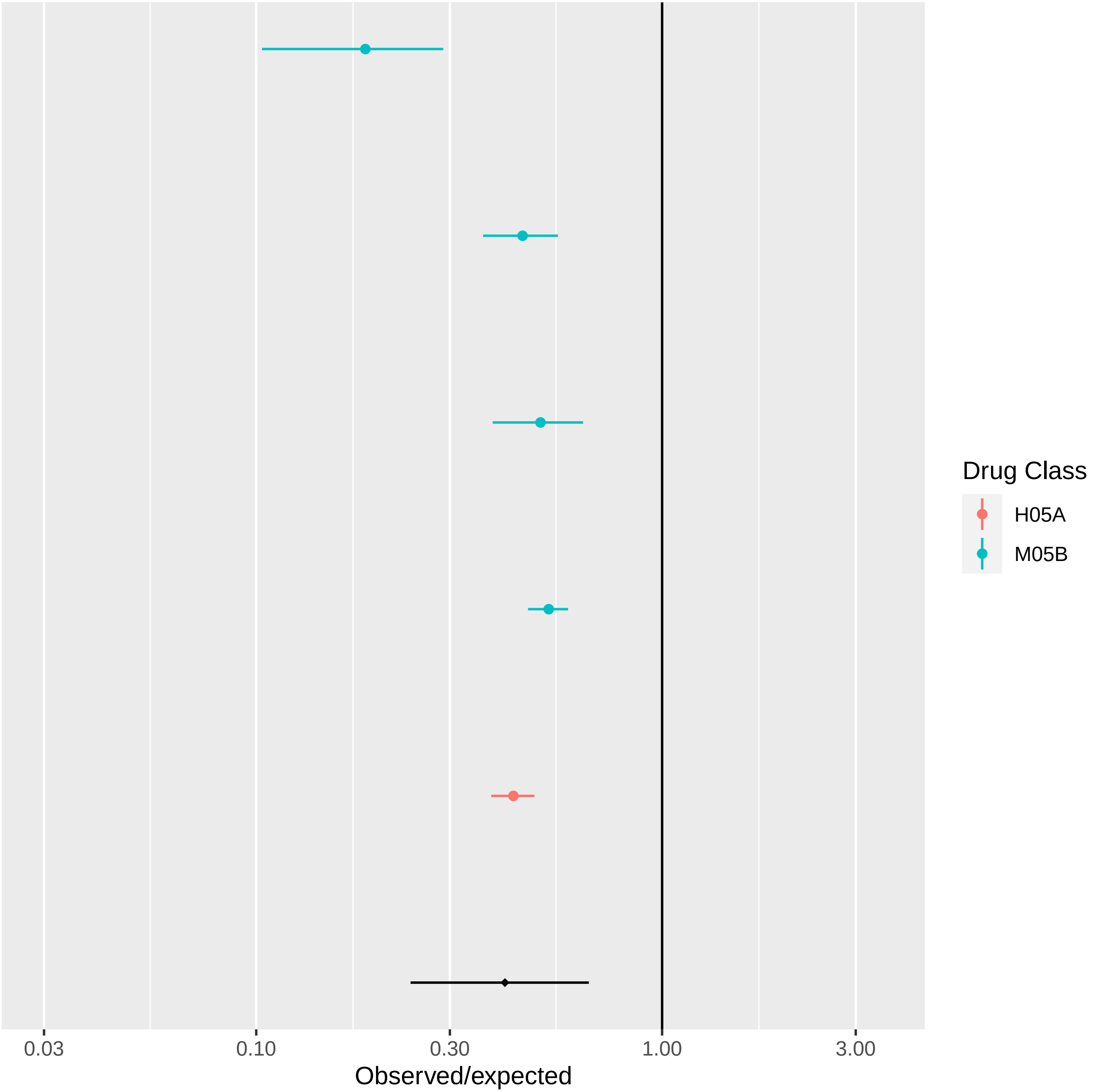

Figure S14: Observed/expected SAEs in Parkinson's disease trials

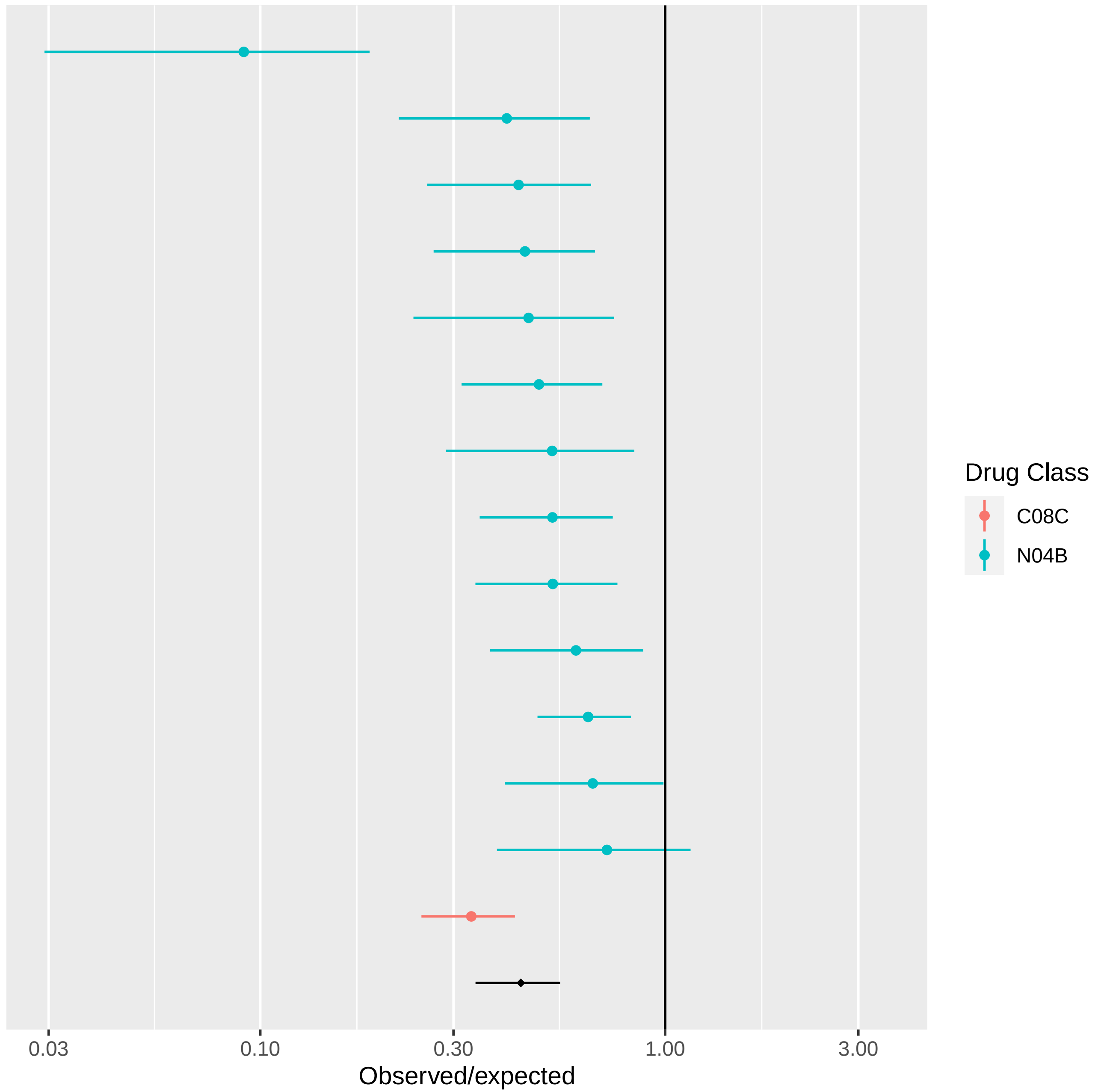

Figure S15: Observed/expected SAEs in psoriasis trials

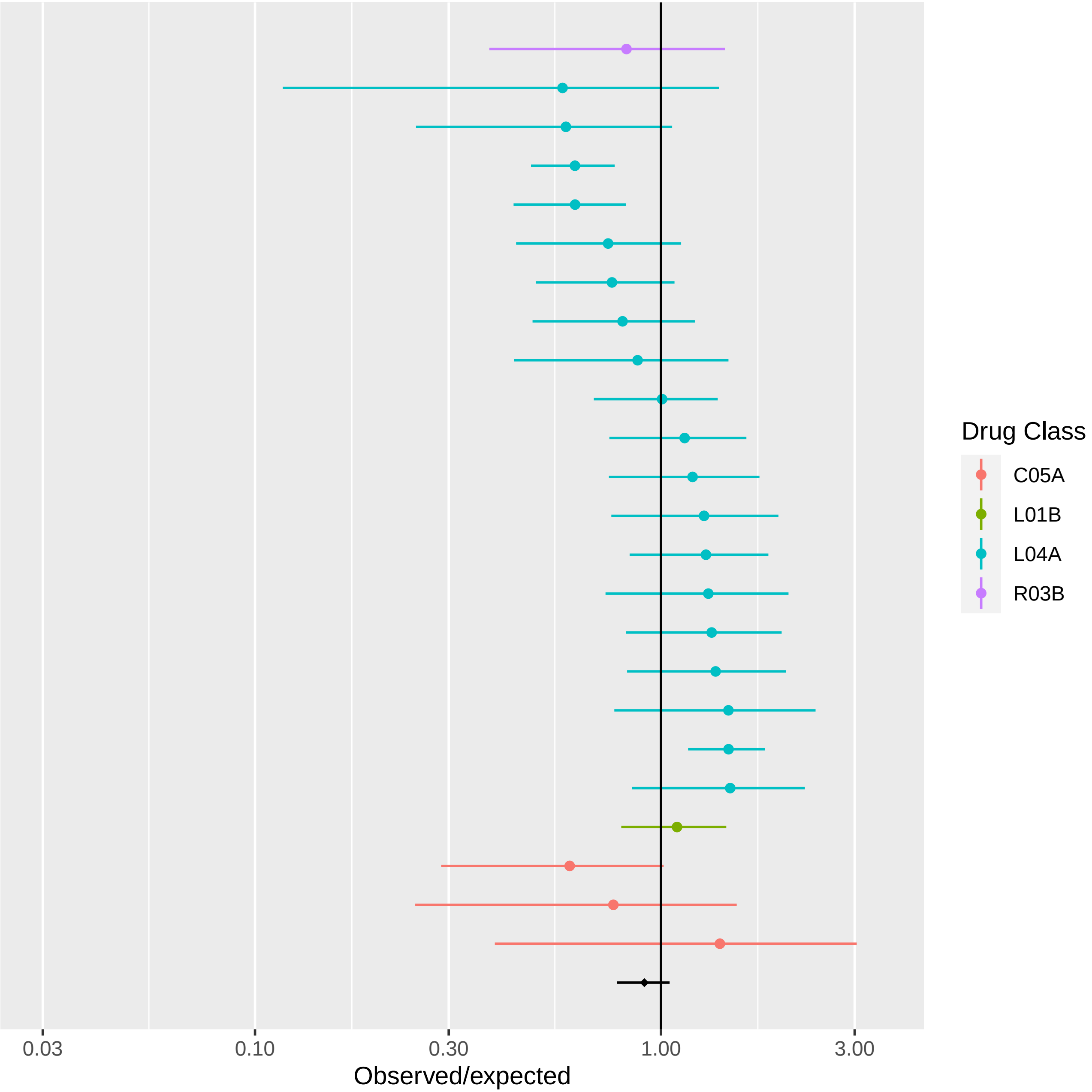

Figure S16: Observed/expected SAEs in psoriatic arthropathy trials

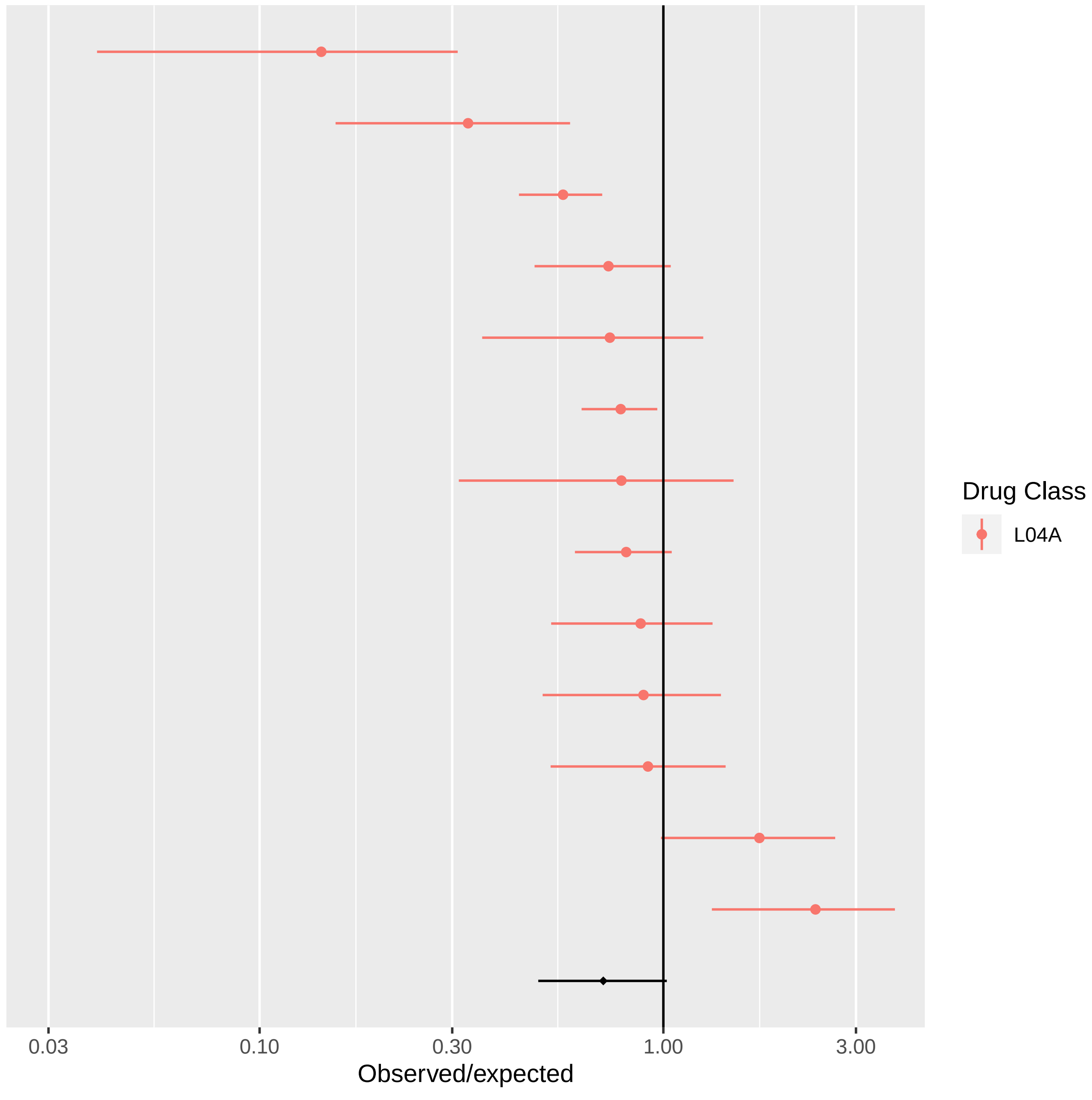

Figure S17: Observed/expected SAEs in chronic obstructive pulmonary disease trials

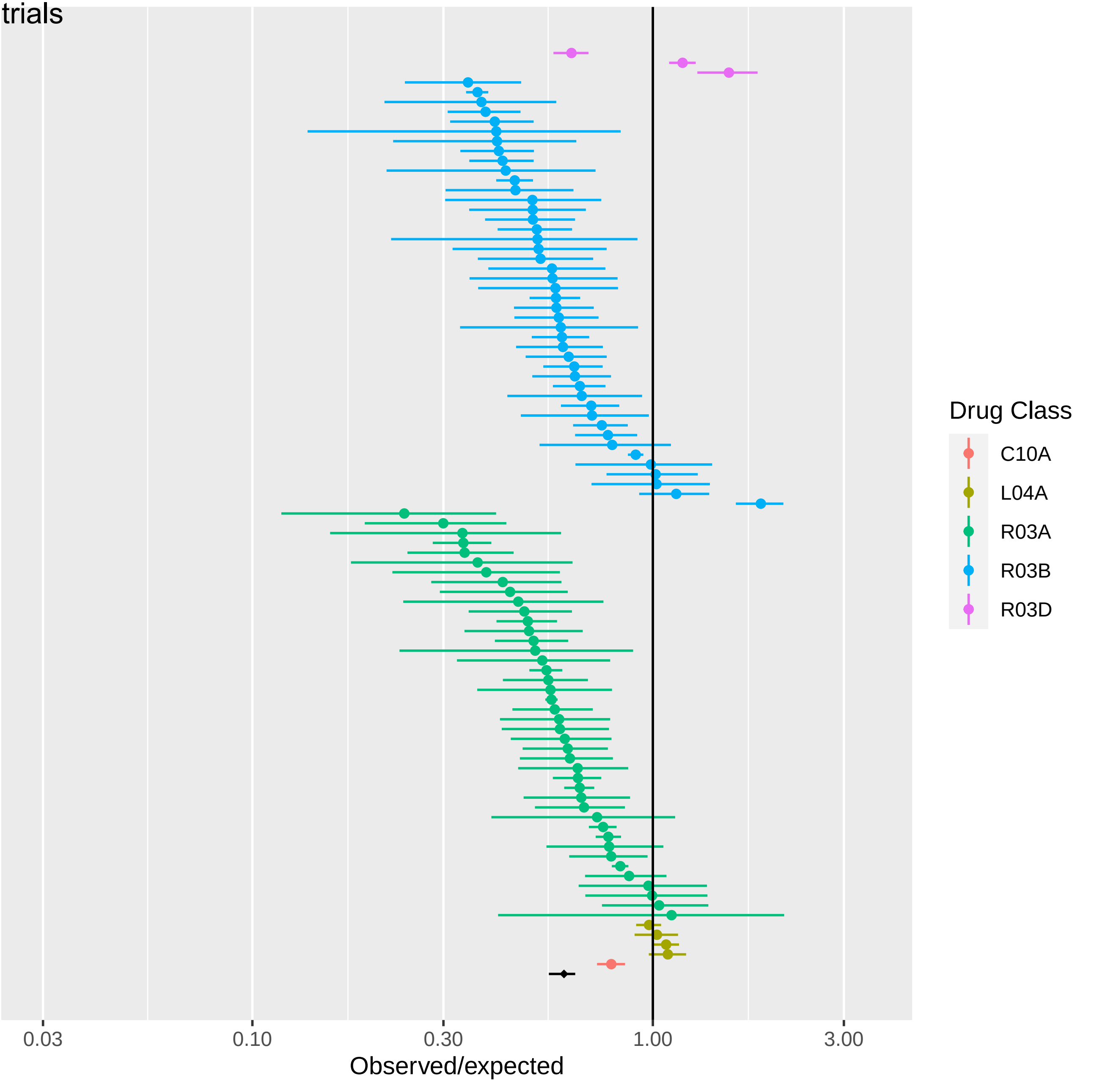

Figure S18: Observed/expected SAEs in pulmonary fibrosis trials

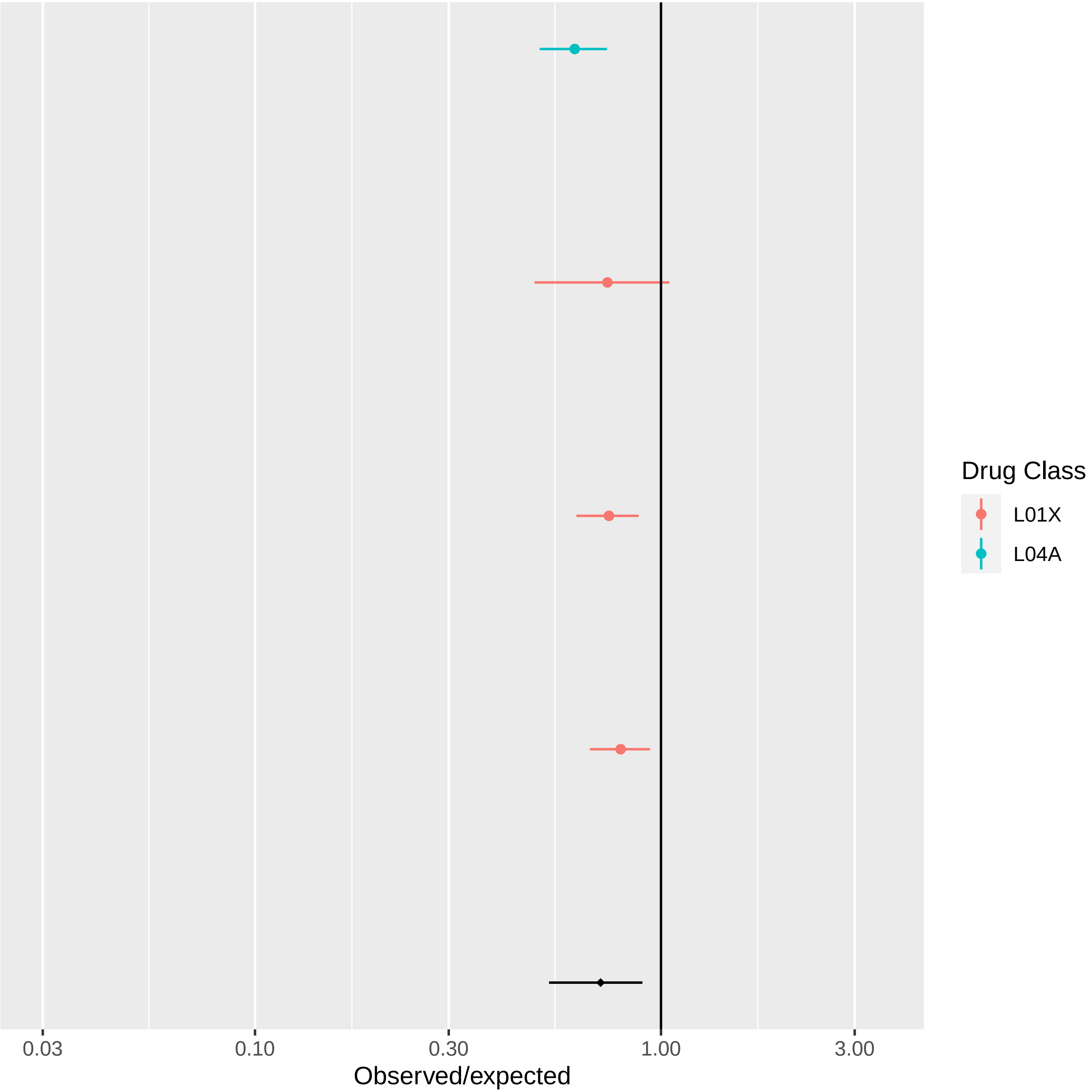

Figure S19: Observed/expected SAEs in rheumatoid arthritis trials

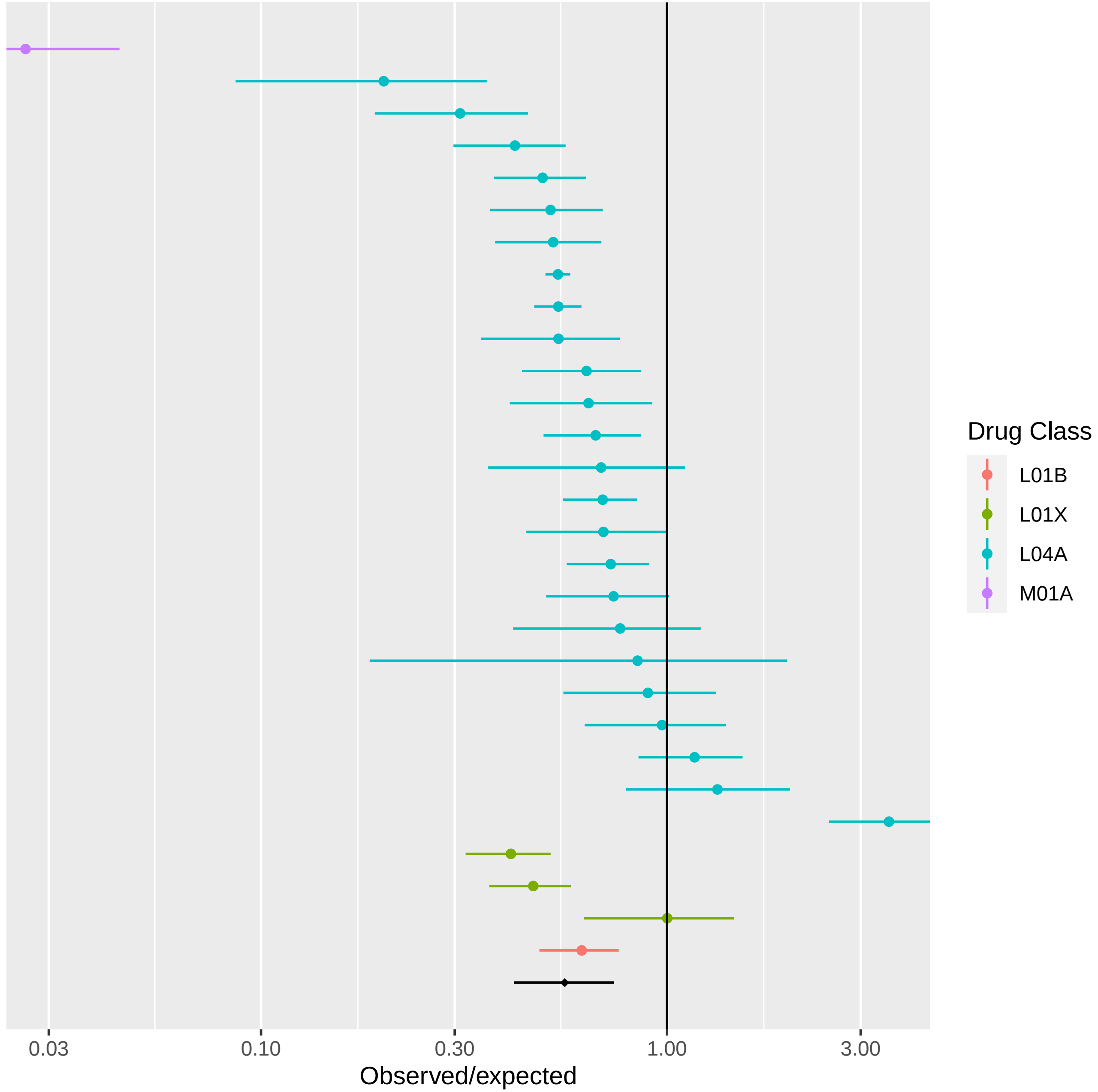

Figure S20: Observed/expected SAEs in systemic lupus erythematosus trials

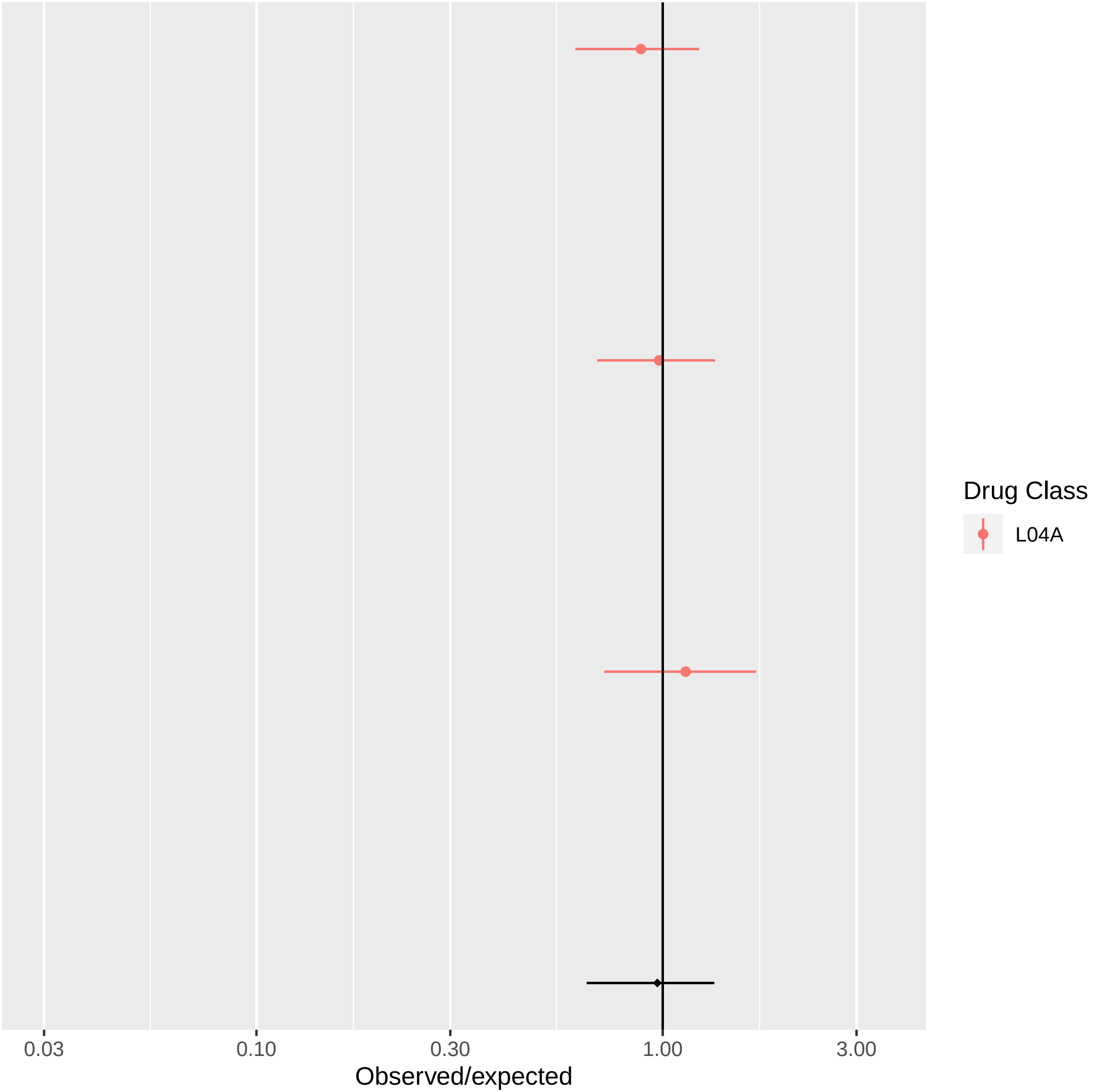

Figure S21: Observed/expected SAEs in thromboembolism trials

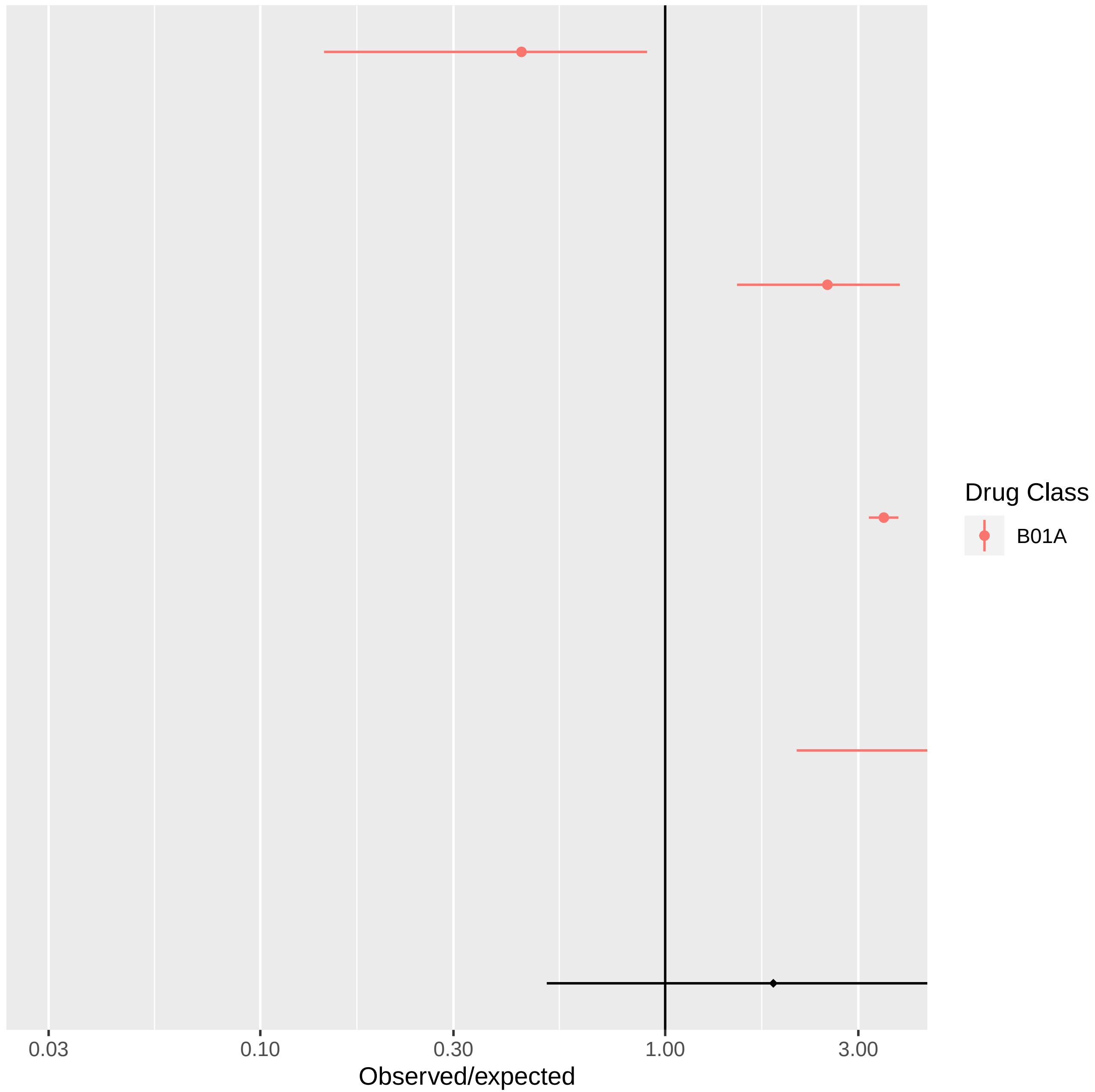

Supplement: Supplementary file 1 — Additional file 1: Supplementary figures S1-S21. Trial level estimates of observed/expected SAEs. Figure S1. Observed/expected SAEs in asthma trials. Figure S2. Observed/expected SAEs in atrial fibrillation trials. Figure S3. Observed/expected SAEs in axial spondyloarthritis trials. Figure S4. Observed/expected SAEs in benign prostatic hyperplasia trials. Figure S5. Observed/expected SAEs in dementia trials. Figure S6. Observed/expected SAEs in type 2 diabetes mellitus trials. Figure S7. Observed/expected SAEs in epilepsy trials. Figure S8. Observed/expected SAEs in hypertension trials. Figure S9. Observed/expected SAEs in pulmonary hypertension trials. Figure S10. Observed/expected SAEs in inflammatory bowel disease trials. Figure S11. Observed/expected SAEs in myocardial infarction trials. Figure S12. Observed/expected SAEs in osteoarthritis trials. Figure S13. Observed/expected SAEs in osteoporosis trials. Figure S14. Observed/expected SAEs in Parkinson’s disease trials. Figure S15. Observed/expected SAEs in psoriasis trials. Figure S16. Observed/expected SAEs in psoriatic arthropathy trials. Figure S17. Observed/expected SAEs in chronic obstructive pulmonary disease trials. Figure S18. Observed/expected SAEs in pulmonary fibrosis trials. Figure S19. Observed/expected SAEs in rheumatoid arthritis trials. Figure S20. Observed/expected SAEs in systemic lupus erythematosus trials. Figure S21. Observed/expected SAEs in thromboembolism trials. [file 12916_2022_2594_MOESM1_ESM.pdf]
