# Supplementary material for: Assessing trial representativeness using serious adverse events: an observational analysis using aggregate and individual-level data from clinical trials and routine healthcare data
Source: BMC Med. 2022 Oct 28;20:410. doi: 10.1186/s12916-022-02594-9 (PMC9615407; doi:10.1186/s12916-022-02594-9)

Additional file 2:
Supplementary figures S22-23 - Comparison of SAEs between trial arms

Figure S22: Trial-level comparison of SAE rate between trial arms

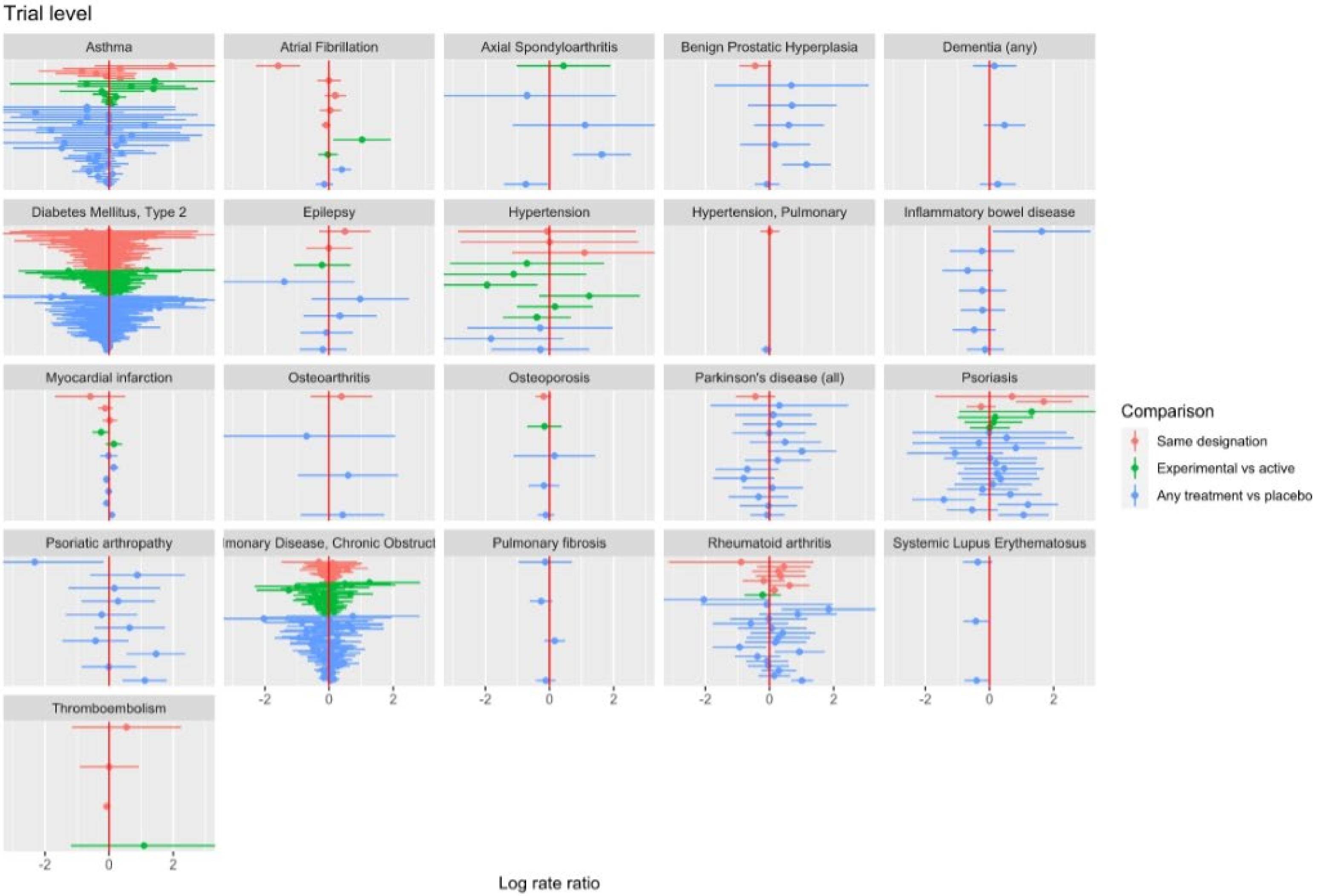

Figure S23: Index condition-level meta-analyses of comparison of SAE rate between trial arms

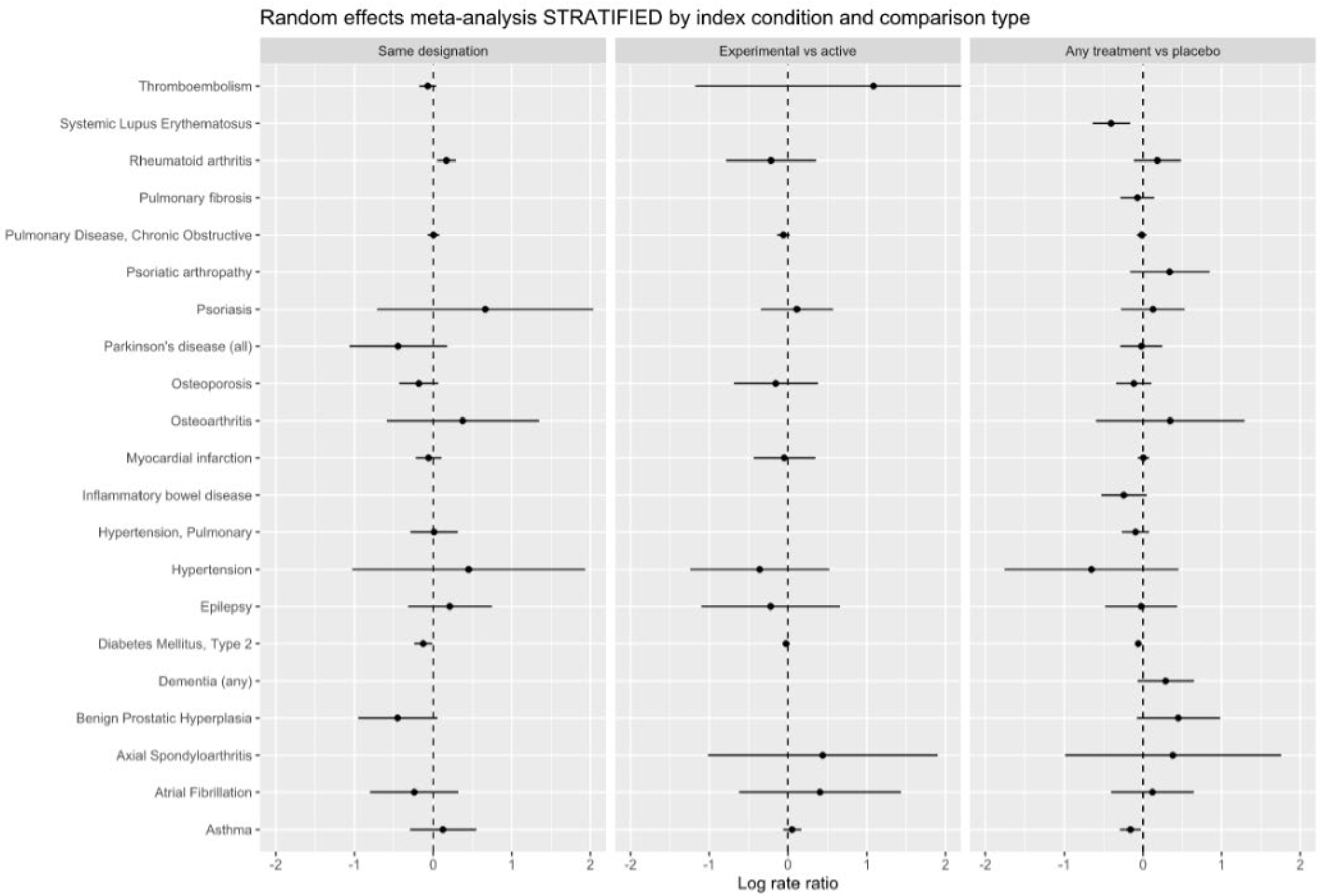

Supplement: Supplementary file 2 — Additional file 2: Supplementary figures S22-23 Comparison of SAEs between trial arms. Figure S22. Trial-level comparison of SAE rate between trial arms. Figure S23. Index condition-level meta-analyses of comparison of SAE rate between trial arms. [file 12916_2022_2594_MOESM2_ESM.pdf]
