# Supplementary material for: Assessing trial representativeness using serious adverse events: an observational analysis using aggregate and individual-level data from clinical trials and routine healthcare data
Source: BMC Med. 2022 Oct 28;20:410. doi: 10.1186/s12916-022-02594-9 (PMC9615407; doi:10.1186/s12916-022-02594-9)

Additional file 3:
Supplementary figures S24-S44 - Observed/expected SAEs before and after standardization by multimorbidity count

Figure S24: observed/expected SAEs before and after standardization by multimorbidity count: asthma trials

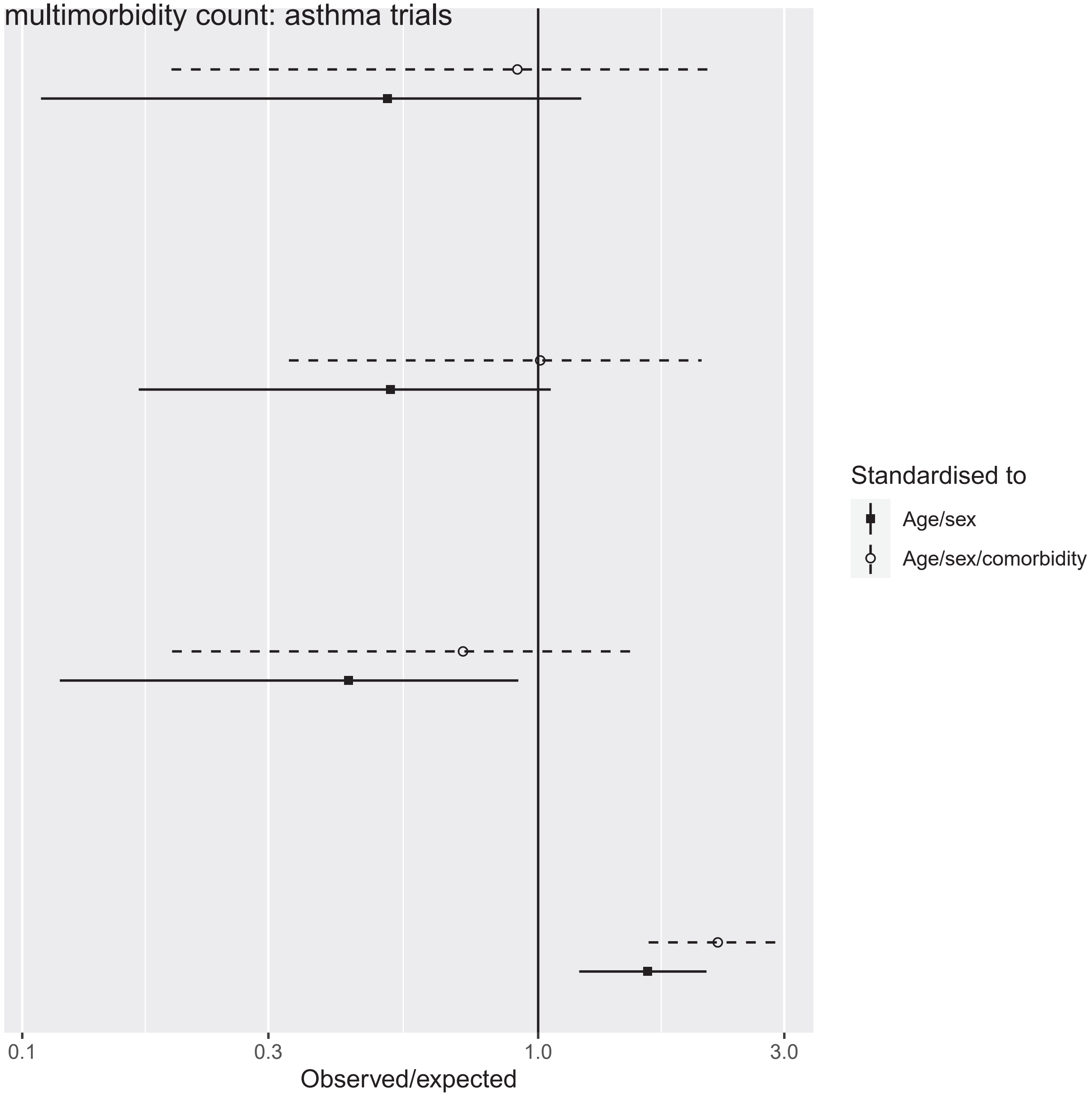

Figure S25: observed/expected SAEs before and after standardization by multimorbidity count: atrial fibrillation trials

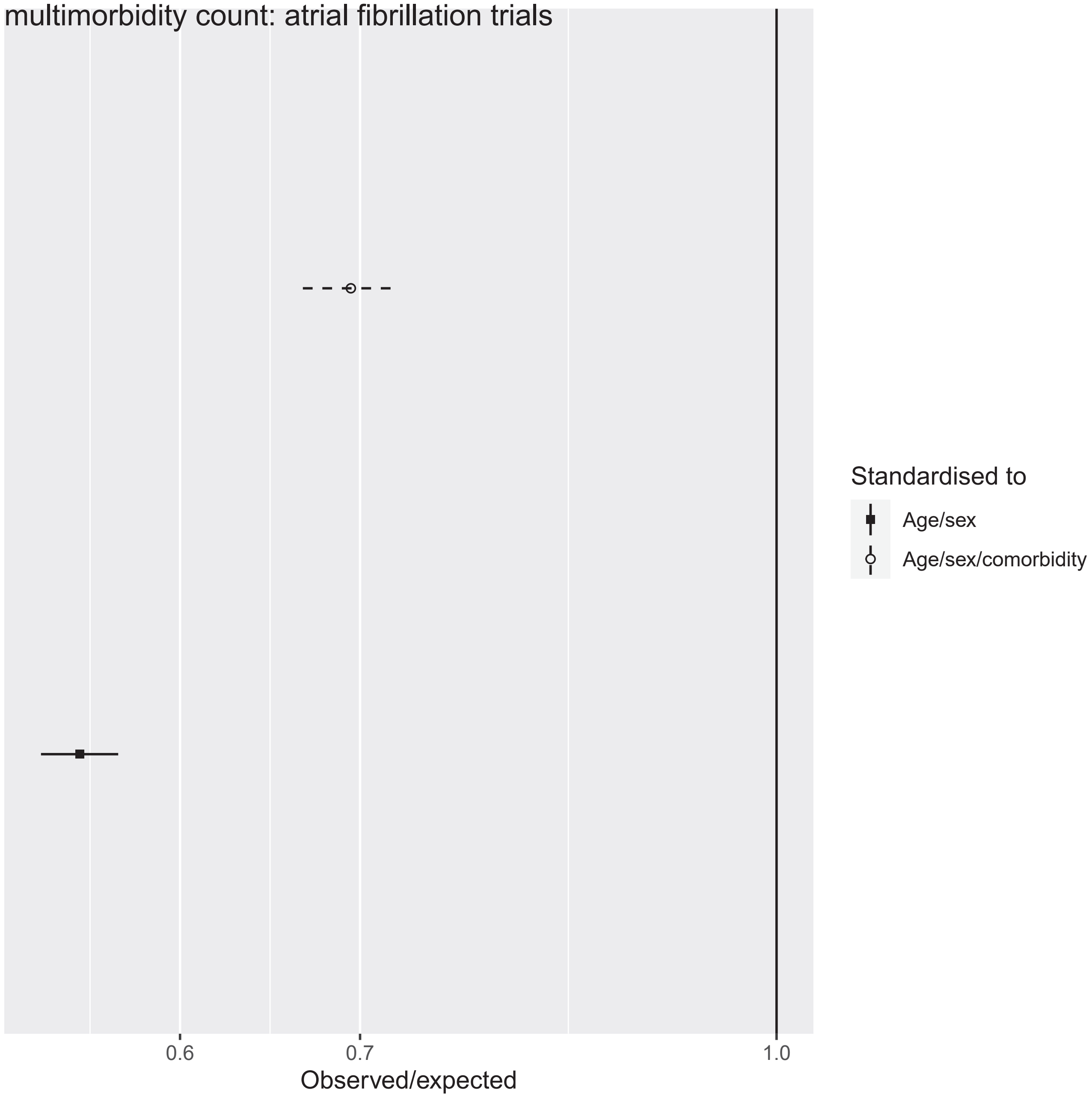

Figure S26: observed/expected SAEs before and after standardization by multimorbidity count: axial spondyloarthritis trials

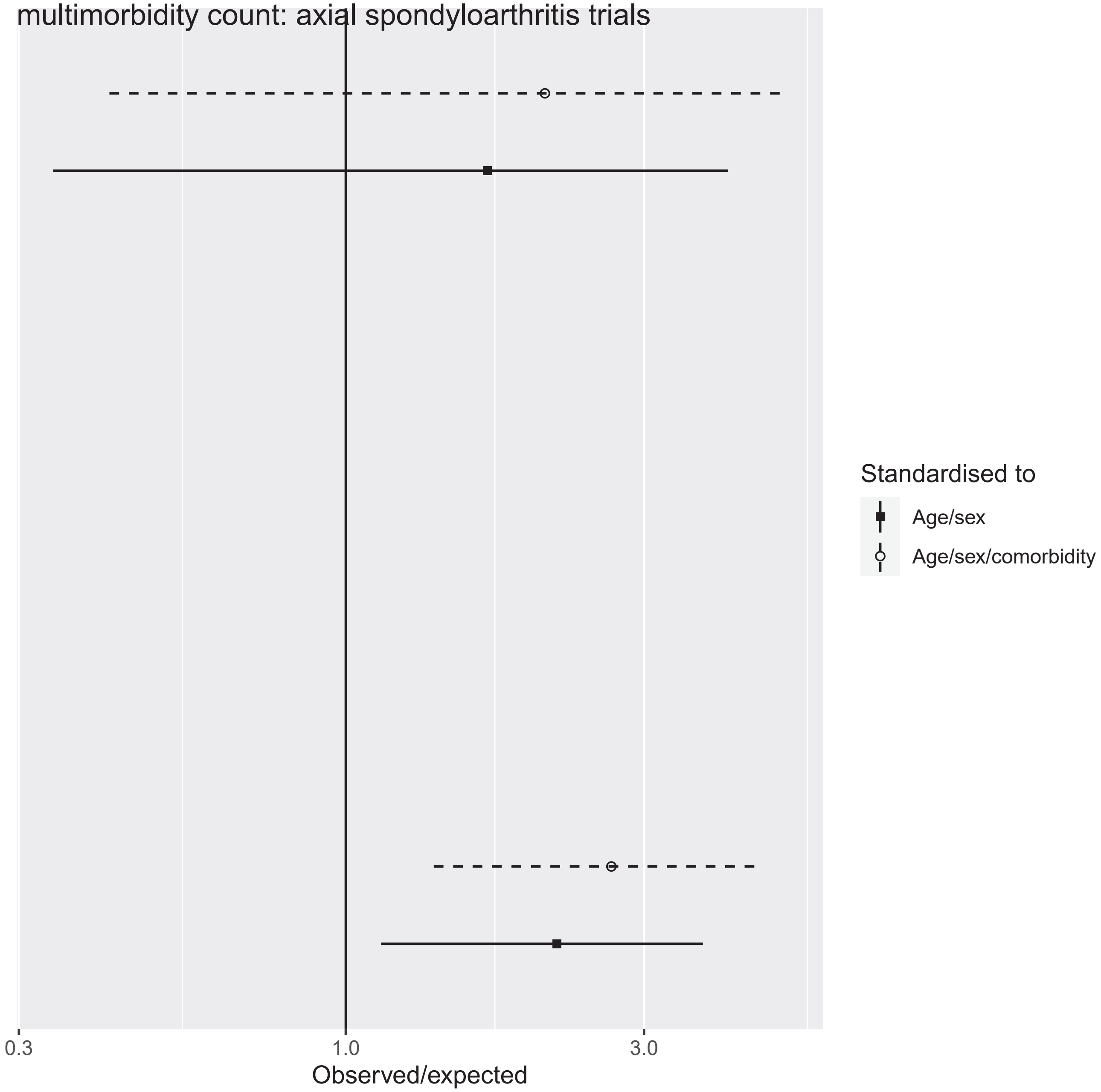

Figure S27: observed/expected SAEs before and after standardization by multimorbidity count: benign prostatic hyperplasia trials

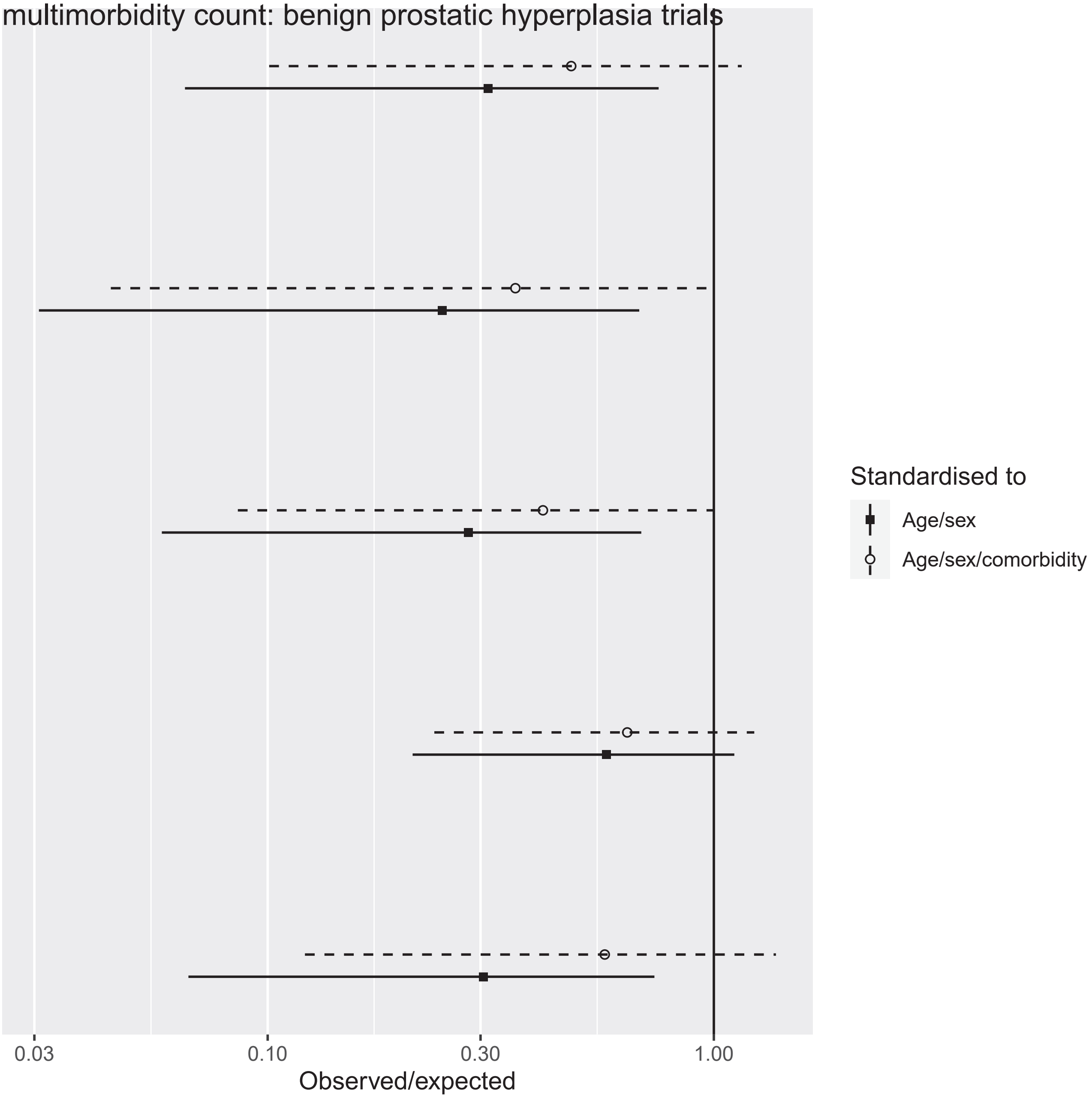

Figure S28: observed/expected SAEs before and after standardization by multimorbidity count: dementia trials

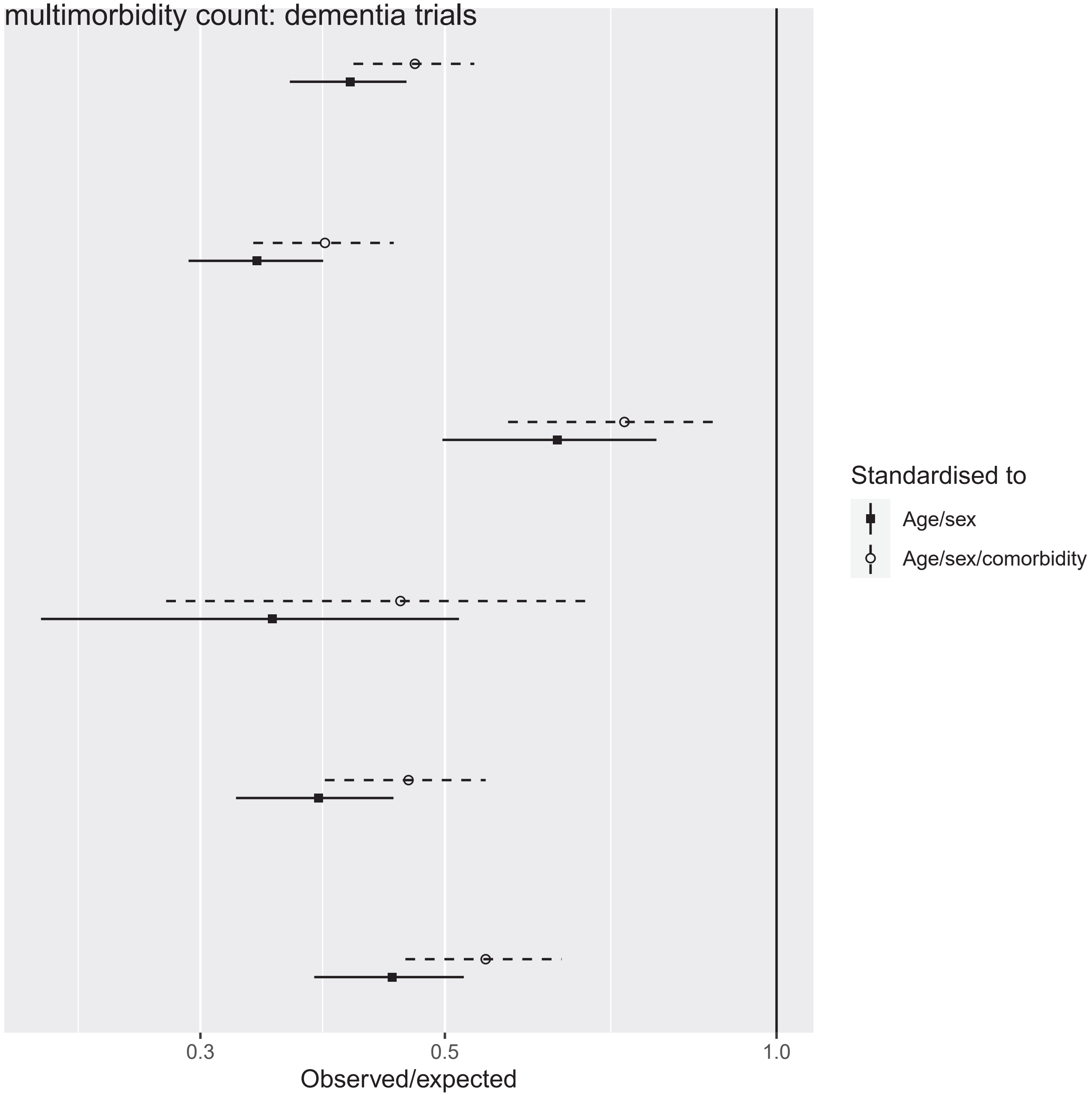

Figure S29: observed/expected SAEs before and after standardization by multimorbidity count: type 2 diabetes mellitus trials

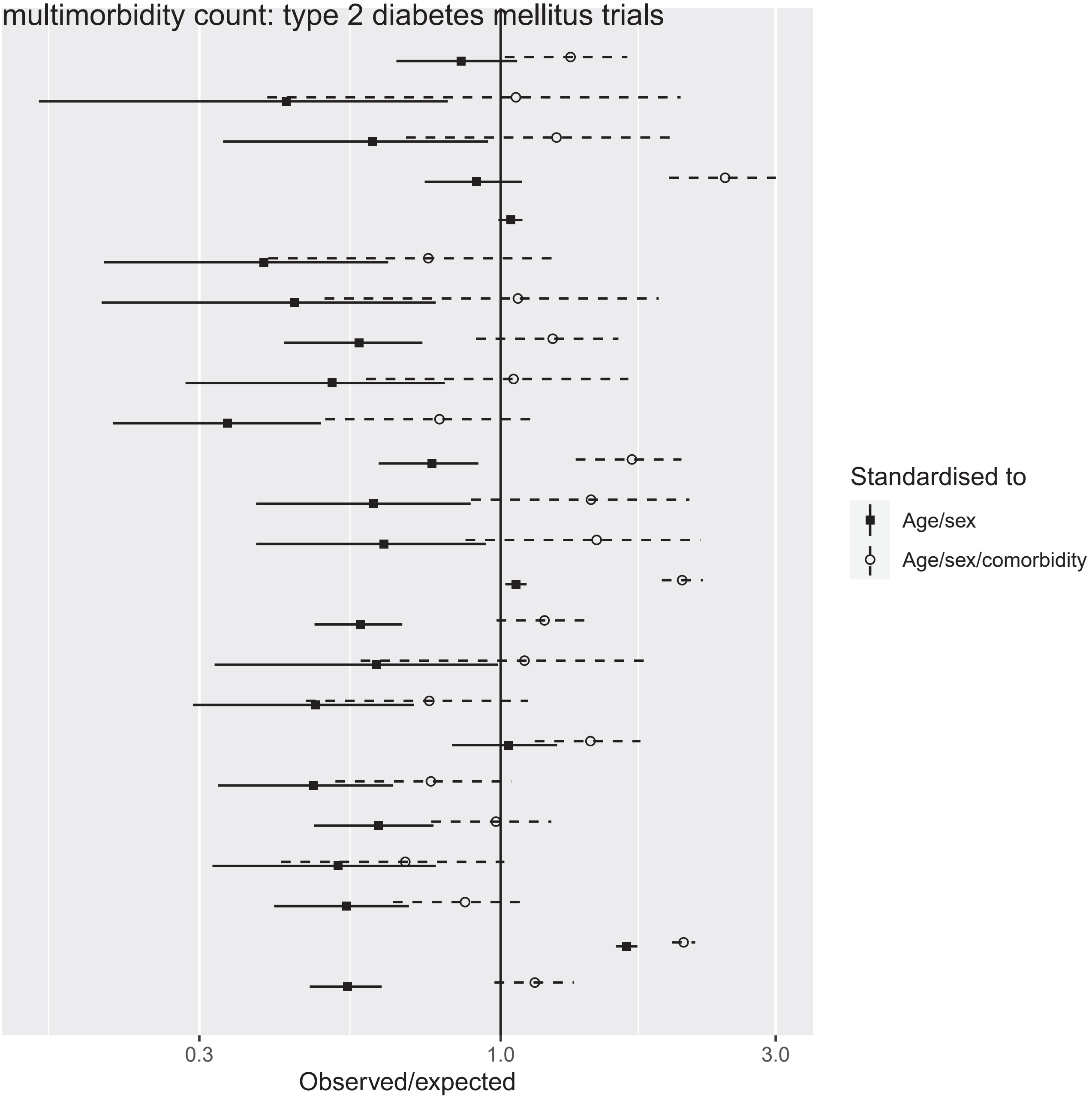

Figure S30: observed/expected SAEs before and after standardization by multimorbidity count: hypertension trials

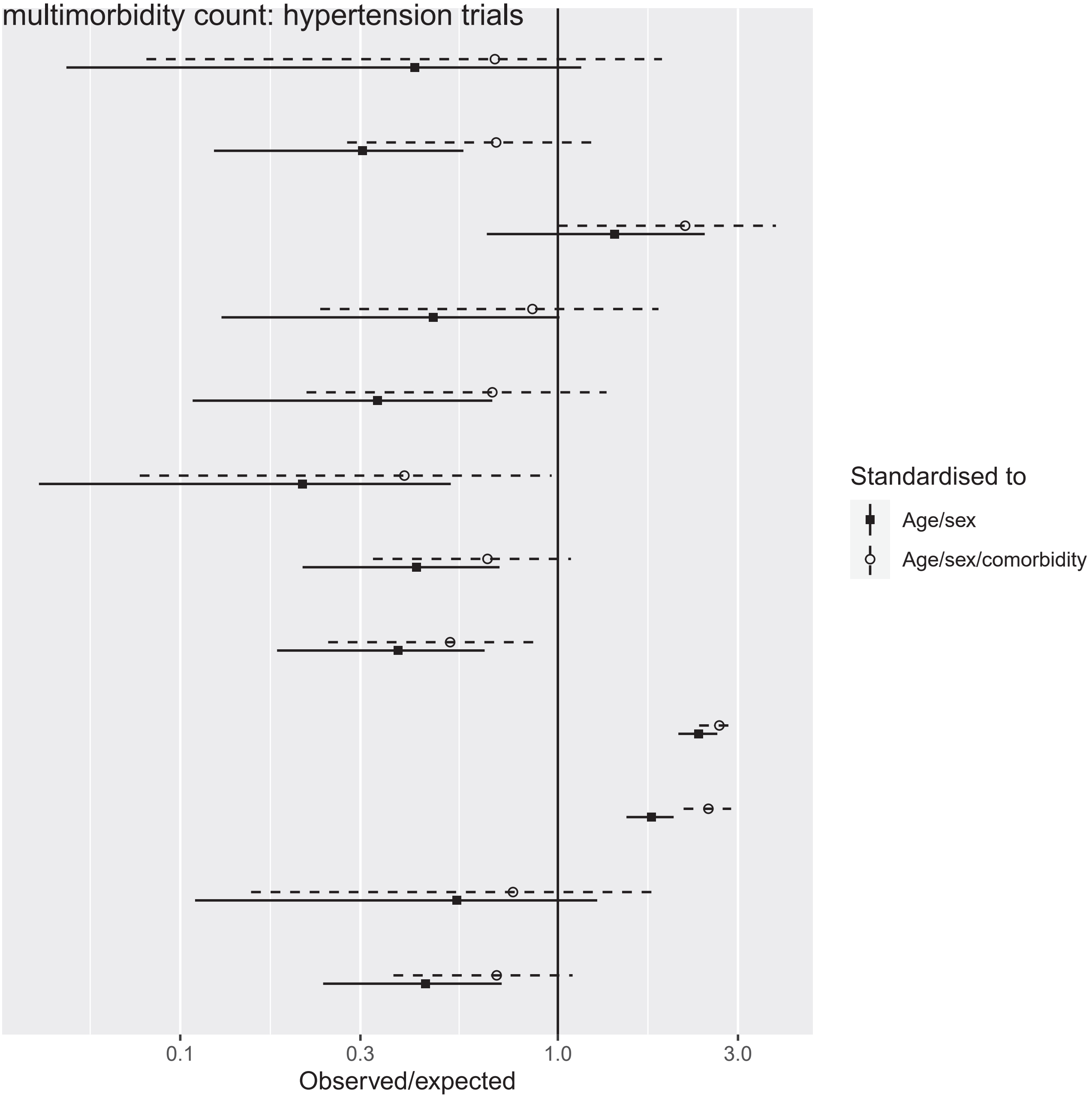

Figure S31: observed/expected SAEs before and after standardization by multimorbidity count: pulmonary hypertension trials

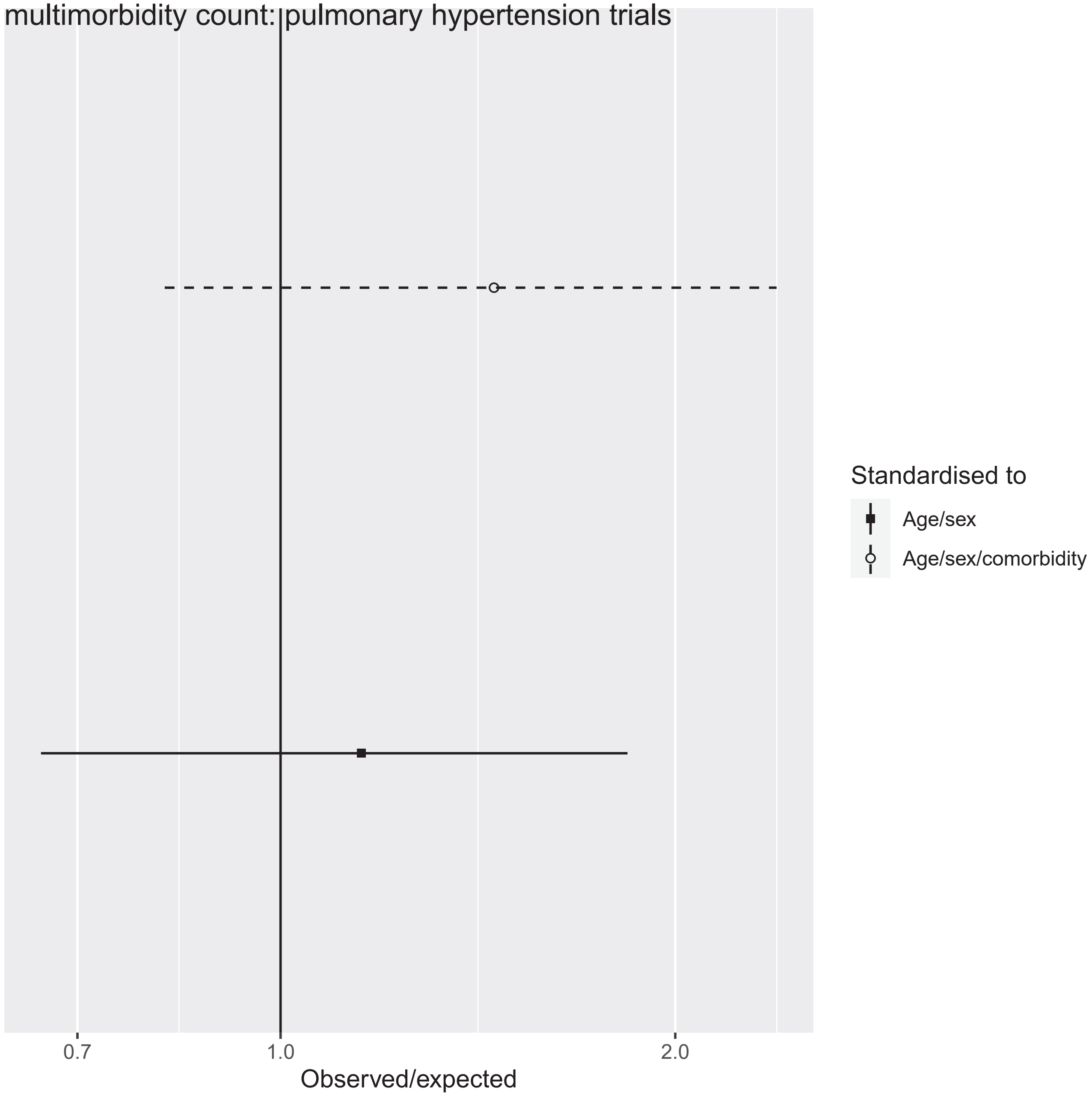

Figure S32: observed/expected SAEs before and after standardization by multimorbidity count: inflammatory bowel disease trials

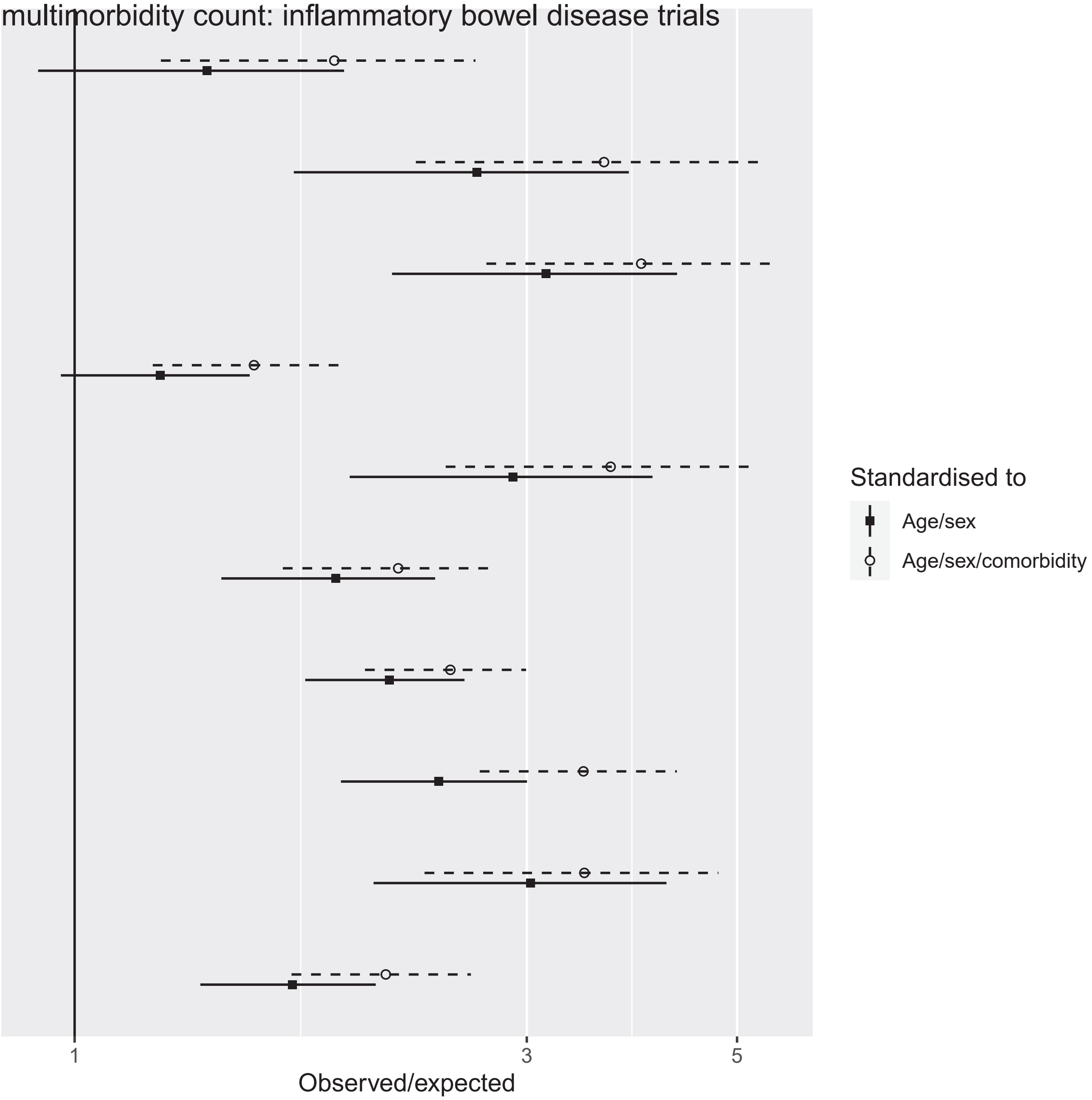

Figure S33: observed/expected SAEs before and after standardization by multimorbidity count: migraine trials

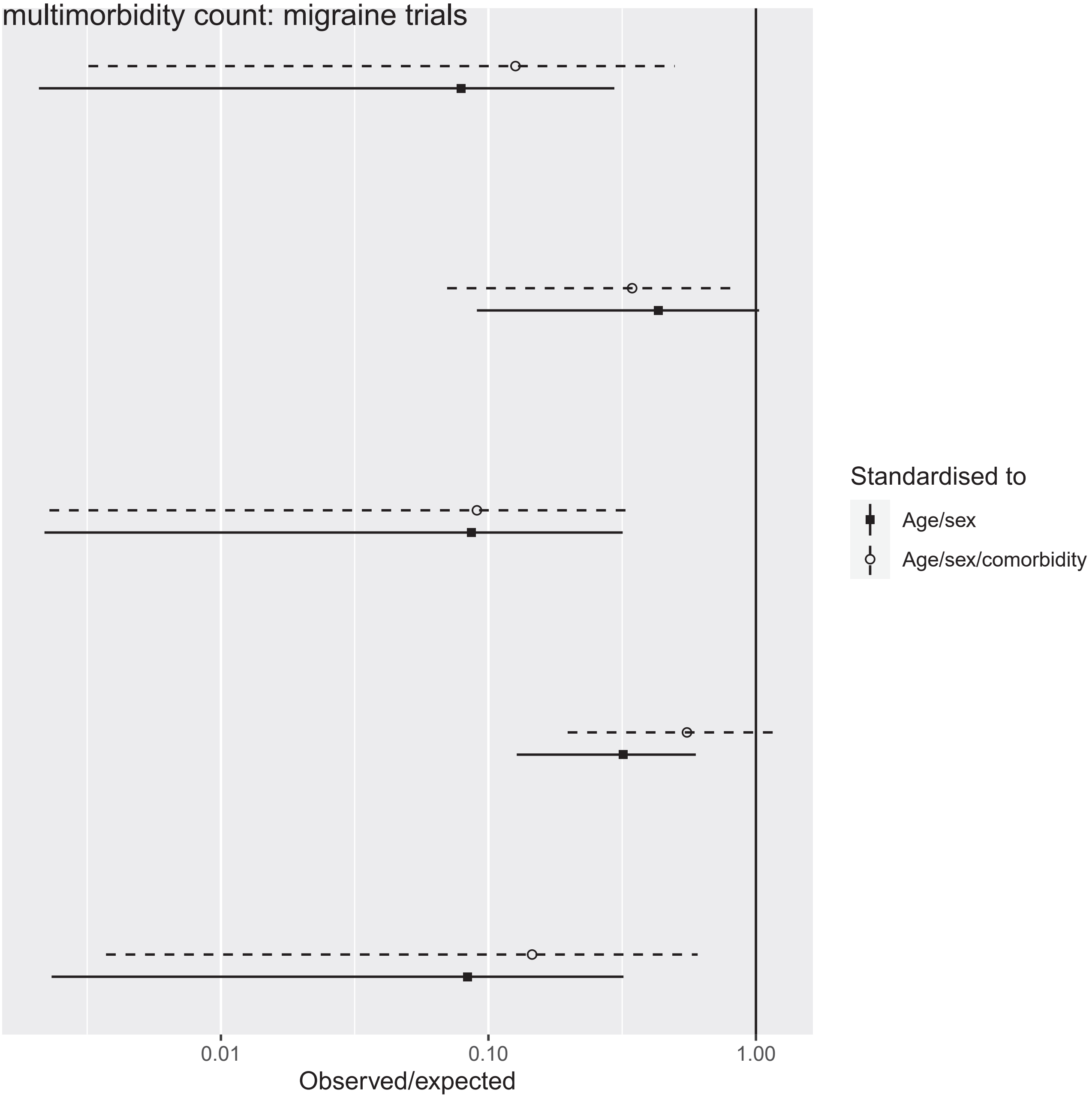

Figure S34: observed/expected SAEs before and after standardization by multimorbidity count: osteoarthritis trials

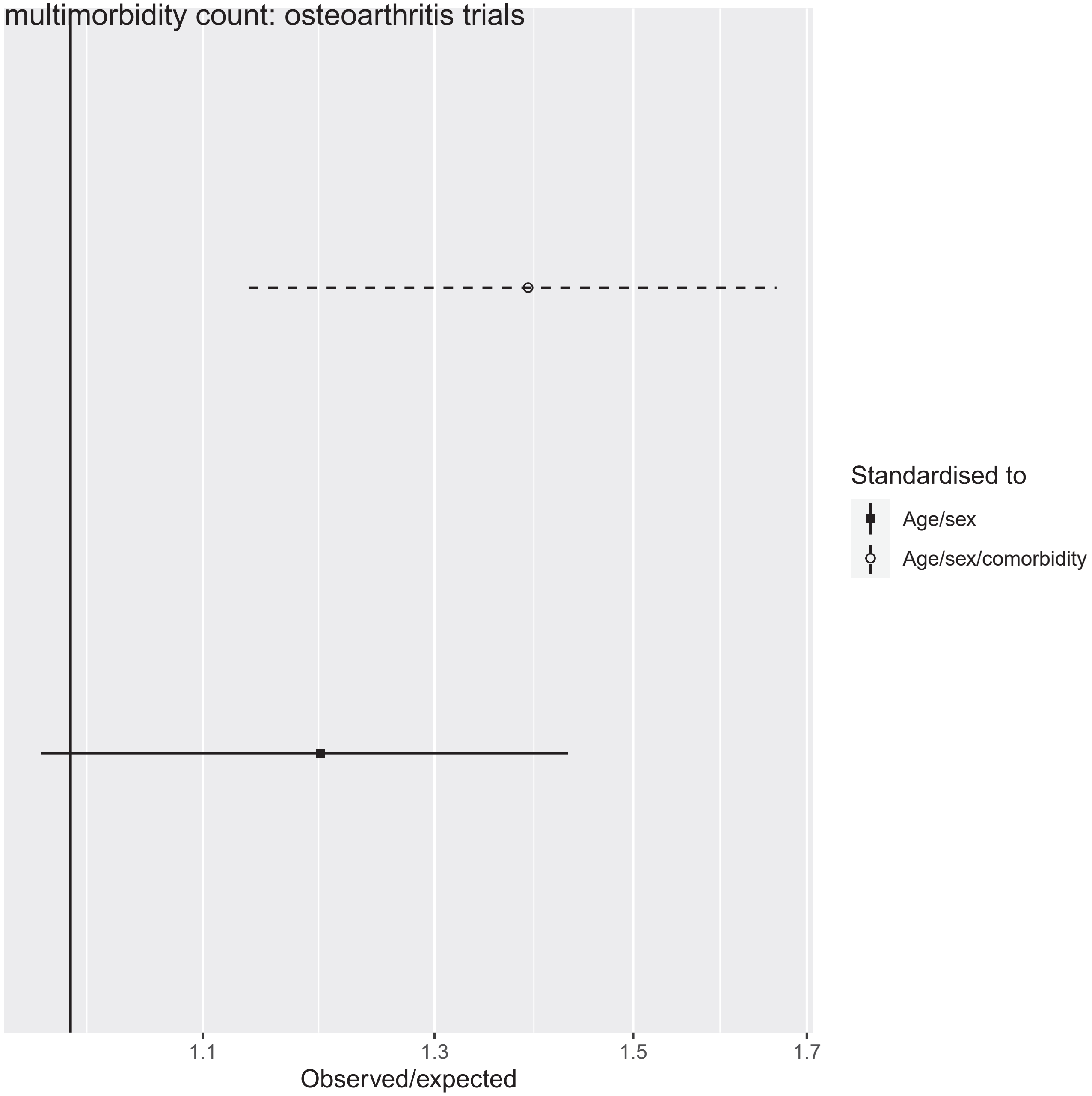

Figure S35: observed/expected SAEs before and after standardization by multimorbidity count: osteoporosis trials

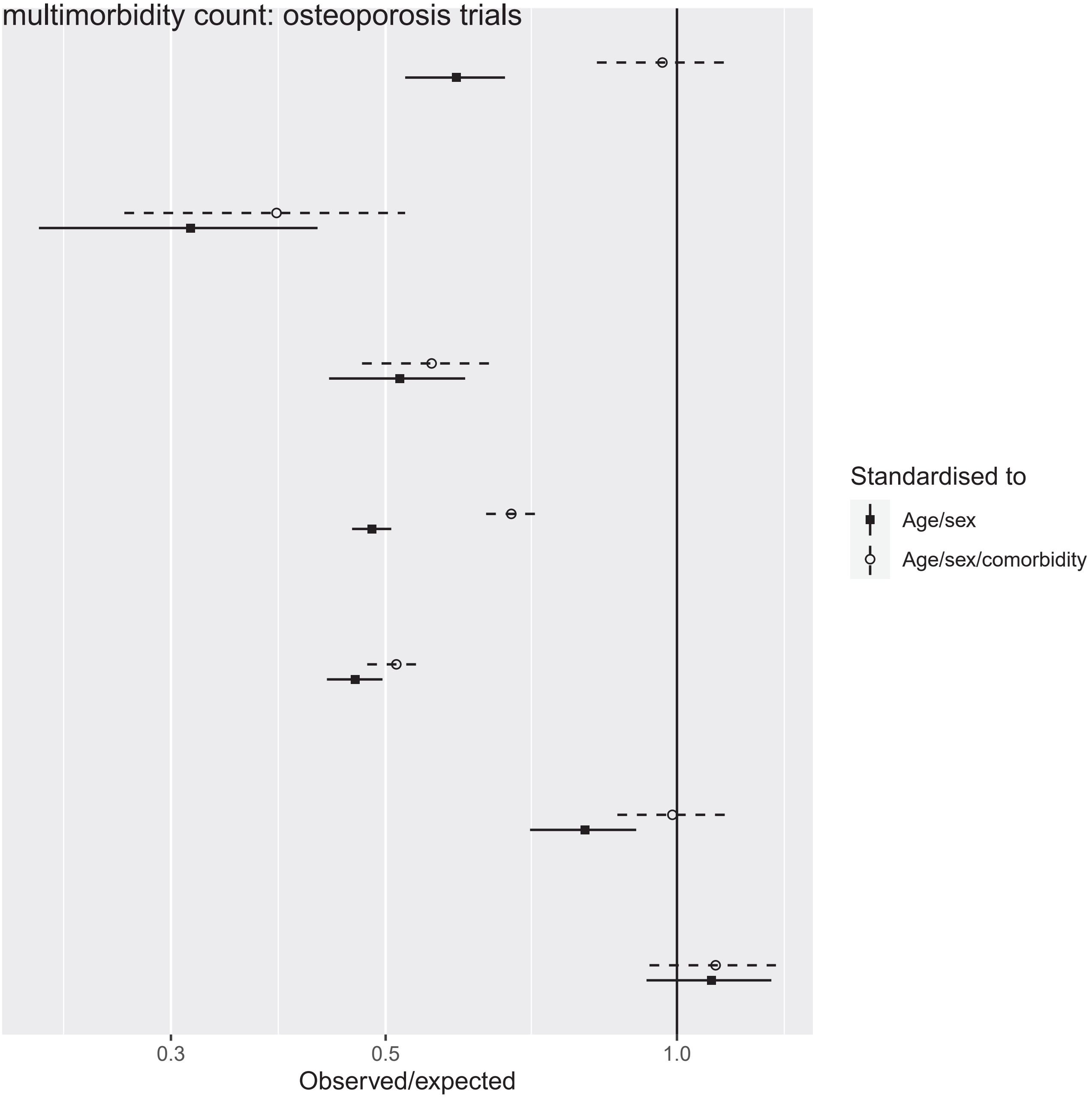

Figure S36: observed/expected SAEs before and after standardization by multimorbidity count: Parkinson’s disease trials

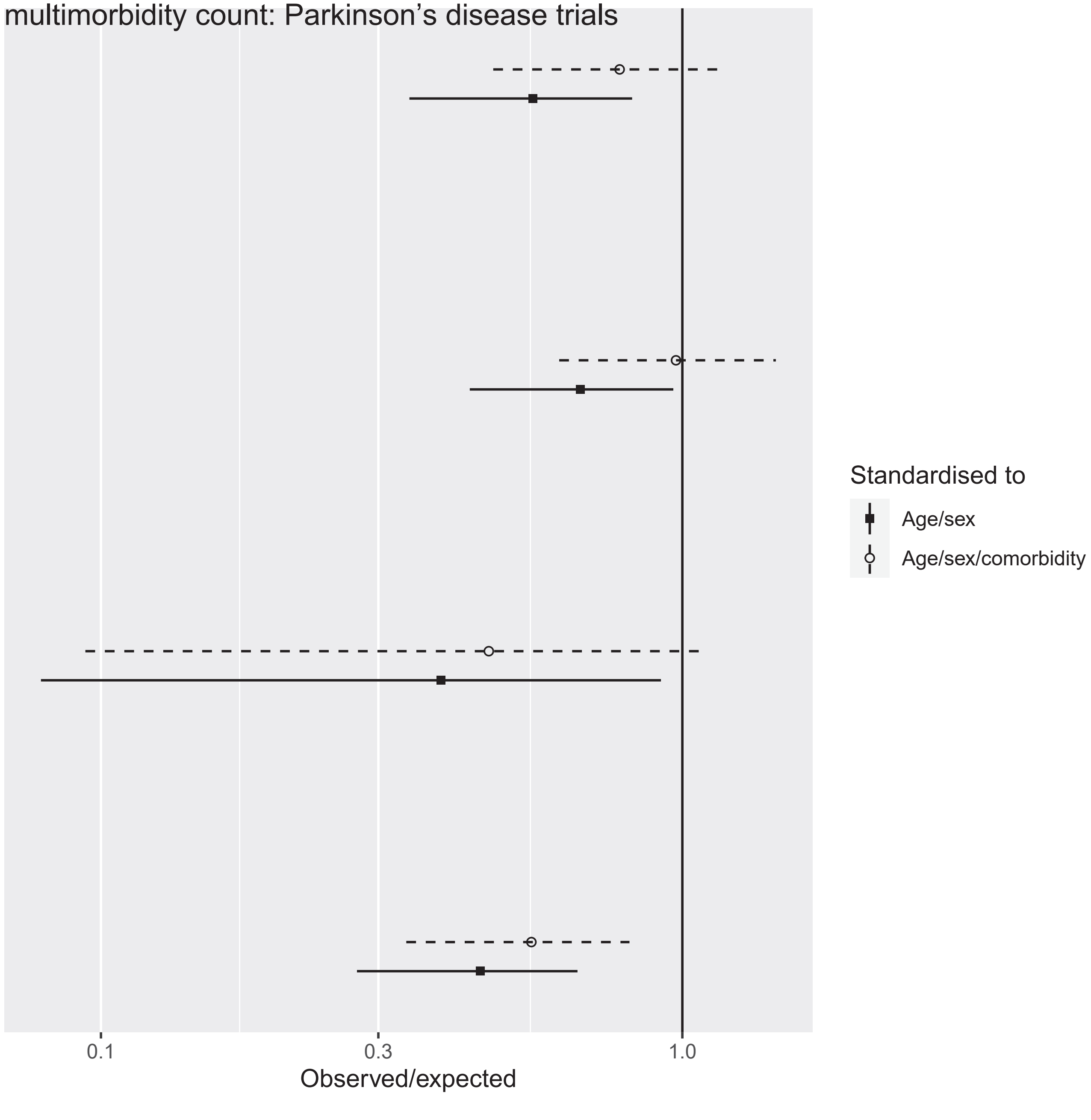

Figure S37: observed/expected SAEs before and after standardization by multimorbidity count: psoriasis trials

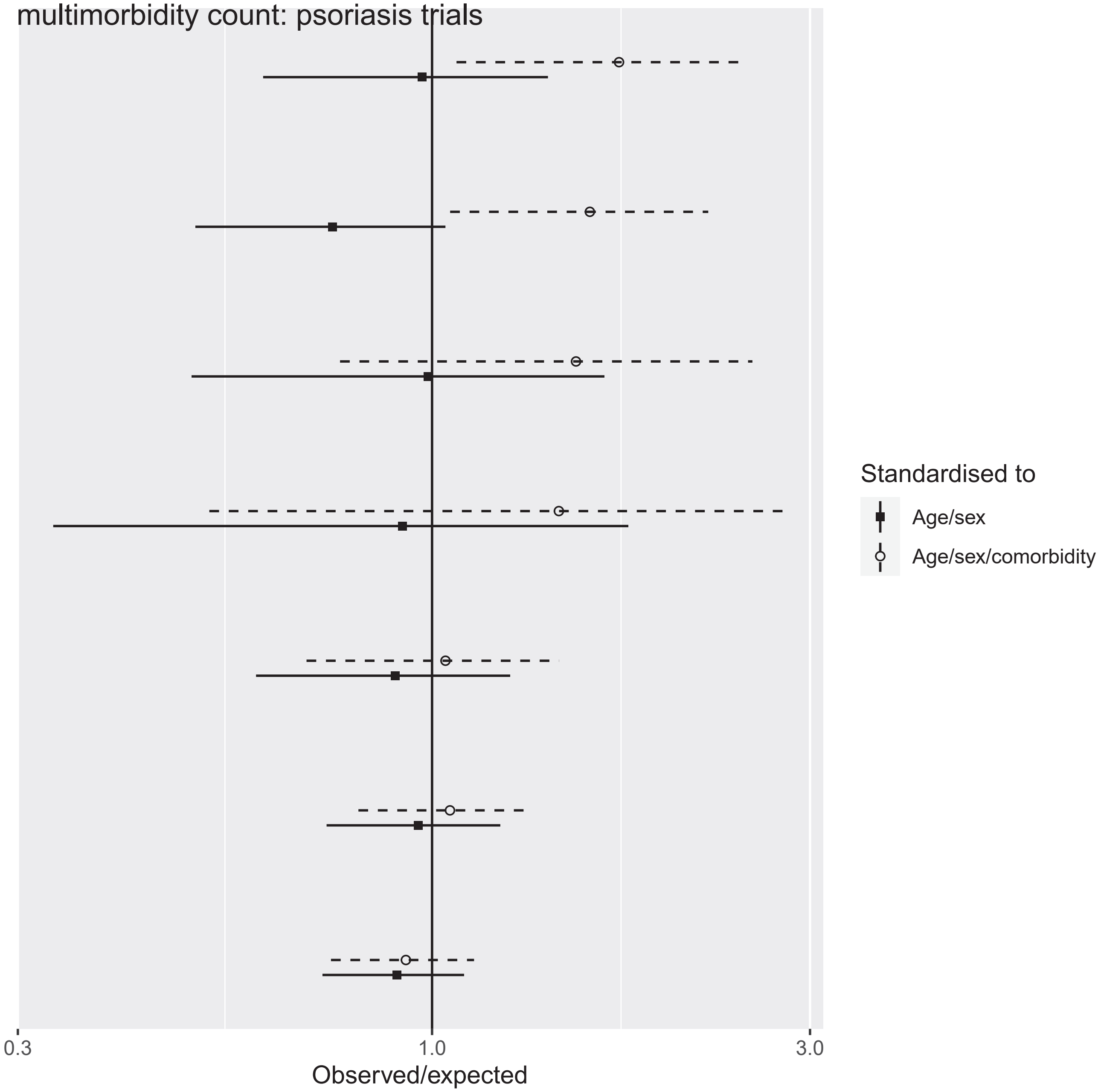

Figure S3: observed/expected SAEs before and after standardization by multimorbidity count: psoriatic arthropathy trials

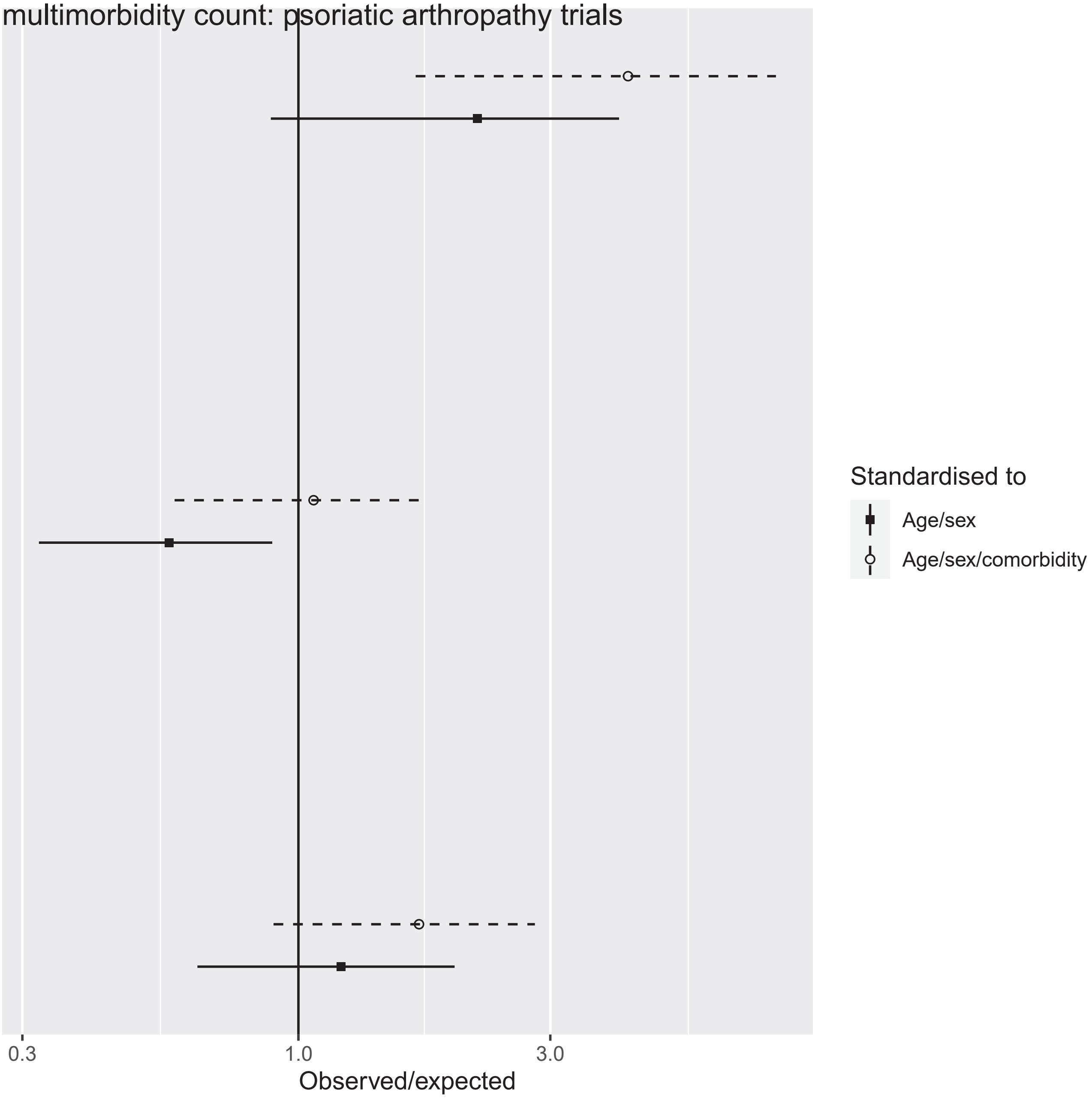

Figure S39: observed/expected SAEs before and after standardization by multimorbidity count: chronic obstructive pulmonary disease trials

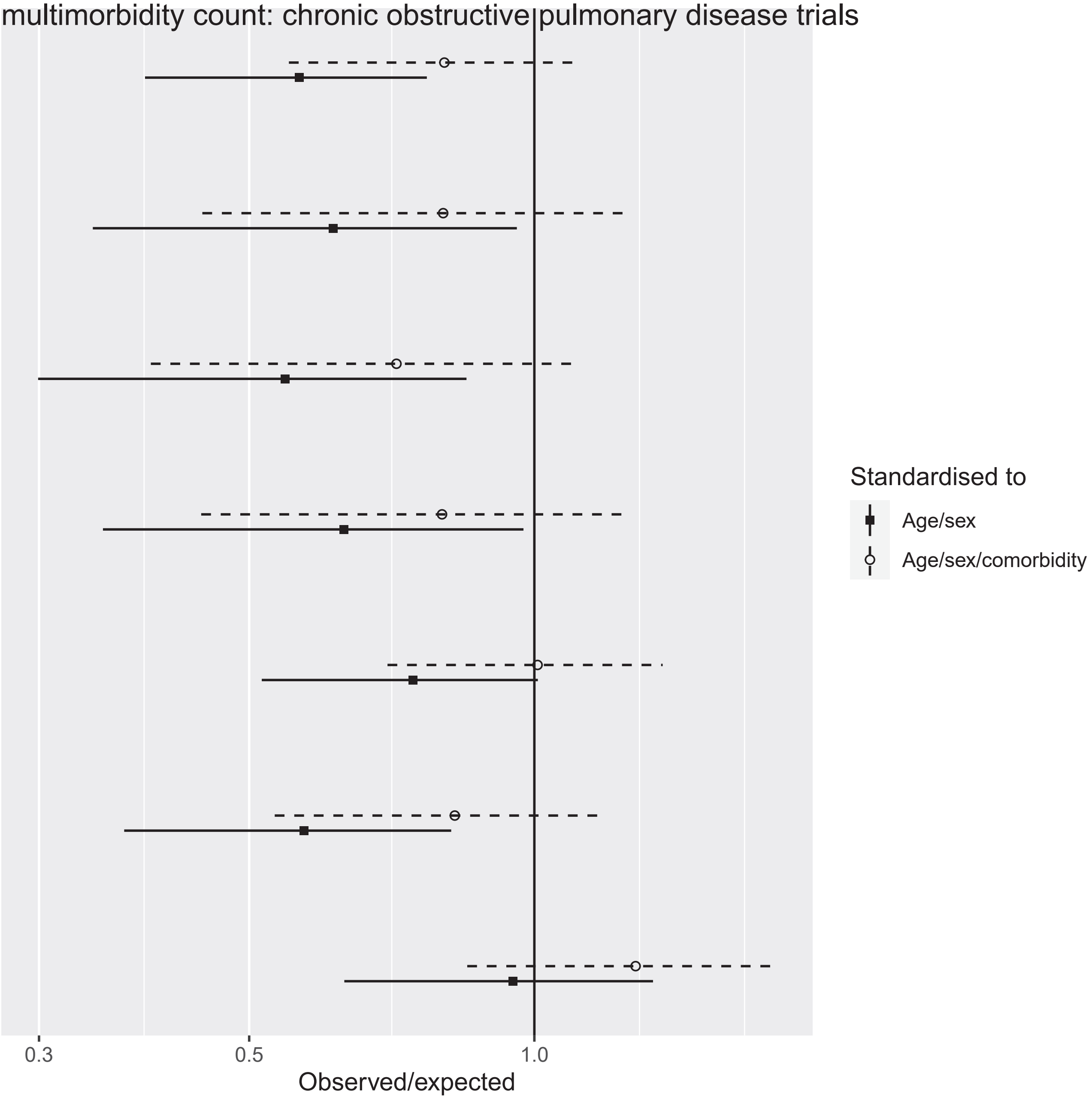

Figure S40: observed/expected SAEs before and after standardization by multimorbidity count: pulmonary fibrosis trials

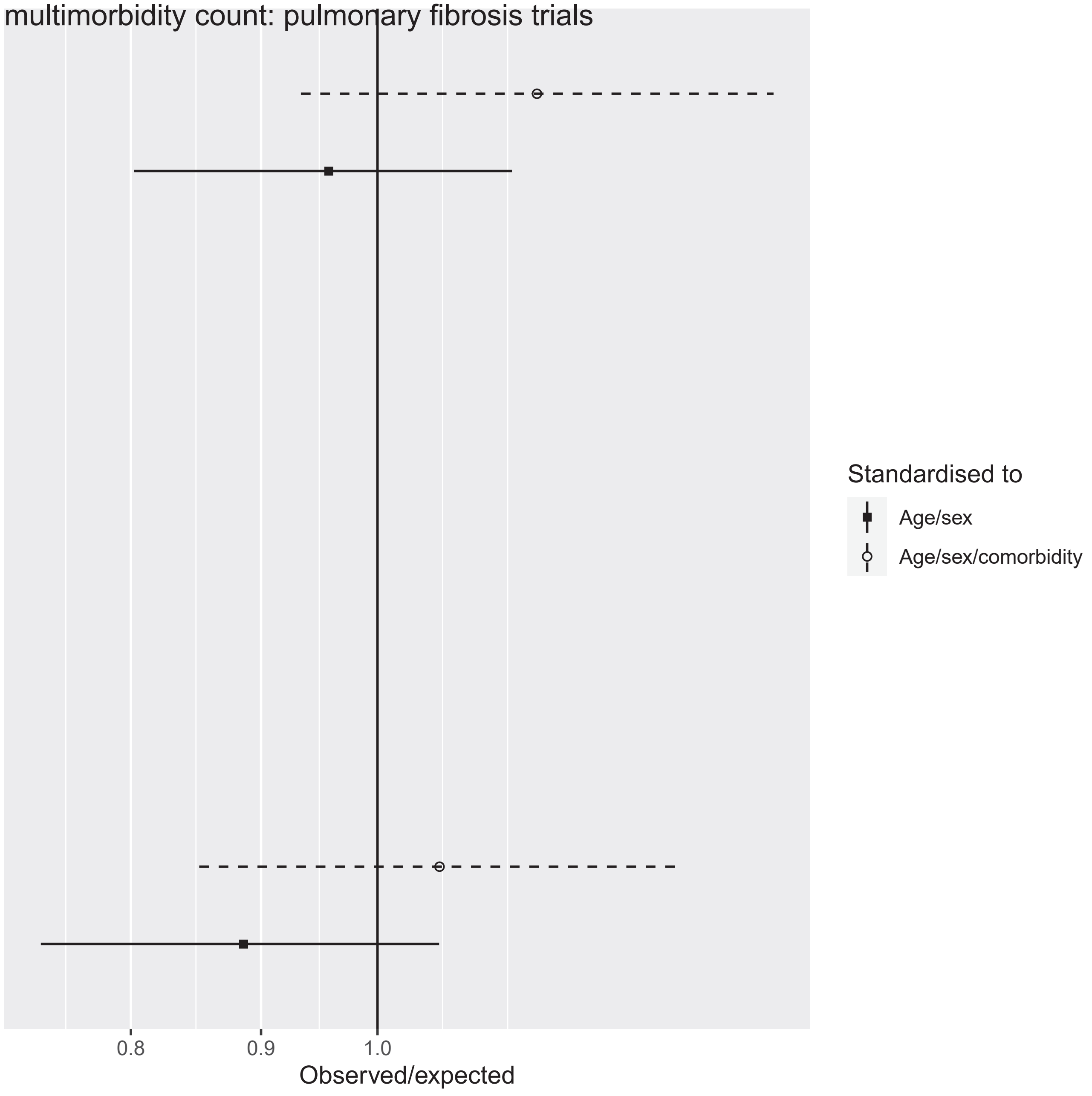

Figure S41: observed/expected SAEs before and after standardization by multimorbidity count: restless legs syndrome trials

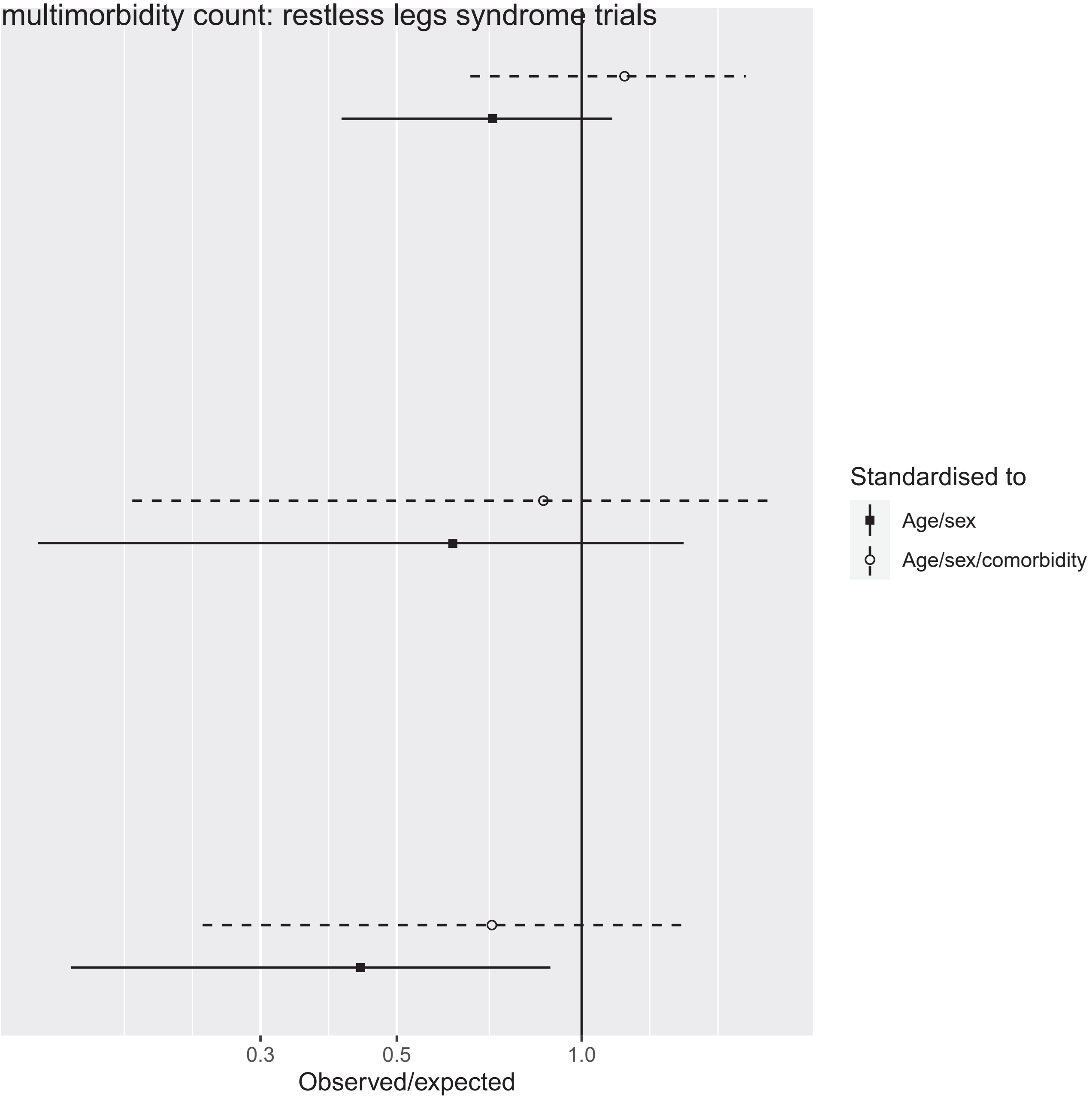

Figure S42: observed/expected SAEs before and after standardization by multimorbidity count: rheumatoid arthritis trials

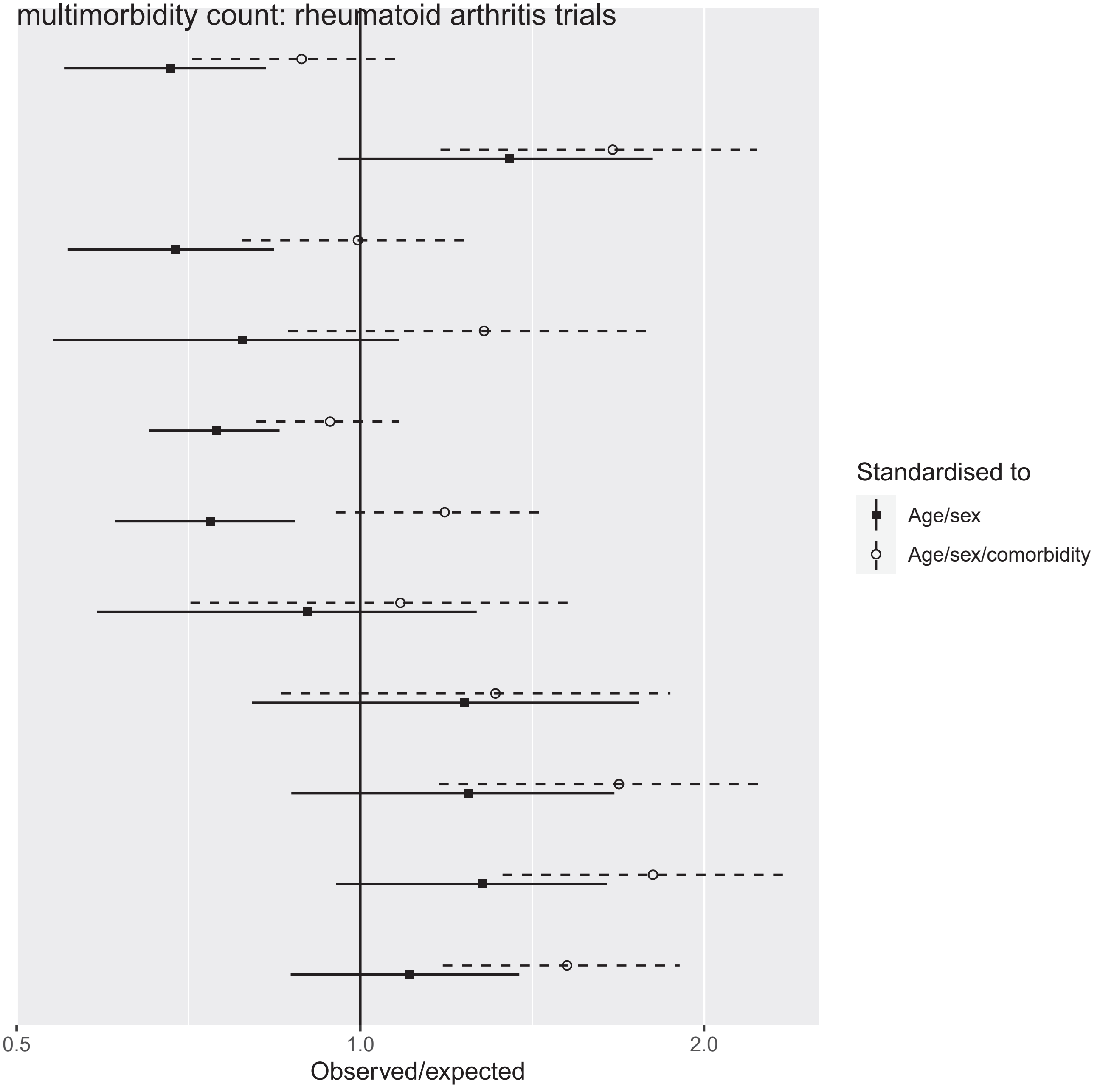

Figure S43: observed/expected SAEs before and after standardization by multimorbidity count: systemic lupus erythematosus trials

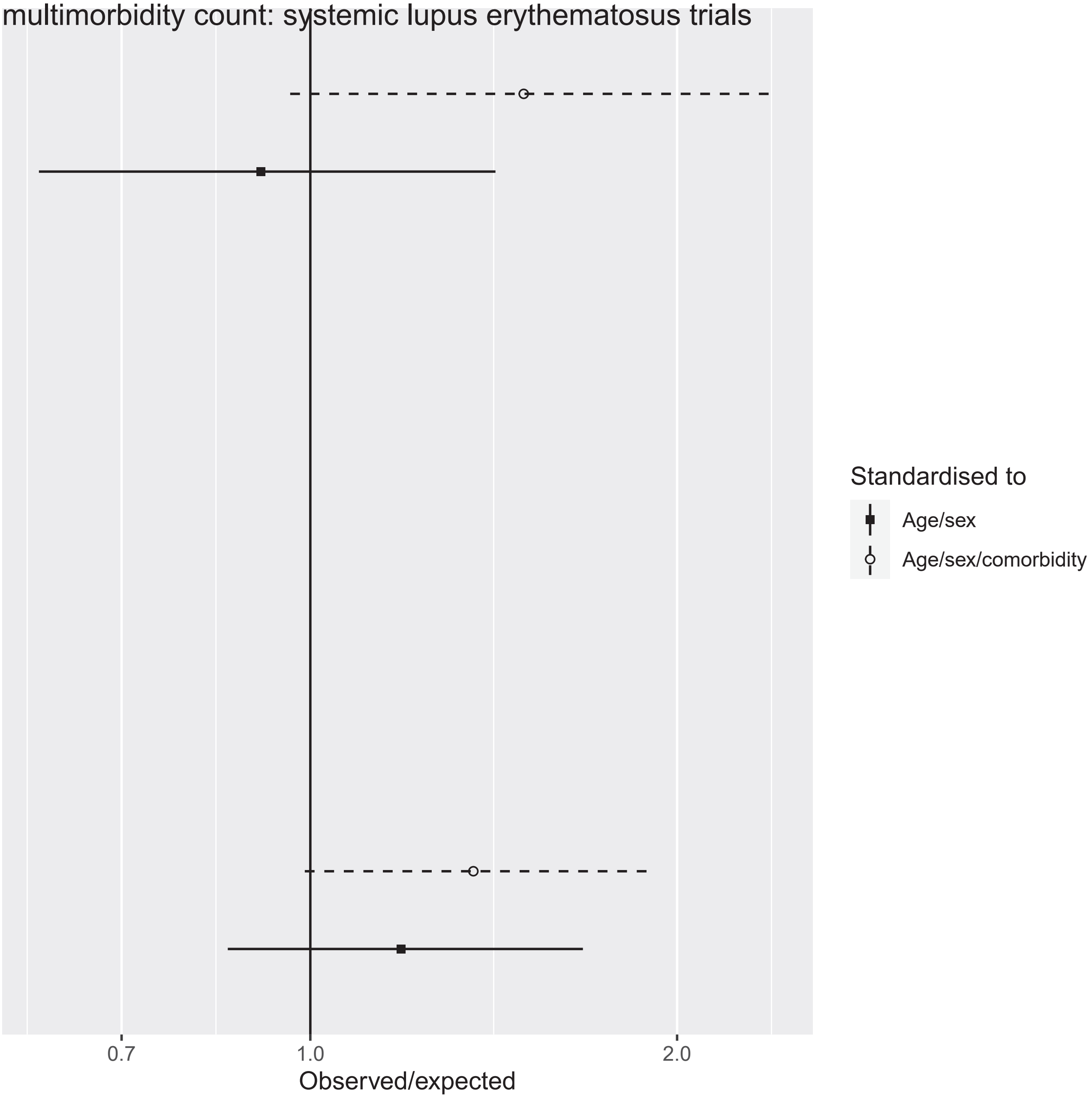

Figure S44: observed/expected SAEs before and after standardization by multimorbidity count: thromboembolism trials

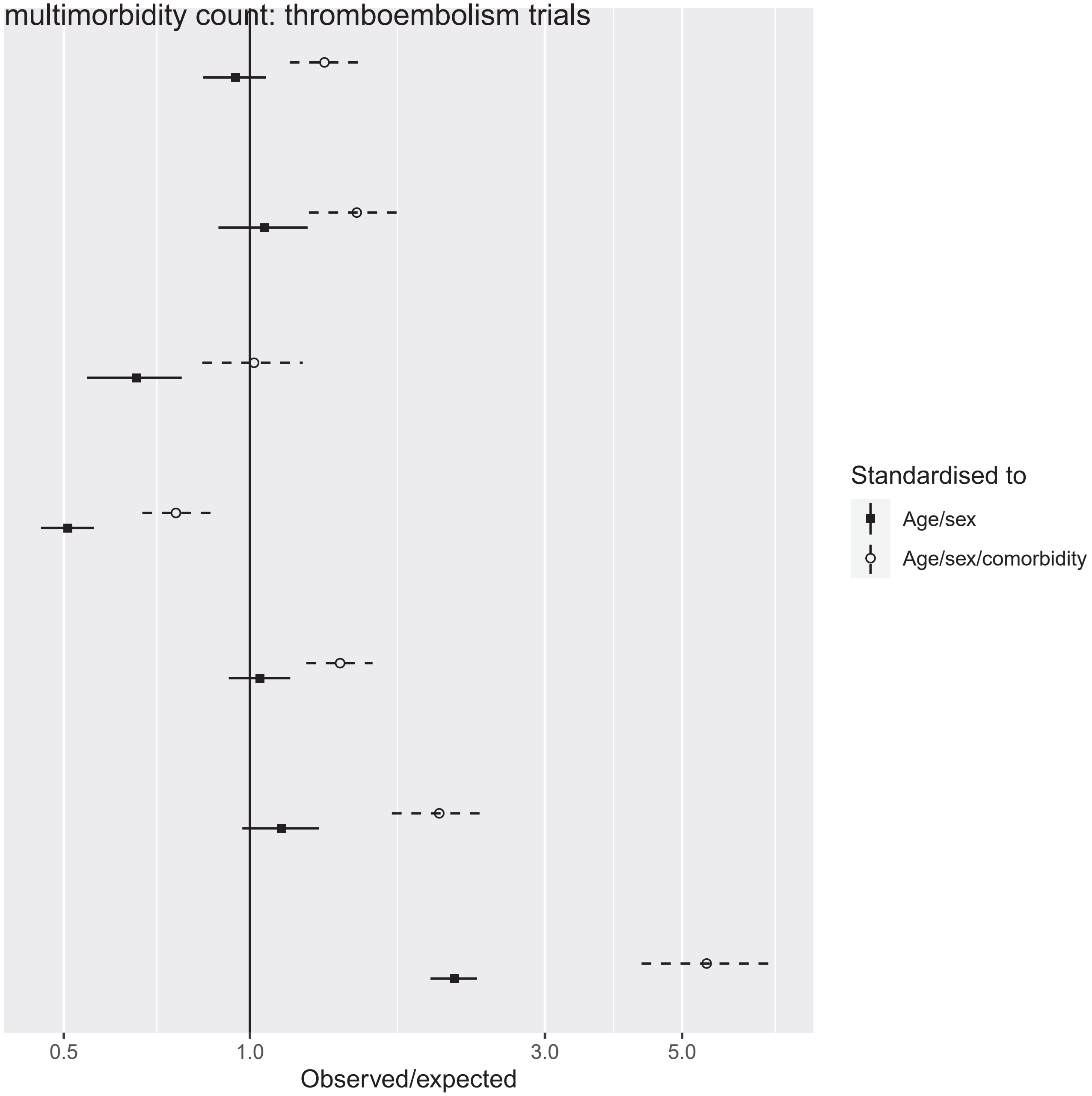

Supplement: Supplementary file 3 — Additional file 3: Supplementary figures S24-S44. Observed/expected SAEs before and after standardization by multimorbidity count. Figure S24. Observed/expected SAEs before and after standardization by multimorbidity count: asthma trials. Figure S25. Observed/expected SAEs before and after standardization by multimorbidity count: atrial fibrillation trials. Figure S26. observed/expected SAEs before and after standardization by multimorbidity count: axial spondyloarthritis trials. Figure S27. Observed/expected SAEs before and after standardization by multimorbidity count: benign prostatic hyperplasia trials. Figure S28. Observed/expected SAEs before and after standardization by multimorbidity count: dementia trials. Figure S29. observed/expected SAEs before and after standardization by multimorbidity count: type 2 diabetes mellitus trials. Figure S30. Observed/expected SAEs before and after standardization by multimorbidity count: hypertension trials. Figure S31. Observed/expected SAEs before and after standardization by multimorbidity count: pulmonary hypertension trials. Figure S32. Observed/expected SAEs before and after standardization by multimorbidity count: inflammatory bowel disease trials. Figure S33. Observed/expected SAEs before and after standardization by multimorbidity count: migraine trials. Figure S34. Observed/expected SAEs before and after standardization by multimorbidity count: osteoarthritis trials. Figure S35. Observed/expected SAEs before and after standardization by multimorbidity count: osteoporosis trials. Figure S36. Observed/expected SAEs before and after standardization by multimorbidity count: Parkinson’s disease trials. Figure S37. Observed/expected SAEs before and after standardization by multimorbidity count: psoriasis trials. Figure S38. Observed/expected SAEs before and after standardization by multimorbidity count: psoriatic arthropathy trials. Figure S39. Observed/expected SAEs before and after standardization by multimorbidity co [file 12916_2022_2594_MOESM3_ESM.pdf]
