# Supplementary material for: Assessing trial representativeness using serious adverse events: an observational analysis using aggregate and individual-level data from clinical trials and routine healthcare data
Source: BMC Med. 2022 Oct 28;20:410. doi: 10.1186/s12916-022-02594-9 (PMC9615407; doi:10.1186/s12916-022-02594-9)
Supplement: Supplementary file 4 — Additional file 4. Statistical methods. [file 12916_2022_2594_MOESM4_ESM.pdf]

Additional file 4:
Statistical methods

Supplementary methods

The following describes the statistical analyses in greater detail. The numbered headings correspond to the headings in the methods section of the main manuscript. All analyses were conducted in R and all code for these analyses as well as all data (other than individual-level data from the SAIL and trial repositories) is available on the project GitHub repository (https://GitHub.com/dmcalli2/sae_ctg_multicond_public).

Figure S1 gives an overview of the analysis.

1. Comparison of SAEs in trials (aggregate data) and routine care

a. Fitted models to routine care data to estimate SAEs

The following models were fitted within the secure SAIL platform. The outcome was first hospitalisation or death. We fitted generalised linear regression models using a Poisson likelihood and log-link with log person-time used as an offset.

The outcome was modelled separately for each index condition and sex. Prior to running the models, we aggregated the data by summing the total number of first events and the person time observed (until the first event) for each age (in one-year bands). For a Poisson model (assuming rates are constant conditional on the covariates in the model) no information is lost by this aggregation step, but it greatly increased the computational speed.

To allow for non-linearity in age, fractional polynomial terms were included where these improved model fit. The best fitting fractional polynomials (up to two) were chosen using backward selection (this was implemented in the MFP package in R). The model description (separate models for each index condition/sex combination) is shown below:-

$$\log(r_j) = \beta_0 + \beta_1 age_j^a + \beta_2 age_j^b + \log(pt_j)$$

B_0 is the intercept, β_1 and β_2 are the transformed-age coefficients, *pt* is person-time and *j* refers to the row of aggregated data. The superscripts a and b refer to the chosen polynomial transformations for age. a and b were chosen from the set {−2, −1, −0.5, 0, 0.5, 1, 2, 3} with 0 indicating the natural log-transformation. As per Royston, when the same transformation is used for the second fractional polynomial, the covariate is first log-transformed.

The coefficients (vector **β**) and variance-covariance matrix (*V*) for each of these models were then exported from the SAIL platform and are available on the project GitHub repository.

b. Estimated age distribution of aggregate level trial data

For each trial, the proportion of participants in each one-year age-band was assumed to follow a truncated normal distribution. This was chosen because trial eligibility criteria impose hard bounds on the possible values (eg age < 70). We had access to the expectation, variance and upper and lower bounds of age for each trial from clinicaltrials.gov. We are not aware of an analytical method for estimating μ (the central tendency) and σ (the dispersion parameter) of a truncated normal distribution from these values. As such these were estimated using the following approach.

We first estimated the mean and variance based on the observed upper and lower bounds for a grid of values of μ and σ. For both μ and σ we used 0.5 year increments. For μ, the values ranged from the lower bound to the upper bound. For σ the values ranged from 1 to the range (upper bound – lower bound). So for a trial with bounds of 18 and 64 years, for example, the expectation and

variance were estimated for 93 values of μ and 91 values of σ (a total of 8443 combinations of values). The expectation and variance for each value were calculated as follows:-

Standardise upper and lower bounds

$$lowstn = \frac{lower - \mu}{\sigma}$$

$$uppstn = \frac{upper - \mu}{\sigma}$$

Calculate expectation

$$E(x) = \mu + \sigma \times \frac{dnorm(lowstn) - dnorm(uppstn)}{pnorm(uppstn) - pnorm(lowstn)}$$

Calculate variance

$$var(x) = \mu^2 \times$$

$$(1 + \frac{lowstn \times dnorm(lowstn) - uppstn \times dnorm(uppstn)}{pnorm(uppstn) - pnorm(lowstn)} - \left(\frac{dnorm(lowstn) - dnorm(uppstn)}{pnorm(uppstn) - pnorm(lowstn)}\right)^2)$$

Lower and upper refer to the upper and lower bounds, μ and σ refer to the central tendency and dispersion parameter of the truncated normal distribution. dnorm and pnorm are the density and cumulative distribution functions respectively of the normal distribution. We performed these calculations in R code using published on the Quantitative Ecology blog (https://quantitative-ecology.blogspot.com/2009/09/truncated-normal-distribution.html).

We then selected the combined values of μ and σ with the lowest difference between the calculated expectation and standard deviation and the reported expectation and standard deviation.

Next, having selected values for μ and σ we used the cumulative distribution function of the truncated normal distribution to calculate the proportion of participants in each one-year age band (this was a vector of proportions, **p**, with a length equal to the range of ages. The length would be 46 for a trial with ages ranging from 18 to 64 years).

Prior to using this approach, we performed a simulation to both check the accuracy our programming code and to assess the ability of our approach to recover the true central tendency and dispersion from a truncated normal distribution under a range of possible values (for the mean, variance, upper and lower bounds). This simulation showed that this approach accurately recovers the true central tendency and dispersion of the truncated normal distribution. Furthermore, we examined the age distribution of the IPD trials within their respective repositories and found that they consistently followed a truncated normal distribution.

The results of this simulation are shown below:

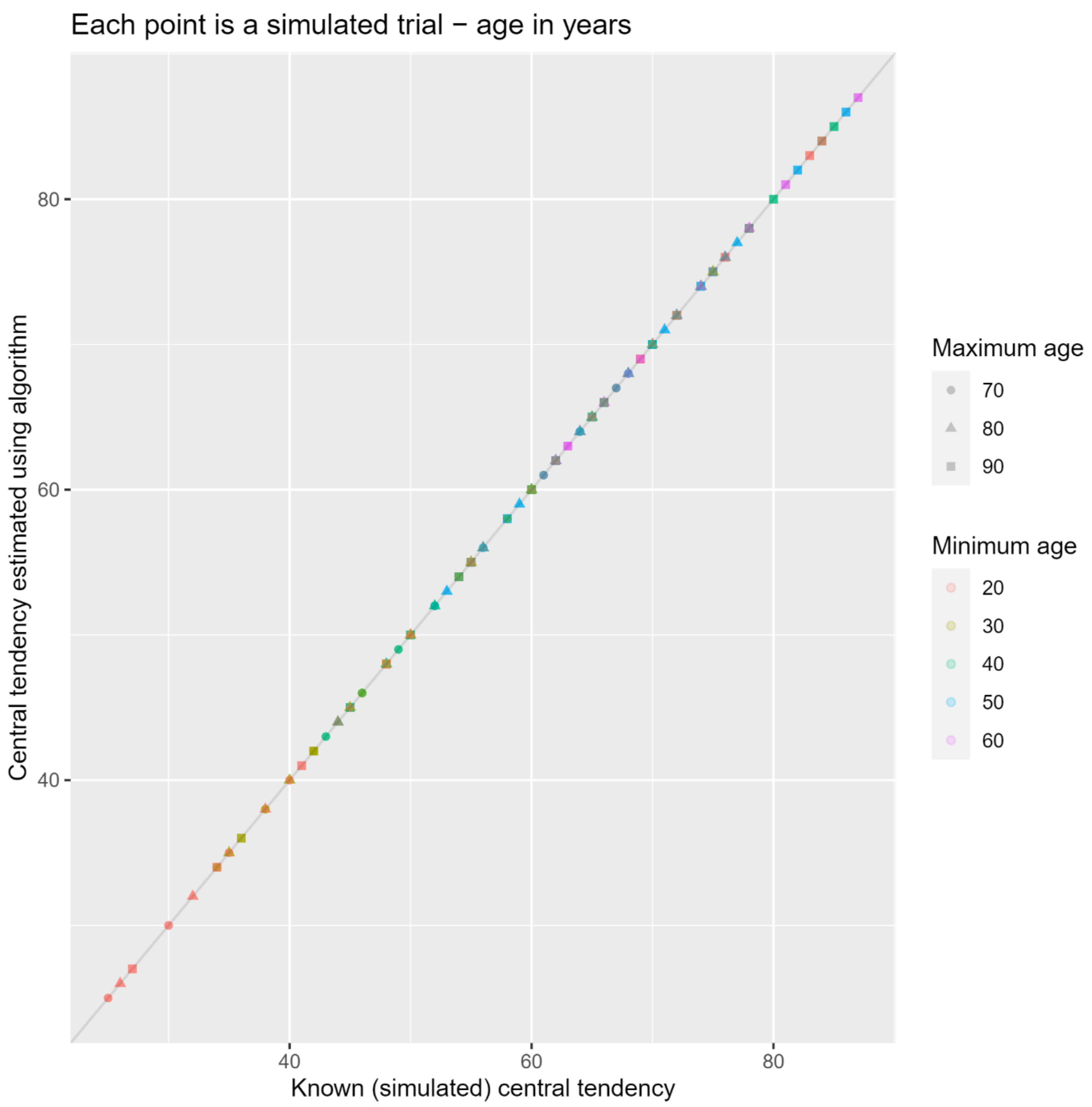
Each point is a simulated trial − age in years
Central tendency estimated using algorithm
Known (simulated) central tendency
80
60
40
40
60
80
Maximum age
70
80
90
Minimum age
20
30
40
50
60

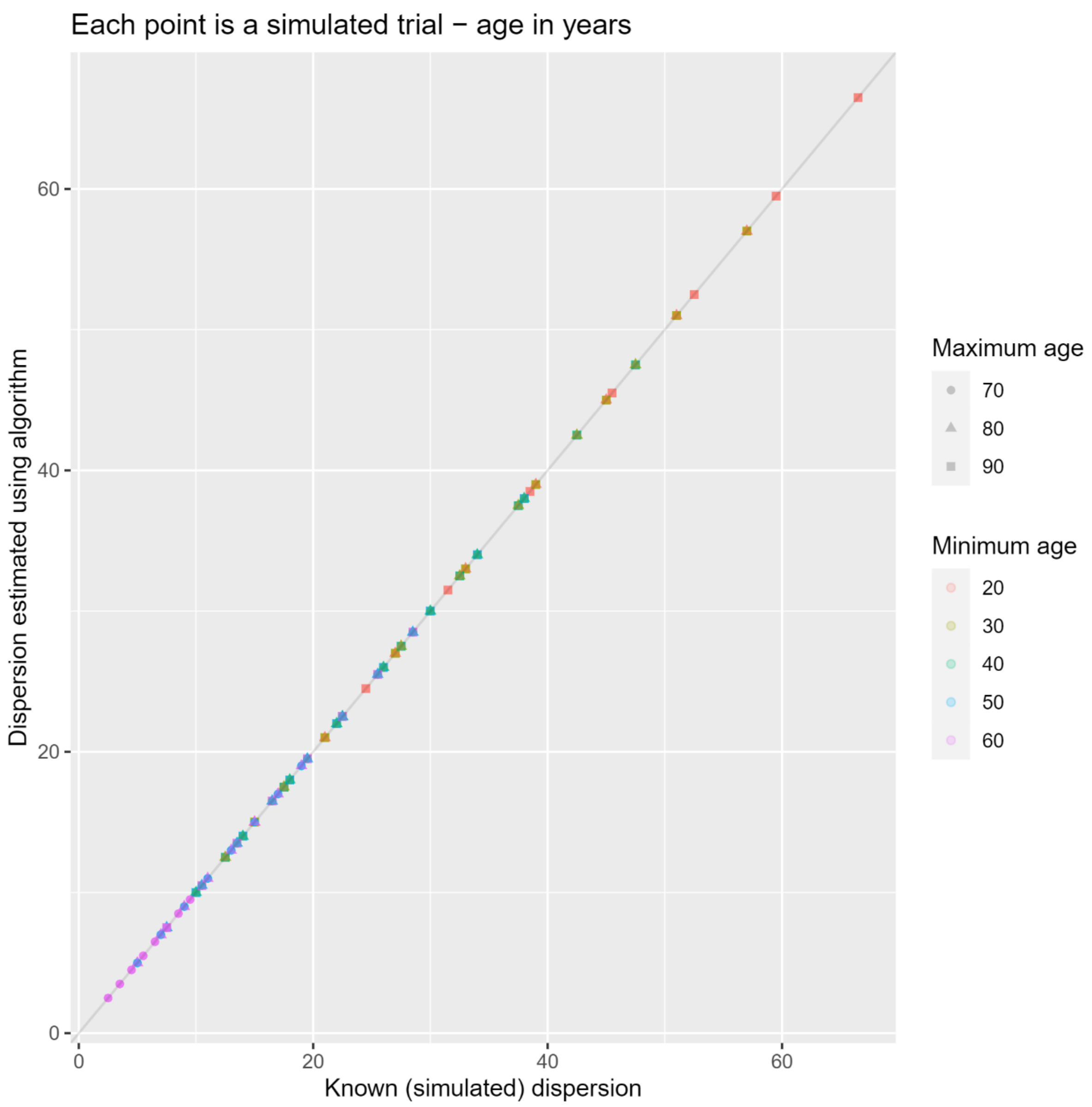

c. Estimated expected SAEs

We used Monte Carlo methods (sampling from probability distributions) to propagate uncertainty in the expected SAE counts (derived from the routine care models (1a) and trial age-sex distributions (1b)) and observed SAE counts (observed in the trials) through to the observed/expected SAE ratios as follows.

For each trial and sex we conducted the following analyses to estimate the expected SAEs:-

i. From the relevant model (index condition and sex-specific), we obtained 10,000 samples from a multivariate normal distribution MVN($\boldsymbol{\beta}$, V). This resulted in a 10,000 by 3 matrix – B – where the columns (j) were the sampled coefficients for β_0, β_1 and β_2 respectively and each row (i) was a set of sampled coefficients.

ii. We applied the relevant fractional polynomial transformations (a-b) to each age band (1b) to obtain an n x 3 matrix - *X* – where n was the number of age-sex bands, the first column was a vector of 1s and the second and third columns were vectors of transformed ages.

iii. We obtained a matrix of 10,000 x n log-rates as follows:-

$$Y = BX^T$$

iv. We exponentiated each element of the matrix *Y* to obtain a matrix of rates – *L*

v. For each element of the matrix *L*, we estimated the risk as follows:-

$$R_{i,j} = 1 - e^{-L_{i,j} \times t}$$

where t was the trial-specific follow up time.

vi. We multiplied *R* by the vector indicating the proportion of participants in each age- band (**p** from 1b) to obtain a vector indicating the overall risk of an SAE (of length 10,000) for all participants in the trial of a given sex:-

$$\boldsymbol{r} = R\boldsymbol{p}^T$$

vii. We multiplied ***r*** by the number of participants of that sex to obtain a vector (of length 10,000) indicating the number of individuals expected to have an SAE, ***e.***

For each trial, we summed each element of the vector of expected SAEs for each sex (***e)*** to obtain a vector (10,000 length) of expected SAEs for the trial.

d. Estimate observed/expected SAE ratio for each trial

To propagate uncertainty in the observed SAE count we did the following. For each trial, we obtained 10,000 samples from a beta distribution where the shape parameters were:-

Shape 1 = number of individuals who experienced an SAE +1

Shape 2 = Total number of participants – number of individuals who experienced an SAE + 1

This resulted in a vector of observed counts, **o**.

We then divided each element of **o** by the expected count from 1c to obtain 10,000 samples of the observed/expected SAE ratio. We summarised this by the 2.5th and 97.5th centiles to obtain an uncertainty range and by the mean to obtain a point estimate.

e. Estimate observed/expected SAE ratio for each index condition

For simplicity, for the index condition level analysis we treated the expected SAE rates as fixed (which were generally estimated with a very high precision due to the large sample size) and fitted the following model to estimate the index condition level observed/expected SAE ratio:-

$$O_z \sim Pois(\lambda_z)$$

$$\log(\lambda_z) = \alpha_z + \log(expected_z)$$

$$\alpha_z \sim N(condition\_mean, \sigma)$$

O is the observed count for each trial, expected the expected count for each trial, λ is the rate parameter for the Poisson distribution, condition_mean is the index condition level estimate for the observed/expected ratio. The models were fit using the rstanarm package (stan_glmer function) using the default weakly informative priors for condition_mean and σ (http://mc-stan.org/rstanarm/articles/priors.html).

2. Associations between multimorbidity count and SAEs in trials (IPD) and routine care

a. Fit models for multimorbidity and SAE rates in routine care data

We replicated the modelling described in 1a, except that we additionally included the morbidity count in the aggregation and model fitting steps. The model is described below. To distinguish the model from the one fitted in 1a, we have used a different symbol for the coefficients:-

$$\log(r_j) = \gamma_0 + \gamma_1 age_j^a + \gamma_2 age_j^b + \gamma_3 morbidity_j^c + \gamma_4 morbidity_j^d + \log(pt_j)$$

γ with subscripts 0, 1, 2, 3 and 4 refer to the coefficients for the intercept, age (1st transformation), age (2nd transformation), morbidity count (1st transformation) and morbidity count (2nd transformation), and the superscripts c and d are the 1st and 2nd fractional polynomial transformations of morbidity count with the other terms being the same as those in 1a.

The resulting coefficients (**γ**) and variance-covariance matrix (*Z)* were exported from SAIL for subsequent analyses and are available in the project GitHub repository.

b. Calculate log rate ratio for multimorbidity counts in routine care

We used the approach described in Royston et al (Royston P, Ambler G, Sauerbrei W. The use of fractional polynomials to model continuous risk variables in epidemiology. Int J Epidemiol. 1999 Oct;28(5):964–74.) to calculate the log-rate ratios and standard error for models with second-degree fractional polynomials (γ_3 and γ_4). We estimated the rate ratios and standard errors across a range of multimorbidity counts, from 0 to 5 in 0.1-unit increments) and plotted these (Figure 3).

c. Fit models for multimorbidity and SAE rates in trial IPD

Within the trial repository we estimated the association between SAEs and morbidity count as follows. This analysis was limited to trials with IPD and where the total number of SAEs per sex was ≥20 (n=60 trials for 11 index conditions). Given the smaller number of events within each trial, we only modelled the morbidity count as a linear term.

$$\log(r_j) = \gamma_0 + \gamma_1 age_j^a + \gamma_2 age_j^b + \gamma_3 morbidity_j + \log(pt_j)$$

We exported the coefficient and standard error for γ_3 from the trial repositories.

d. Meta-analyse morbidity models

For each index condition, within each sex, we meta-analysed the effect measure estimates for the morbidity SAE association (γ_3). We fitted a random effects meta-analysis via restricted maximum-likelihood estimation within the metafor package in R. This assumes that the log rate ratio is normally distributed. We subsequently plotted the overall estimate and 95% confidence interval for each sex/index condition across the range of comorbidity counts observed for trials with that index condition (Figure 3).

3. Comparison of observed and expected trial SAEs before and after accounting for multimorbidity

The following analysis was very similar to that conducted in 1, but there were differences due to the addition of morbidity count data, access to trial IPD and the fact that the trial IPD was held in a secure safe haven from which individual-level data could not be exported.

a. Fitted models to routine care data to estimate SAEs

Model fitting is described in 2a.

b. Age-sex-morbidity count distribution of aggregate level trial data

Unlike 1b we had IPD for these trials. Therefore, for each trial we used individual-level data with which we observed the age, sex and morbidity count for each individual.

c. Estimated rate of SAEs for each trial participant according to age, sex, and morbidity count

We then applied the coefficients and variance covariance matrix (3a) to the individual-level data (3b) to estimate the expected counts. We sampled from a multivariate normal distribution to allow us to propagate uncertainty from the routine care models through to the final estimates of expected counts as follows:-

For each relevant selected index condition and sex we conducted the following analyses to estimate the expected SAEs:-

i. From the relevant model, we obtained 10,000 samples from a multivariate normal distribution MVN($\boldsymbol{\gamma}$, Z). This resulted in a 10,000 by 5 matrix – B – where the columns (j) were the sampled coefficients for γ_0, γ_1, γ_2 , γ_3 and γ_4 respectively and each row (i) was a set of sampled coefficients.
ii. We applied the relevant fractional polynomial transformations (a-d) to each individual age and morbidity count (1b) to obtain an n x 5 matrix - X – where n was the number of participants, the first column was a vector of 1s, the second and third columns were vectors of transformed ages and the fourth and fifth columns were vectors of transformed morbidity counts.
iii. We obtained a matrix of 10,000 x n log-rates as follows:-

$$Y = BX^T$$

iv. We exponentiated each element of the matrix Y to obtain a matrix of rates – L
v. For each element of the matrix L, we estimated the risk as follows:-

$$R_{i,j} = 1 - e^{-L_{i,j} \times t}$$

where t was the trial-specific follow up time.

vi. We subset R into columns where participants were male and female.
vii. We summed the each of the rows in the subset matrices to obtain vectors of the expected counts in men and women**.**
viii. We plotted these counts using Q-Q plots and histograms, identifying that the trial-sex-level expected counts were approximately normally distributed then exported the mean and standard deviation of these counts from the trial repositories.
ix. Outside the trial repository we obtained 10,000 samples from these trial-sex specific normal distributions (viii) and summed each element of these to obtain a vector of expected counts for each trial.
x. We summed the observed SAEs for each sex within each trial and exported these counts from the trial repositories.

d. Estimate observed/expected ratio

For each trial and sex, we obtained 10,000 samples from a beta distribution where the shape parameters were:-

Shape 1 = number of individuals who experienced an SAE (3c+1

shape 2 = Total number of participants – number of individuals who experienced an SAE + 1

Within each trial, we summed each element of the vector for each sex in order to obtain a vector of observed counts, **o**.

We then divided each element of **o** by the expected count from 3c.ix to obtain 10,000 samples of the observed/expected SAE ratio. We summarised this by the 2.5th and 97.5th centiles to obtain an uncertainty range and by the mean to obtain a point estimate

We repeated the above analyses after dropping morbidity count (model described in 1a).

Figure S1 Overview of analysis

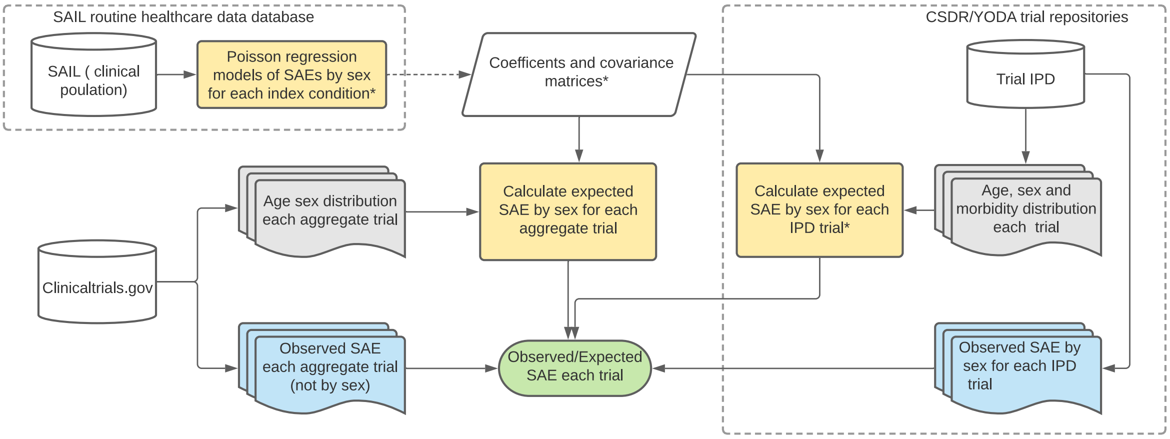

* i) for age alone and ii) for age and morbidity count

Note that for the usual situation – the evaluation of aggregate-level published trial SAE data – there is no need to access trial IPD.
